# *CDKN2A* homozygous deletions and *TSC2* somatic mutations in metastatic pancreatic neuroendocrine tumors

**DOI:** 10.1038/s41698-025-01210-2

**Published:** 2025-12-05

**Authors:** Tito Teles Jesus, Lorenzo Ferrando, Lia Rodrigues, Rui Sousa Martins, Luís Cardoso, José Manuel Lopes, Paula Soares, Arnaud Da Cruz Paula, João Vinagre

**Affiliations:** 1 Instituto de Investigação e Inovação em Saúde (i3S), Universidade do Porto, 4200-135 Porto, Portugal; 2 Instituto de Patologia e Imunologia Molecular da Universidade do Porto (Ipatimup), 4200-135 Porto, Portugal; 3 Department of Internal Medicine and Clinical Specialties, University of Genoa, Genoa, Italy; 4 Faculdade de Medicina da Universidade do Porto (FMUP), 4200-319 Porto, Portugal; 5 Departamento de Endocrinologia, Diabetes e Metabolismo do Centro Hospitalar Universitário de Coimbra, 3000-075 Coimbra, Portugal

**Keywords:** Cancer genomics, Neuroendocrine cancer, Pancreatic cancer

## Abstract

Despite improvements in the molecular profiling of pancreatic neuroendocrine tumors (PanNETs), predicting their clinical behavior and response to specific therapies remains challenging. We sought to elucidate the molecular basis underlying the broad phenotypic variations in these neoplasms through a genetic characterization of primary and metastatic PanNETs. Our findings revealed an enrichment of *CDKN2A* homozygous deletions and *TSC2* somatic mutations in metastatic PanNETs when compared to non-metastatic lesions. Tumor evolution analysis further revealed the acquisition of such genetic alterations as late events in the progression of these neoplasms, conferring poor survival outcomes to the affected patients. Biallelic loss of DNA damage repair genes, *ATRX* and/or *DAXX*, was associated with a high fraction of the genome altered in PanNETs, with pathogenic alterations affecting those genes also being associated with a homologous recombination deficiency signature. These findings highlight molecular mechanisms driving PanNET progression and underscore the need for further molecular characterization and tumor evolution studies to evaluate targeted therapies for such a challenging disease.

## Introduction

Pancreatic neuroendocrine tumors (PanNETs) are low-incidence diseases accounting for less than 3% of all pancreatic malignancies, but their prevalence is currently rising^1^. Patients with PanNET present metastases at diagnosis in 60–80% of cases^1^, and their prognosis differs widely, with some tumors having an indolent nature, with a reasonable length of survival even with a metastatic presentation, and others being extremely aggressive with a poor prognosis^2^. Recent advances in sequencing technologies have uncovered the molecular basis of numerous cancers and led to new prognostic classification systems and actionable targets^3^. Indeed, it has been demonstrated that increasing numbers of molecular pathways are involved in the biology and clinical behavior of PanNETs, such as DNA damage repair, chromatin remodeling, telomere alteration, PI3K/AKT/mTOR and p53/cell cycle signaling pathways^3–5^. DNA sequencing analysis of PanNETs has identified recurrent somatic mutations affecting *MEN1*, *DAXX*, and *ATRX*^3–6^. Loss of function of *DAXX* or *ATRX*, results in telomere dysfunction, due to the activation of homologous recombination in telomeric DNA, and consequently leads to genomic instability. The inactivation of these genes is also implicated in the activation of the alternative lengthening of telomeres (ALT) mechanism^3,6,7^. Additional mutations in the PI3K/AKT/mTOR pathway were also found in these neoplasms, such as those affecting *TSC1/2* and *PTEN* tumor suppressor genes, and pronounced losses of these genes were correlated with liver metastases, shorter time to progression, and shorter disease-free and overall survival^5,6,8^. More recently, high frequencies of *CDKN2A* copy number (CN) losses were observed in metastatic PanNETs^9^. Despite improvements in molecular profiling and prognostic grading and staging systems, it remains a challenge to predict the clinical behavior of PanNETs and the response to specific therapies, given the high degree of heterogeneity of these tumors. While most PanNETs present as advanced disease, the available systemic therapies provide modest benefits. Therefore, there is a particular need to develop more effective systemic therapies based on the molecular profile of PanNETs. Specifically, determining the molecular basis of the wide phenotypic variations underlying this disease is crucial to develop future personalized therapies.

In an attempt to meticulously characterize the genetic repertoire and unravel clinically informative alterations affecting PanNETs, we analyzed the molecular features of 192 primary PanNETs and metastases from Nguyen et al.^10^, 6 primary tumors with matched metastases from Cowzer et al.^11^ and Jee et al.^12^, and 33 primary PanNETs from the Pan-Cancer Analysis of Whole Genomes (PCAWG)^13^.

## Results

### Clinical features of pancreatic neuroendocrine tumors

Tumors from 211 patients subjected to targeted massively parallel sequencing via MSK-IMPACT from 2014 to 2020 were identified^10^. Nineteen cases were excluded due to the absence of any genetic alterations, leaving 192 cases in the study cohort, including 87 primary tumors and 105 unmatched metastases. The median age of primary and metastatic tumors was of 58 and 59 years, respectively (range of primaries, 26-86 years; range of metastases, 28-82 years; Supplementary Table S1). Of the 87 primary PanNETs and 105 metastases, 14 (16%) and 50 patients (48%) died of the disease, respectively. The median follow-up time of the primary tumors was of 30.9 months (range 0.92–70.7 months), and of the metastases was 17.1 months (range 0.46-71.9 months; Supplementary Table S1). Of the 6 patients with primary tumors and matched metastases, 3 died of the disease^11,12^.

### Genetic characterization of primary and metastatic pancreatic neuroendocrine tumors

**Fig. 1:**
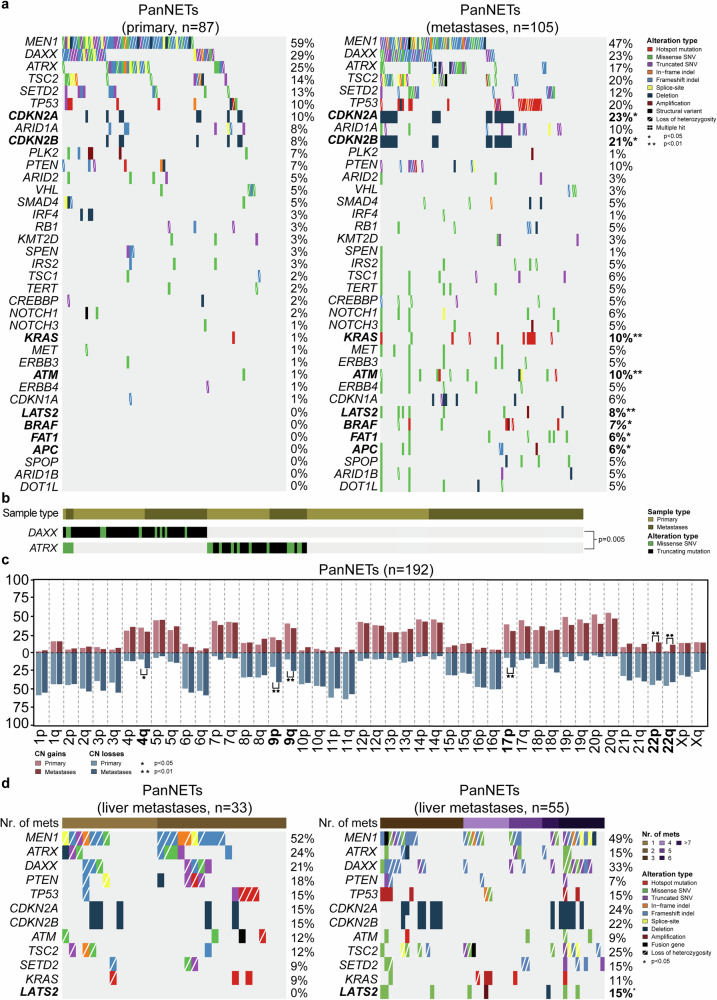
Genomic features of primary neoplams and metastases from pancreatic neuroendocrine tumors. Comparisons between primary neoplams and metastases from pancreatic neuroendocrine tumors (PanNETs) for **a**, recurrent somatic alterations, **b**, mutual exclusivity analysis between *DAXX* and *ATRX*, and **c**, copy number gains and losses. Comparisons between liver metastases from PanNETs stratified according to the number of distant metastases for **d**, recurrent somatic alterations. Alteration types and number of metastases are color-coded according to the legend. Statistical significance was evaluated using Fisher’s exact test. CN copy number, PanNETs pancreatic neuroendocrine tumors.

We next assessed the genetic differences between primary tumors and metastases from PanNETs (Fig. 1). No statistically significant differences were observed regarding the tumor mutational burden (TMB) and fraction of the genome altered (FGA) between the metastases and primary tumors (2.59 vs. 2.94, *p* = 0.052; 37.3% vs. 47.7%, *p* = 0.316, respectively; Supplementary Fig. S1a, b). Genetic alterations were found to be significantly more frequent in the metastases when compared to primary tumors, such as those affecting *CDKN2A* (23% vs. 10%, *p* < 0.05), *CDKN2B* (21% vs. 8%, *p* < 0.05), *KRAS*, *ATM* (10% vs. 1%, *p* < 0.01, for both genes), *LATS2* (8% vs. 0%, *p* < 0.01), *BRAF* (7% vs 0%, *p* < 0.05), *FAT1* and *APC* (6% vs 0%, *p* < 0.05, for both genes) (Fig. 1a). As previously reported in PanNETs^3,6^, *DAXX* and *ATRX* genetic alterations were found to be mutually exclusive (*p* = 0.005; Fig. 1b). While the majority of the *DAXX* mutations were deemed clonal, a substantial proportion of mutations affecting *ATRX* were found to be subclonal (9/38, 24%), mainly in the primary tumors (6/22, 27%; Supplementary Fig. S1c, Supplementary Table S2). When looking at CN alterations, the frequency of gains of chromosome 22p and 22q was significantly higher in the metastases than in primary tumors (15.2% vs 2.3% and 12.4% vs 2.3%, respectively, *p* < 0.01; Fig. 1c). In addition, loss of chromosome 4q was found to be significantly more frequent in the metastases when compared to primary tumors (21% vs 9%, *p* < 0.05), as were the losses of chromosomes 9p and 9q (40% vs. 20%, and 24.8% vs. 9.2%, respectively, *p* < 0.01; Fig. 1c).

We have also stratified the primary PanNETs according to the presence/absence of metastases. Such stratification revealed a significantly higher TMB in primary tumors with evidence of metastases than in primary tumors with no metastases (2.59 vs 0.98, *p* = 0.018; Supplementary Fig. S2a). No statistically significant differences regarding the FGA was observed between the former and the latter (45.6% vs 26.3%, *p* = 0.082; Supplementary Fig. S2b). When looking at the recurrent somatic mutations from both the MSK-IMPACT^10^ and Scarpa et al.^6^ data however, we observed a statistically significant enrichment of mutations affecting *DAXX*, *ATRX*, *TSC2*, *TP53* and *ARID1A* in primary tumors with evidence of metastasis when compared to primary tumors with no metastasis (33% vs 19%, 25% vs 9%, 11% vs 3%, 10% vs 2%, and 8% vs 1%, respectively, all statistical differences at *p* < 0.05; Supplementary Fig. S2c). Furthermore, we observed in primary PanNETs a significantly higher frequency of gains of chromosomes 7 (50% vs. 17.6%, *p* < 0.05) and 14 (52.9% vs. 23.5%, *p* < 0.05), and losses of chromosomes 10p (50% vs. 17.6%, *p* < 0.05), 10q (52.9% vs 17.6%, *p* < 0.05), 21p (37.1% vs 11.8%, *p* < 0.05) and 21q (40% vs 11.8%, *p* < 0.05; Supplementary Fig. S2d).

Most of the metastases from PanNETs were found in the liver (84%; Supplementary Table S1). When stratifying the liver metastases according to the number of metastases each corresponding patient harbors, we noticed a significantly higher frequency of *LATS2* genetic alterations in the metastases from patients harboring more than 2 metastases when compared with those harboring 2 metastases or less (15% vs 0%, *p* < 0.05; Fig. 1d).

### Biallelic loss of *DAXX* or *ATRX* contributes to an increased fraction of the genome altered in pancreatic neuroendocrine tumors

**Fig. 2:**
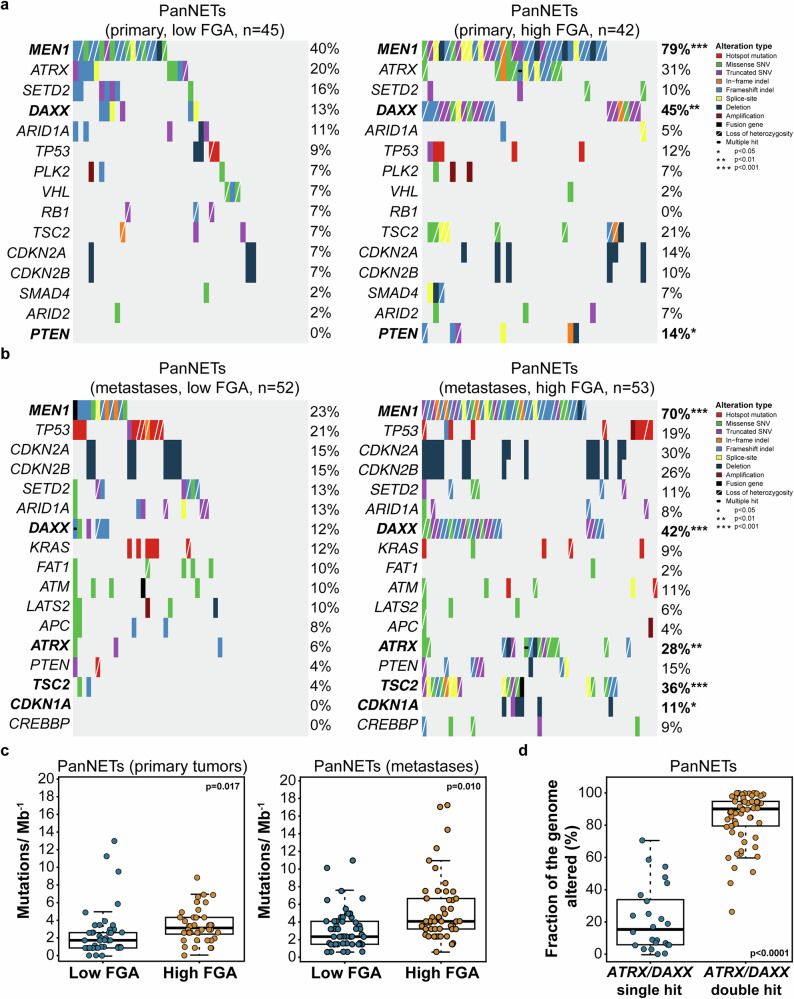
Genomic features of primary neoplams and metastases from pancreatic neuroendocrine tumors with low and high fraction of the genome altered. Comparisons between primary neoplasms and metastases from pancreatic neuroendocrine tumors (PanNETs) with low and high fraction of the genome altered for **a** and **b**, recurrent somatic alterations, and **c**, tumor mutational burden. Comparisons between PanNETs harboring single-hit alterations affecting *DAXX* and *ATRX*, and double genetic alterations affecting the same genes for **d**, fraction of the genome altered. Alteration types are color-coded according to the legend. Statistical significance was evaluated in (**a**) and (**b**) using Fisher’s exact test, and in (**c**) and (**d**) using the Mann-Whitney U test. FGA, fraction of the genome altered; PanNETs, pancreatic neuroendocrine tumors.

When looking at the CN profiles of both primary tumors and metastases, we noticed two main sample groups: one containing extremely high numbers of CN alterations and another harboring samples with relatively quiet genomes (Supplementary Fig. S3a, b). Considering that PanNETs often present high levels of genetic instability at the chromosomal level^6^, we sought to compare the molecular features of primary and metastatic PanNETs with low FGA, with primary and metastatic PanNETs with high FGA, respectively (Fig. 2). In comparison to primary tumors with low FGA, primary tumors displaying high FGA were found to harbor significantly higher frequencies of *MEN1* (40% vs. 79%, *p* < 0.001), *DAXX* (13% vs 45%, *p* < 0.01) and *PTEN* (0% vs 14%, *p* < 0.05) genetic alterations, with the latter harboring a substantial proportion of subclonal mutations (Fig. 2a, Supplementary Fig. S4a, Supplementary Table S2). The metastases with high FGA were found to have significantly higher frequencies of genetic alterations than metastases displaying low FGA, such as those affecting *MEN1* (70% vs. 23%, *p* < 0.001), *DAXX* (42% vs. 12%, *p* < 0.001), *TSC2* (36% vs. 4%, *p* < 0.001), *ATRX* (28% vs. 6%, *p* < 0.01), and *CDKN1A* (11% vs. 0%, *p* < 0.05; Fig. 2b). Of note, while only 6% (2/35) of the mutations affecting *DAXX* and *ATRX* were found to be subclonal in the metastases with high FGA, 44% (4/9) of mutations affecting these genes were deemed subclonal in the metastases with low FGA (Supplementary Fig. S4b, Supplementary Table S2).

A significantly higher TMB was observed in both primary tumors and metastases with high FGA than in primary tumors and metastases with low FGA (primary tumors, 3.3 vs 1.72, *p* = 0.017; metastases: 3.5 vs 1.84, *p* = 0.01; Fig. 2c). We noticed that loss of heterozygosity (LOH) followed the majority of *DAXX* and *ATRX* genetic alterations in both primary tumors and metastases displaying high FGA (Fig. 2a, b). Compared to single alterations affecting *DAXX* or *ATRX*, samples with double genetic alterations affecting one of these two genes harbored a significantly higher FGA (15.3% vs 90%, *p* < 0.0001; Fig. 2d).

**Fig. 3:**
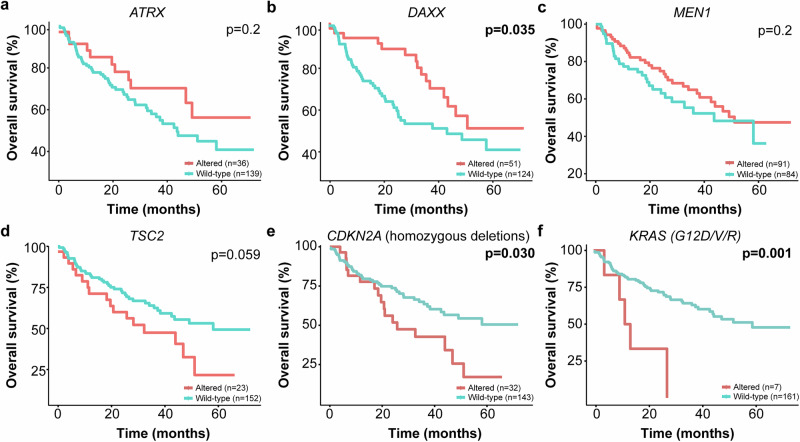
Survival analysis of metastatic pancreatic neuroendocrine tumors harboring genetic alterations affecting *ATRX, DAXX, MEN1, TSC2, CDKN2A* and *KRAS.* Kaplan-Meier overall survival curves for 175 pancreatic neuroendocrine tumors stratified by altered gene status, namely **a**, *ATRX*, **b**, *DAXX*, **c**, *MEN1*, **d**, *TSC2*, **e**, *CDKN2A* (homozygous deletions), **f**, *KRAS* (G12D/V/R). Statistical significance was evaluated in (**a**), (**b**), (**c**), (**d**), (**e**) and (**f**) using the log-rank test. PanNETs, pancreatic neuroendocrine tumors.

When looking at the survival curves, however (Fig. 3a, b), we noticed that metastatic patients harboring *ATRX* or *DAXX* genetic alterations had better survival outcomes than patients not harboring genetic alterations affecting these two genes, with this difference being statistically significant only for *DAXX* (*p* = 0.035; Fig. 3b). When stratifying the patients with biallelic loss of *ATRX* or *DAXX* according to their survival status, we found that 64% of patients who died from the disease harbored mutations in the *TSC2* gene, being this difference statistically significant in comparison with the alive patients (64% vs 26%, *p* < 0.05; Supplementary Fig. S4c). Homozygous deletions of *CDKN2A* and *CDKN2B* genes were also more prevalent in deceased patients, though not statistically significant (50% vs 32%, *p* > 0.05; Supplementary Fig. S4c).

### *CDKN2A* homozygous deletions and *TSC2* genetic alterations confer poor survival outcomes to the affected patients and are late events in the malignant progression of pancreatic neuroendocrine tumors

Considering the overall survival results for patients harboring *ATRX* or *DAXX* genetic alterations, we focused our attention on other altered cancer-related genes in metastatic PanNETs (Fig. 3c–f). Interestingly, we found that metastatic patients harboring *TSC2* genetic alterations, as well as *CDKN2A* homozygous deletions and *KRAS* hotspot codon mutations (*G12D/V/R*) had worse survival outcomes when compared with patients not harboring *TSC2*, *CDKN2A* or *KRAS* genetic alterations, being this difference statistically significant for both *CDKN2A* and *KRAS* affected genes (*CDKN2A* homozygous deletions, *p* = 0.030, *KRAS G12D/V/R*, *p* = 0.001; Fig. 3e, f).

**Fig. 4:**
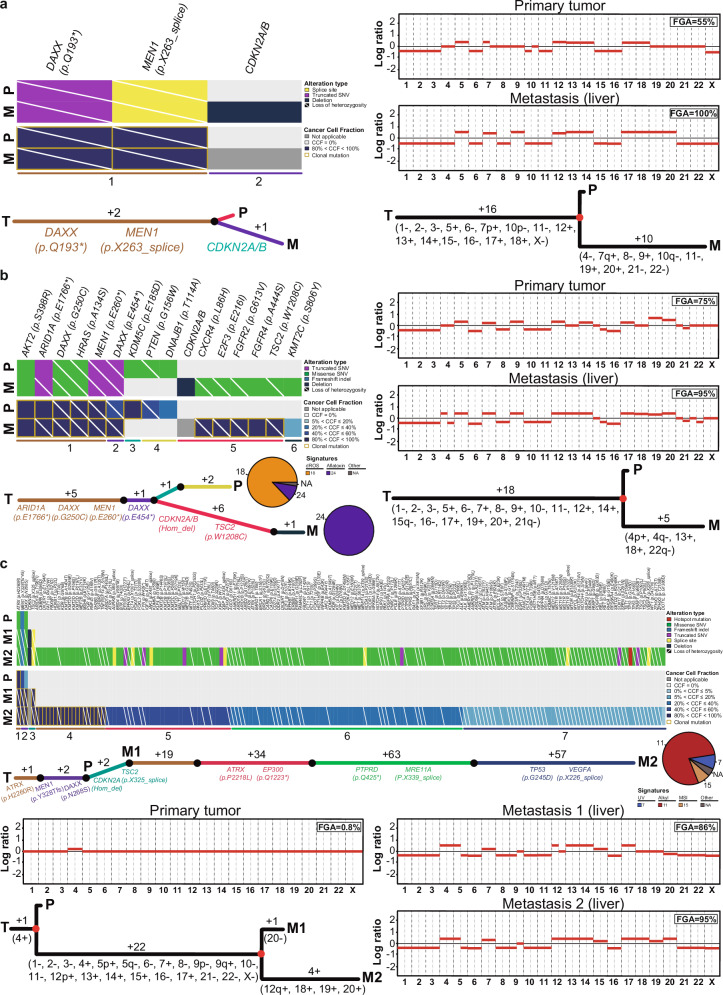
Clonal composition and phylogenetic analysis of primary pancreatic neuroendocrine tumors and matched metastases. Clonal composition and phylogenetic analysis of three cases (**a**–**c**). Heatmaps depicting the frequency of genetic alterations and cancer cell fractions of each somatic mutation in the primary pancreatic neuroendocrine tumors (PanNETs) and matched metastasis of a given case are shown ((**a**) and (**b**), top left; (**c**), **top**). The alteration types and cancer cell fraction are color-coded according to the legend. Copy number plots depicting segmented Log2 ratios (y-axis) according to genomic position (x-axis) of primary PanNETs and matched metastases are depicted ((**a**) and (**b**), top right; (**c**), bottom). Phylogenetic trees of primary PanNETs and matched metastases are shown ((**a**) and (**b**), bottom left; (**c**), middle). Trunk and branches are colored according to clusters, and the number of somatic mutations that result in the divergence of a clone/subclone from its ancestor is shown. Pathogenic mutations that define a given clone are depicted. Phylogenetic trees based on copy number alterations are shown ((**a**) and (**b**), bottom right; (**c**), bottom left). The numbers alongside the branches represent the number of copy number alterations. Gains and losses are shown in parentheses. Alkyl alkylating agent, dROS damage by reactive oxygen species, M metastasis, MSI microsatellite instability, NA not applicable, P primary, UV ultra-violet light, T trunk.

To further validate our previous findings, we retrieved the genomic data from six patients with primary PanNETs and matched metastases^11,12^. Except for one case, pathogenic mutations affecting *MEN1*, *DAXX* or *ATRX* were found to be clonal and at the trunk of the mutational phylogenetic trees (Fig. 4a–c, Supplementary Fig. 5a, c, Supplementary Table S3). Primary tumors and metastases with biallelic losses of *ATRX* or *DAXX* displayed extremely high FGA (Fig. 4a–c, Supplementary Fig. 5a). The sole case with one primary tumor and two matched metastases had the three components sharing three somatic mutations, including a clonal *ATRX* missense mutation, a *MEN1* frameshift mutation, and a *DAXX* missense mutation (both mutations deemed subclonal in the primary tumor and becoming fully clonal in the metastases; Fig. 4c, Supplementary Table S3). Loss of heterozygosity affecting the three genes was found to occur only in the two metastases, with a marked increase of the FGA from the primary tumor to the first metastasis (Fig. 4c). The second metastasis was found to harbor 19 clonal and 154 subclonal mutations, including a subclonal *TP53* hotspot mutation (Fig. 4c, Supplementary Table S3). Such high TMB can be explained by the treatment with alkylating agents and the corresponding dominant signature 11 observed in this component, as described by Cowzer et al.^11^ (Fig. 4c). Acquisition of *CDKN2A* homozygous deletions and *TSC2* mutations was found to occur in three and in two metastatic components, respectively (Fig. 4a–c, Supplementary Table S3). Hotspot mutations affecting *CDKN2A* and *TP53* were also found to affect the metastatic component of an additional two cases (Supplementary Fig. 5b, c, Supplementary Table S3).

### Genetic alterations affecting *ATRX* and *DAXX* are associated with homologous-recombination deficiency features in primary pancreatic neuroendocrine tumors

**Fig. 5:**
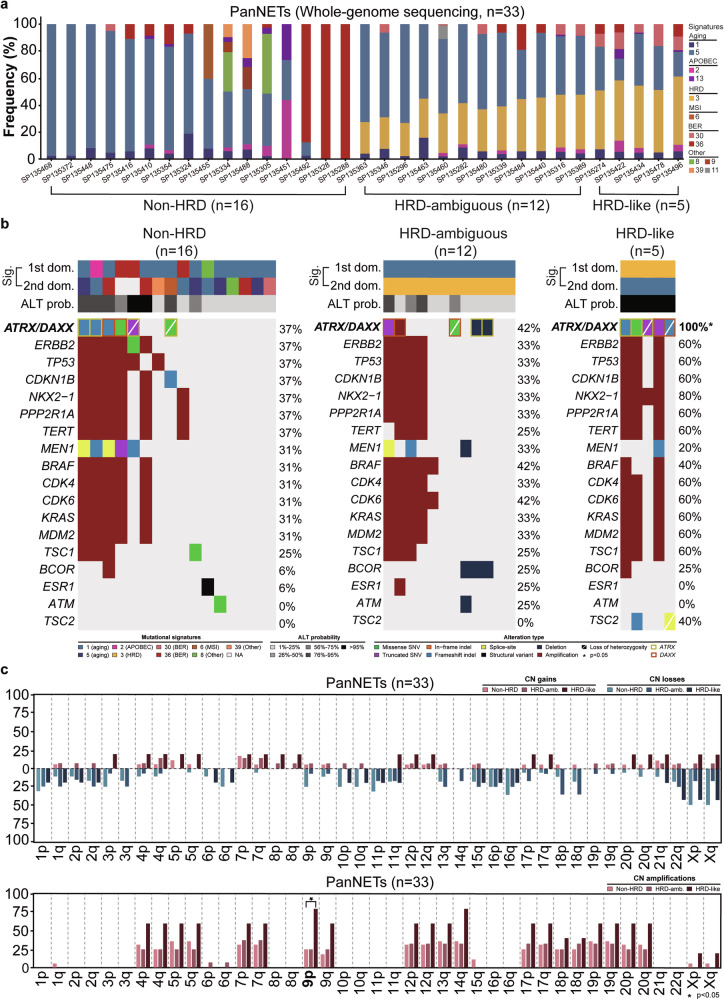
Genomic features of primary pancreatic neuroendocrine tumors with and without homologous recombination deficiency features. Comparisons between primary pancreatic neuroendocrine tumors stratified according to the presence/absence of homologous recombination deficiency features assessed by mutational signatures (**a**) for **b**, recurrent somatic mutations and **c**, copy number alterations (gains, losses, and amplifications). Alteration types are color-coded according to the legend. Statistical significance was evaluated using Fisher’s exact test. ALT, alternative lengthening of telemores; BER base-exchange repair, CN copy number, HRD homologous recombination deficiency, MSI microsatellite instability, PanNETs pancreatic neuroendocrine tumors.

To further characterize which PanNET display a homologous recombination deficiency (HRD) feature, we next looked at the mutational signatures inferred from somatic mutations of 33 primary PanNETs subjected to whole-genome sequencing (WGS)^13^ and performed a genetic comparison between PanNETs with no evidence of HRD features and with PanNETs with patterns of HRD (Fig. 5). Mutational signatures inferred from somatic mutations of these 33 primary PanNETs revealed the presence of 16 PanNETs with no mutational signature 3 exposure (“non-HRD” group), 12 PanNETs harboring a second dominant mutational signature 3 (“HRD-ambiguous” group), and 5 PanNETs with a dominant mutational signature 3 (“HRD-like” group; Fig. 5a). When looking at the genetic repertoire of these three groups, we observed a step-wise increment of the frequency of genetic alterations affecting *ATRX* and *DAXX* from the “non-HRD” to the “HRD-like” group, with the latter having a higher frequency of these alterations when compared to the “HRD-ambiguous” (100% vs 42%, *p* = 0.029) and “non-HRD” (100% vs 37%, *p* = 0.035) groups (Fig. 5b). As expected, the ALT probability observed in the HRD dominant cases was extremely high (>95%; Fig. 5b). Of note, the “HRD-like” group also harbored *TSC2* pathogenic mutations affecting 40% of patients, with these alterations being absent in both the “HRD-ambiguous” and “non-HRD” groups (Fig. 5b).

When looking at CN alterations, we observed that between 25-80% of all 33 PanNETs harbored amplifications affecting chromosomes 4, 5, 7, 9, 12, 13q, 14q, 17, 18, 19, and 20, with chromosome 5 being one of the the most affected chromosome, as seen by the frequency of *TERT* gene amplifications present in 36% of all cases (Fig. 5b, c). In addition, a significantly higher frequency of amplifications of chromosome 9p was observed in the “HRD-like” tumors when compared to “non-HRD” PanNETs (80% vs 25%, *p* < 0.05; Fig. 5c).

### Pancreatic neuroendocrine tumors harbor recurrent somatic genetic alterations affecting genes in the PI3K/AKT/mTOR, p53, and DNA damage repair pathways

**Fig. 6:**
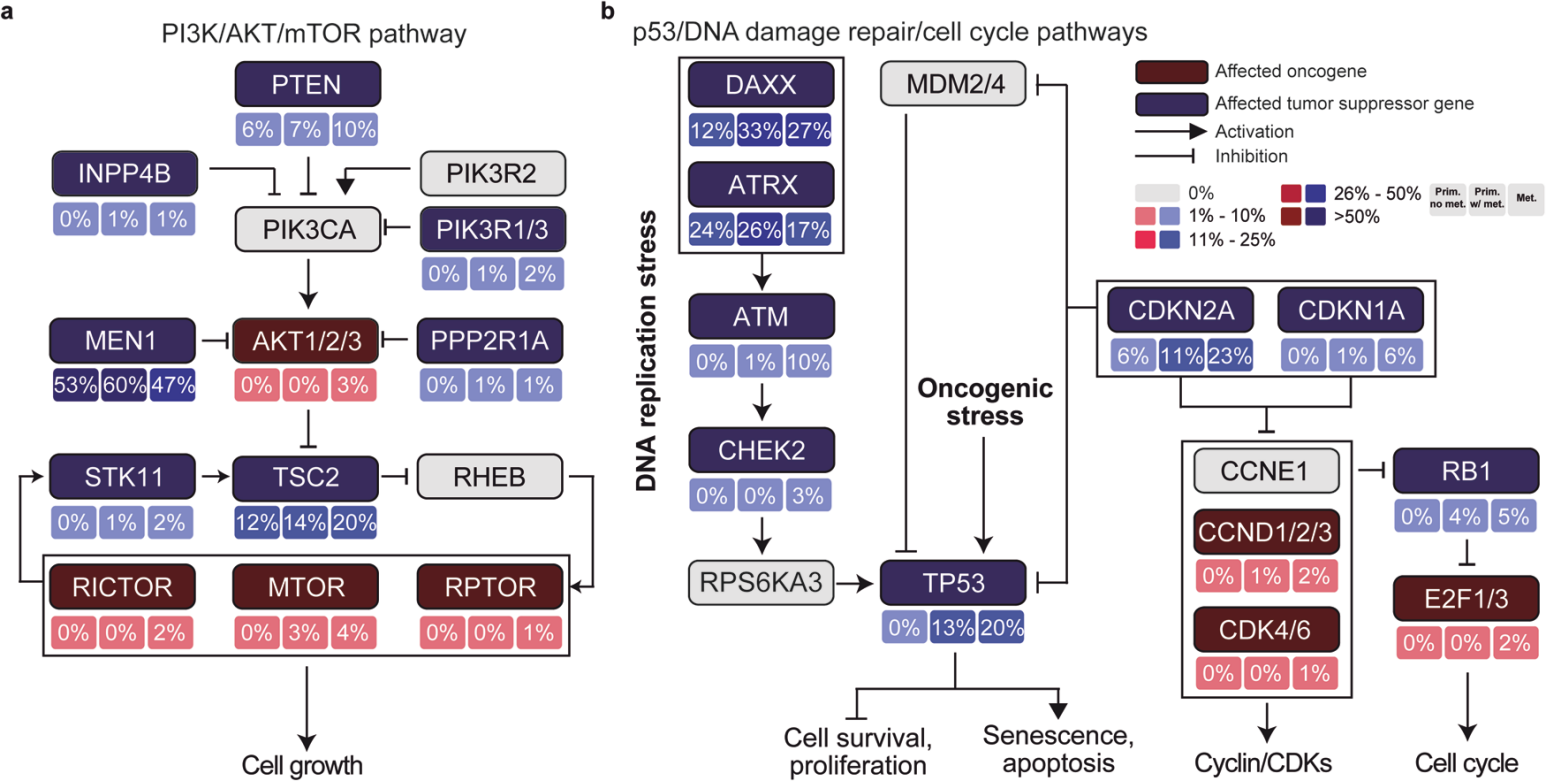
Signaling pathways most affected by genetic alterations present in pancreatic neuroendocrine tumors. Frequency of activating (red) or loss-of-function (blue) somatic genetic alterations affecting genes in pancreatic neuroendocrine tumors (PanNETs) and present in the canonical **a** PI3K/AKT/mTOR, and **b** p53/DNA damage repair/cell cycle signaling pathways. The frequency of primary PanNETs with no evidence of metastases, primary tumors with evidence of metastases, and metastatic samples is depicted under the gene name. Statistical significance was evaluated using using Mann-Whitney U test.

Given the fact that the most affected genes in PanNETs are located in distinct signaling pathways, we performed a pathway analysis based on the somatic mutations, structural variants, CN amplifications, and homozygous deletions present in the 192 PanNETs included in this study. Such analysis revealed an enrichment of genetic alterations targeting the PI3K/AKT/mTOR, DNA damage repair, and p53/cell cycle pathways (Fig. 6a, b). The most affected gene was the tumor suppressor gene *MEN1*, with half of the patients harboring *MEN1* genetic alterations. The frequency of genetic alterations affecting *TSC2*, an additional tumor suppressor gene, was also found to be high, mainly in the metastases (20%; Fig. 6a). Both tumor suppressor genes, *DAXX* and *ATRX* were found to affect 46% of all cases, with a higher prevalence in primary tumors with evidence of metastases (Fig. 6b). PanNETs harboring such genetic alterations have potentially a defective DNA damage repair mechanism/signaling pathway. When looking downstream of such pathway, *ATM* and *TP53* genetic alterations affected 10% and 20% of the metastases, respectively (Fig. 6b). In addition, and as observed above, a step-wise increment in the frequency of homozygous deletions affecting *CDKN2A* was observed from primary tumors to the metastases. Neoplasms with such alterations have a defective cell cycle pathway (Fig. 6b).

## Discussion

Despite improvements in the molecular profiling of PanNETs over the last decade, predicting the clinical behavior of these tumors and their response to specific therapies remains challenging. Such difficulties are mainly due to the high degree of heterogeneity observed in these neoplasms. With this in mind, we sought to meticulously characterize the genetic repertoire of primary PanNETs and metastases^10–12^ and unravel clinically informative alterations affecting these tumors. When looking at the molecular features of 87 primary PanNETs and 105 unmatched metastases, we noticed a significantly higher frequency of *CDKN2A* homozygous deletions in the metastases when compared to primary tumors, as well as poor survival outcomes in patients harboring these deletions. In addition, tumor evolution analysis of 6 primary tumors with matched metastases revealed that 4 metastases harbored private genetic alterations affecting *CDKN2A*. Previous studies have also observed an association of *CDKN2A* homozygous deletions with metastases from PanNETs^9,14^. Guo et al. also observed these deletions occurring exclusively in the metastases (4/10 patients) compared to matched primary tumors^9^. Beyond CN alterations, other *CDKN2A* inactivating mechanisms were also linked with survival outcomes in PanNETs, or with the risk of developing the disease. For instance, *CDKN2A* promoter hypermethylation has been associated with poor prognosis in pancreatic cancer^15^, and more specifically, in pancreatic endocrine neoplasms^16^. Pathogenic germline variants affecting *CDKN2A* have also been shown to be associated with the risk of developing such disease^17^. All of these observations, combined with the clinical outcome obtained in the present study, suggests that *CDKN2A* should be regarded as a diagnostic and prognostic biomarker in PanNETs. Applying genotyping analysis of this gene in clinical practice could greatly improve patient risk stratification.

Akin to patients harboring *CDKN2A* homozygous deletions, those harboring *TSC2* genetic alterations were also found to have poor survival outcomes, and a subset of metastases was found to harbor private *TSC2* somatic mutations coupled with LOH when compared to their matched primary tumors. Loss of TSC2 by immunohistochemistry in PanNETs was previously demonstrated to be associated with significantly shorter overall survival^18^. In the study from Guo et al., 3 out of 10 metastases were found to harbor private *TSC2* somatic mutations when compared with their matched primary tumors^9^. While *CDKN2A* belongs to the cell cycle signaling pathway, *TSC2* is a negative regulator of the mTOR signaling pathway. Hence, although patients with advanced PanNETs showed relatively high response rates when treated with capecitabine and temozolomide^19^, it would be interesting to determine if mTOR pathway inhibitors such as everolimus and its analog sirolimus could be promising drugs against PanNETs genotyped for the somatic loss of *TSC2*.

We also determined the CN alterations present in the 192 PanNETs and noticed that biallelic losses of *DAXX* or *ATRX* were associated with high frequencies of the FGA in both primary tumors and metastases. Such associations were previously described in PanNETs: Marinoni et al. have demonstrated that immunohistochemical loss of DAXX and ATRX was associated with chromosomal instability in 39 out of 142 PanNETs^20^; Scarpa et al. have also shown that biallelic inactivation of *DAXX* or *ATRX* were strongly associated with increased telomere length in 26 out of 86 PanNETs^6^. Because both tumor suppressor genes, *DAXX* and *ATRX*, regulate p53 chromatin binding and DNA damage response^21^, complete loss of one of these two genes would inactivate/disrupt the DNA damage repair signaling pathway and consequently compromise the genomic stability of PanNETs. Loss of *ATRX* or *DAXX* were reported as an independent prognostic factor for poor disease-free survival in these tumors^22^. Nonetheless, *ATRX/DAXX* mutations remain a topic of debate as prognostic markers. In fact, there have been conflicting studies, raising the possibility that *ATRX/DAXX* mutations may be associated with poor overall survival in low stage tumors, but with better survival outcomes in advanced stage or metastatic tumors^23^. Our findings suggest a clear and better survival outcome in metastatic patients harboring *DAXX* mutations, and no association between survival outcomes and *ATRX* genetic alterations in advanced PanNETs.

With the notion that a subset of PanNETs displays high levels of HRD, we also looked at the mutational signatures previously inferred in 33 primary PanNETs subjected to WGS from PCAWG^13^. We found that five tumors had a dominant signature 3 (“HRD-like”) associated with HRD features. When comparing the genetic repertoire of these tumors with those harboring a second dominant signature 3 (“HRD-ambiguous”) or with those with no evidence of signature 3 (“non-HRD”), we also found that pathogenic genetic alterations affecting *ATRX* and *DAXX* were enriched in the “HRD-like” group. An elegant study from Juhász et al. demonstrated that *ATRX* depletion in HeLa cells abolishes DNA repair synthesis and prevents the formation of sister chromatid exchanges at exogenously induced DNA double-stranded breaks (DSBs)^24^. The authors also described that *ATRX* partners with *DAXX* to incorporate histone H3.3 during homologous recombination-mediated repair of such exogenously induced DSBs^24^. Such observations definitely warrants additional studies regarding the association between HRD features and pathogenic mutations affecting *ATRX* and *DAXX* in PanNETs. To further investigate the function of *ATRX* in the DNA damage repair signaling pathway, Garbarino et al. created isogenic wild-type and *ATRX* knockout glioma cell lines using CRISPR-based gene targeting. This study revealed that loss of *ATRX* confers sensitivity to poly(ADP)- ribose polymerase (PARP) inhibitors, which was linked to an increase in replication stress^25^. Investigating whether PARP inhibitors could also be effective in *ATRX-* or *DAXX*-depleted PanNETs would be of great clinical interest to the affected patients.

Our study has significant limitations. As we could only retrieve the molecular and clinical features from pan-cancer studies, we could not assess the telomere lengths in the MSK-IMPACT-derived cohort, nor could we perform clinical associations according to tumor grade. The results from the tumor evolution analysis were limited by the reduced number of matched PanNETs included, and restricted by the use of in silico bioinformatics tools only. Finally, the mutational re-analyses performed in the metastatic cohort were restricted to cancer-related genes only, and we cannot rule out that other genetic alterations could play a role in these tumors studied here.

In conclusion, and considering the extremely high levels of heterogeneity in PanNETs, we highlighted relevant clinically informative alterations in advanced PanNETs, especially those affecting *CDKN2A*, *TSC2* (both conferring poor survival outcomes), *ATRX* and *DAXX* (both inducing chromosomal instability/homologous recombination deficiencies), that may be explored therapeutically or included in a daily routine for stratification of patients with a poorer prognosis. This knowledge may contribute to the development of targeted therapies tailored to the unique molecular vulnerabilities of these tumors. Understanding the molecular landscape of PanNETs may improve patient stratification for personalized treatments, potentially increasing efficacy while minimizing side effects, leading to innovative diagnostic tools, prognostic markers, and transforming the management and prognosis of this complex disease.

## Methods

### Case selection

All patients with PanNETs under an institutional review board–approved protocol at Memorial Sloan Kettering Cancer Center and whose tumors were subjected to targeted massively parallel sequencing via Memorial Sloan Kettering–Integrated Mutation Profiling of Actionable Cancer Targets (MSK-IMPACT)^10^ from 2014 to 2020 were identified (*n* = 211 cases sequenced) and accessed through cBioPortal. Cases not harboring any genetic alteration were excluded from the analysis (*n* = 19), leaving 192 PanNETs selected (Supplementary Table S1). Clinicopathological data, including age at diagnosis, sample type (87 primary tumors and 105 unmatched metastases), location and number of the metastases, and past cancer history, were also retrieved^10^ (Supplementary Table S1). Of the 87 primary PanNETs, 70 patients had evidence of metastases. In addition, 6 patients with primary PanNETs and matched metastases whose tumors were also subjected to targeted massively parallel sequencing via MSK-IMPACT were identified^11,12^. Besides this, 33 additional primary PanNETs subjected to WGS were identified and retrieved from the ICGC/TCGA Pan-Cancer Analysis of Whole Genomes (PCAWG) Consortium^13^.

### Massively Parallel Sequencing Analysis

Tumor and matched normal DNA samples were subjected to targeted massively parallel sequencing using MSK-IMPACT, which targets all exons and selected introns of 341-468 cancer genes^26,27^. Relevant genomic data derived from MSK-IMPACT included somatic mutations, structural variants, copy number (CN) alterations (gains and losses, gene amplifications, and homozygous deletions), TMB, and FGA^10,11^. The CN segment files were also retrieved to determine whether genes harboring somatic mutations were targeted by LOH^28^. CN alterations were estimated by taking the median of the log ratios per chromosomal arm. The absolute CN values were then calculated according to the tumor purity of a given sample, as previously described^28^. The cancer cell fractions (CCFs) of all somatic mutations were computed using ABSOLUTE^29^, taking into account the variant allele frequency (VAF) and the ploidy status of each somatic variant. Solutions from ABSOLUTE were manually reviewed. A mutation was classified as clonal if its probability of being clonal was >50% or if the lower bound of the 95% confidence interval of its CCF was >90%, as previously described^30^. Mutations that did not meet the above criteria were considered subclonal. Relevant genomic data from PCAWG included somatic mutations, structural variants, CN alterations, TMB, and mutational signatures^13^. CN alterations were inferred based on the median of the absolute CN values of a given gene, clustered by chromosomal arm. The probability of the ALT mechanism previously calculated for all the 33 PCAWG samples was also retrieved^31^.

### Genetic comparisons between primary and metastatic pancreatic neuroendocrine tumors, and with low and high fraction of the genome altered

The frequencies of somatic genetic alterations of 87 primary PanNETs were compared with those of 105 unmatched metastases (Supplementary Table S1). In addition, the frequencies of these alterations were also compared between the number of distant metastases each patient harbored. We used a cutoff of 2 distant metastases to stratify these patients for increased statistical power. We have also compared the frequencies of somatic genetic alterations between PanNETs with low and high FGA. For this, we used the median of the FGA as a cutoff (primary tumors: 37.3%; metastases: 47.7%) to stratify the samples into low and high FGA. All of these genetic comparisons (including the TMB), were performed using MSK-IMPACT-derived samples only (*n* = 192). The sole exception regards the mutation comparisons between primary tumors with evidence of metastases and primary tumors with no evidence of metastases. For these, we also added the mutation information of the 98 PanNETs retrieved from Scarpa et al.^6^. In that way, we ended up accruing 97 primary tumors with no evidence of metastases (17 primary tumors subjected to MSK-IMPACT and 80 primary tumors subjected to WGS (Scarpa et al.^6^)), and 88 primary tumors with evidence of metastases (70 primary tumors subjected to MSK-IMPACT and 18 primary tumors subjected to WGS (Scarpa et al.^6^)). For all of the genetic comparisons, only the 341 genes targeted by the smallest MSK-IMPACT panel were used.

### Tumor evolution analysis of primary pancreatic neuroendocrine tumors with matched metastasis

The clonal composition of each somatic mutation was determined using ABSOLUTE^29^, as described above. A given genetic alteration (somatic mutations and homozygous deletions) was considered “shared” if it was present in both the primary and matched metastatic lesion. We defined alterations “private to the primary lesion” and “private to the metastatic lesion” as those present only in the primary tumor or in the metastasis, respectively. Mutational signatures were defined by deconstructSigs using all single-nucleotide variants (SNVs)^32^ at default parameters, as previously described^33^, for samples with ≥10 somatic SNVs. To reconstruct the phylogeny of the primary and matched metastatic PanNETs, we used Treeomics^34^ based on all genetic alterations identified and the CCF values calculated above, as previously described^35,36^. For the construction of phylogenetic trees based on CN alterations, major and minor CNs were modeled through transducer-based pairwise comparison functions using MEDICC^37^, assuming a diploid state with no CN alterations to root the phylogenies.

### Genetic comparisons between pancreatic neuroendocrine tumors according to the presence or absence of homologous recombination deficiency features

For each of the 33 PanNETs subjected to WGS, we retrieved the frequency of the mutational signatures from the PCAWG study^13^. Each sample was stratified according to the frequency of the mutational signature associated with homologous recombination deficiency (HRD, Signature 3)^38^. PanNETs with a dominant signature 3 were considered “HRD-like” tumors (*n* = 5), those with a second dominant signature 3 were considered “HRD-ambiguous” tumors (*n* = 12), and those with no evidence of signature 3 were considered “non-HRD” tumors (*n* = 16). The frequencies of somatic genetic alterations were compared between these three groups.

### Pathway analyses

A DAVID pathway analysis was conducted based on genes affected by somatic genetic alterations^39^. Pathways found to be statistically significantly enriched (*p* < 0.01) in PanNETs and previously curated and reported in Sanchez-Vega et al. were selected^40^. Additionally, a mutual exclusivity analysis was performed using combinations of mutually exclusive alterations (CoMET) with a pairwise Fisher’s exact test^41^.

### Statistical analysis

Statistical analyses were performed using R v3.1.2. Fisher’s exact tests were employed for comparisons between categorical variables, whereas Mann–Whitney U tests were used for continuous variables. All tests were two-sided, and a *p*-value < 0.05 was considered statistically significant. Survival analyses were performed using univariate Cox regressions, and Kaplan–Meier curves were displayed using the R package survival^42^. For the survival analyses comprising the most affected genes in PanNETs, only the follow-up time from metastatic patients (*n* = 175) was considered. For a proper visualization, only recurrently altered genes per sample type are represented in the heatmaps.

## Supplementary information

Supplementary information

## Data Availability

All clinical and molecular data was retrieved from the cBioPortal database ([https://www.cbioportal.org/)](https://www.cbioportal.org/)).
