# Supplementary material for: CDKN2A homozygous deletions and TSC2 somatic mutations in metastatic pancreatic neuroendocrine tumors
Source: NPJ Precis Oncol. 2025 Dec 5;10:7. doi: 10.1038/s41698-025-01210-2 (PMC12775397; doi:10.1038/s41698-025-01210-2)
Supplement: Supplementary file 1 — Supplementary information [file 41698_2025_1210_MOESM1_ESM.pdf]

Supplementary Figure S1

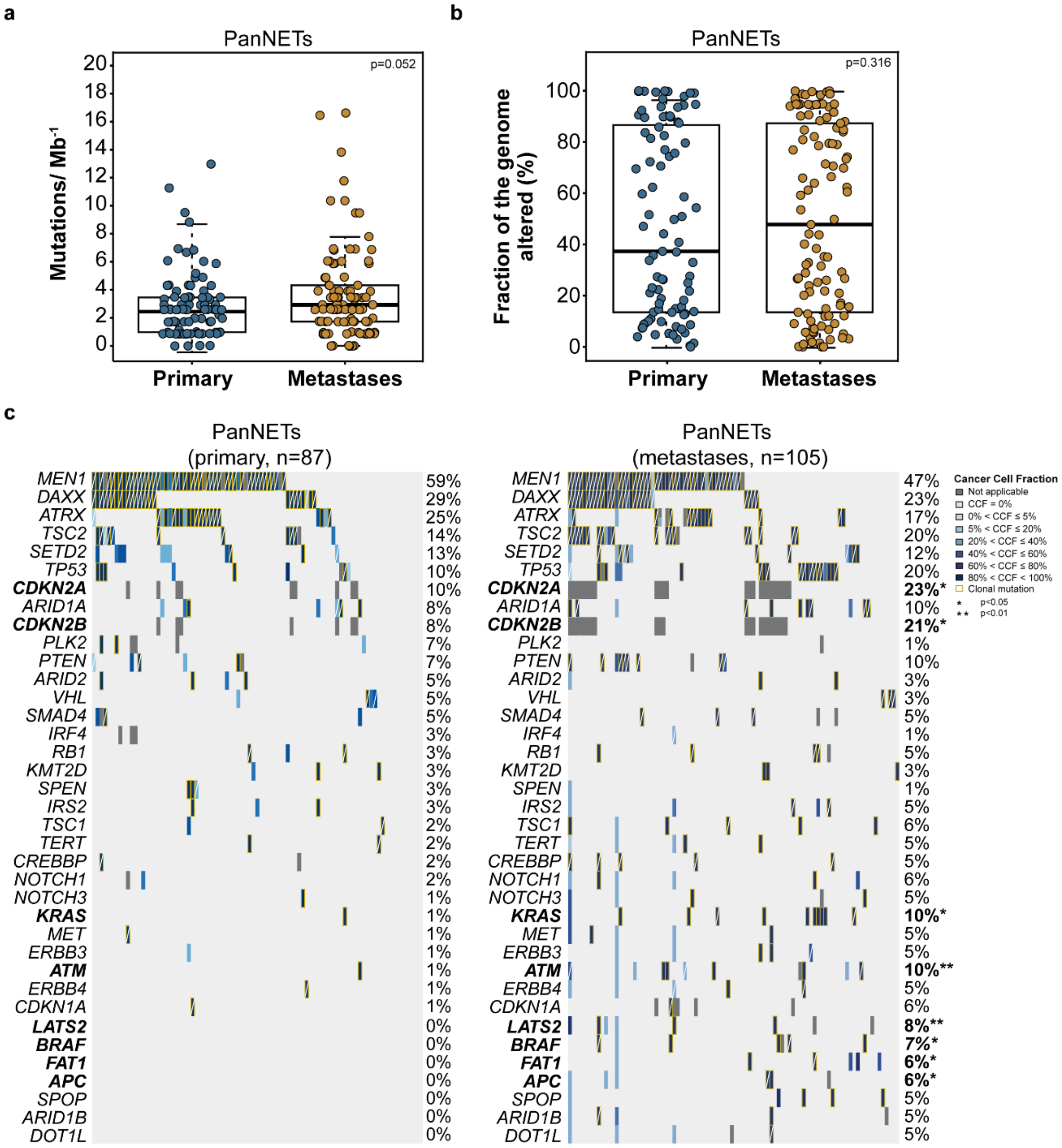

Supplementary figure 1: Genomic features of primary neoplasms and metastases from pancreatic neuroendocrine tumors and cancer cell fractions of somatic mutations present in these tumors. Comparisons between primary neoplasms and metastases from pancreatic neuroendocrine tumors (PanNETs) for (**a**), tumor mutational burden and (**b**), fraction of the genome altered. Heatmaps depicting cancer cell fractions of somatic mutations in (**c**), PanNETs, identified by MSK-IMPACT. Cancer cell fractions are color coded according to the legend. Statistical significance was evaluated in (**a**) and in (**b**), using the Mann-Whitney U test. CCF, cancer cell fraction; PanNETs, pancreatic neuroendocrine tumors.

Supplementary Figure S2

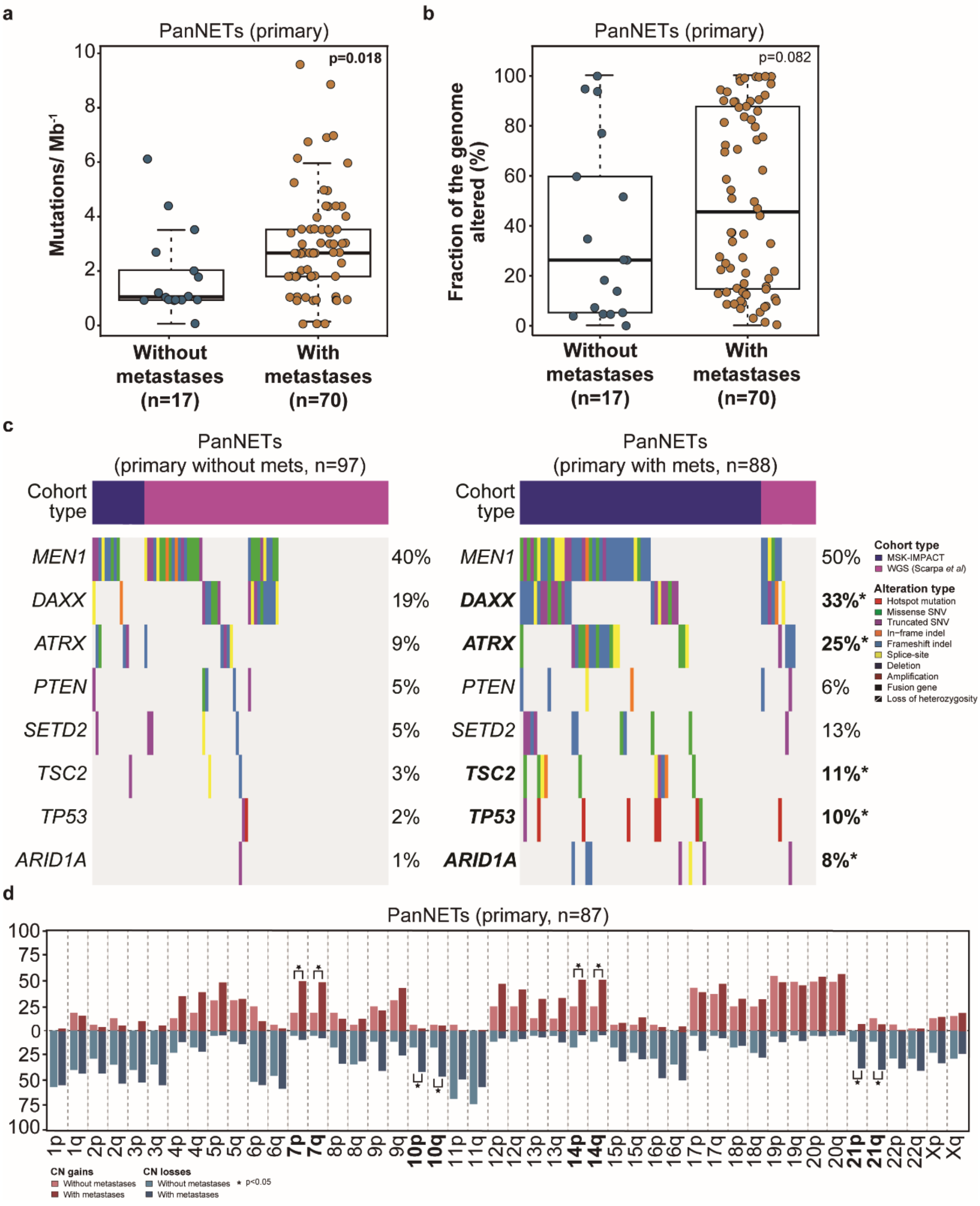

Supplementary figure 2: **Genomic features of primary pancreatic neuroendocrine tumors.** Comparisons between primary pancreatic neuroendocrine tumors (PanNETs) without evidence of metastasis and primary PanNETs with metastasis for (**a**), tumor mutational burden, (**b**), fraction of the genome altered, (**c**), recurrent somatic alterations from both the MSK-IMPACT and Scarpa datasets, and (**d**), copy number gains and losses. Alteration types are color-coded according to the legend. Statistical significance was evaluated in (**a**) and (**b**) using Mann-Whitney U test and in (**c**) and (**d**) using Fisher's exact test. CN, copy number; PanNETs, pancreatic neuroendocrine tumors.

Supplementary Figure S3

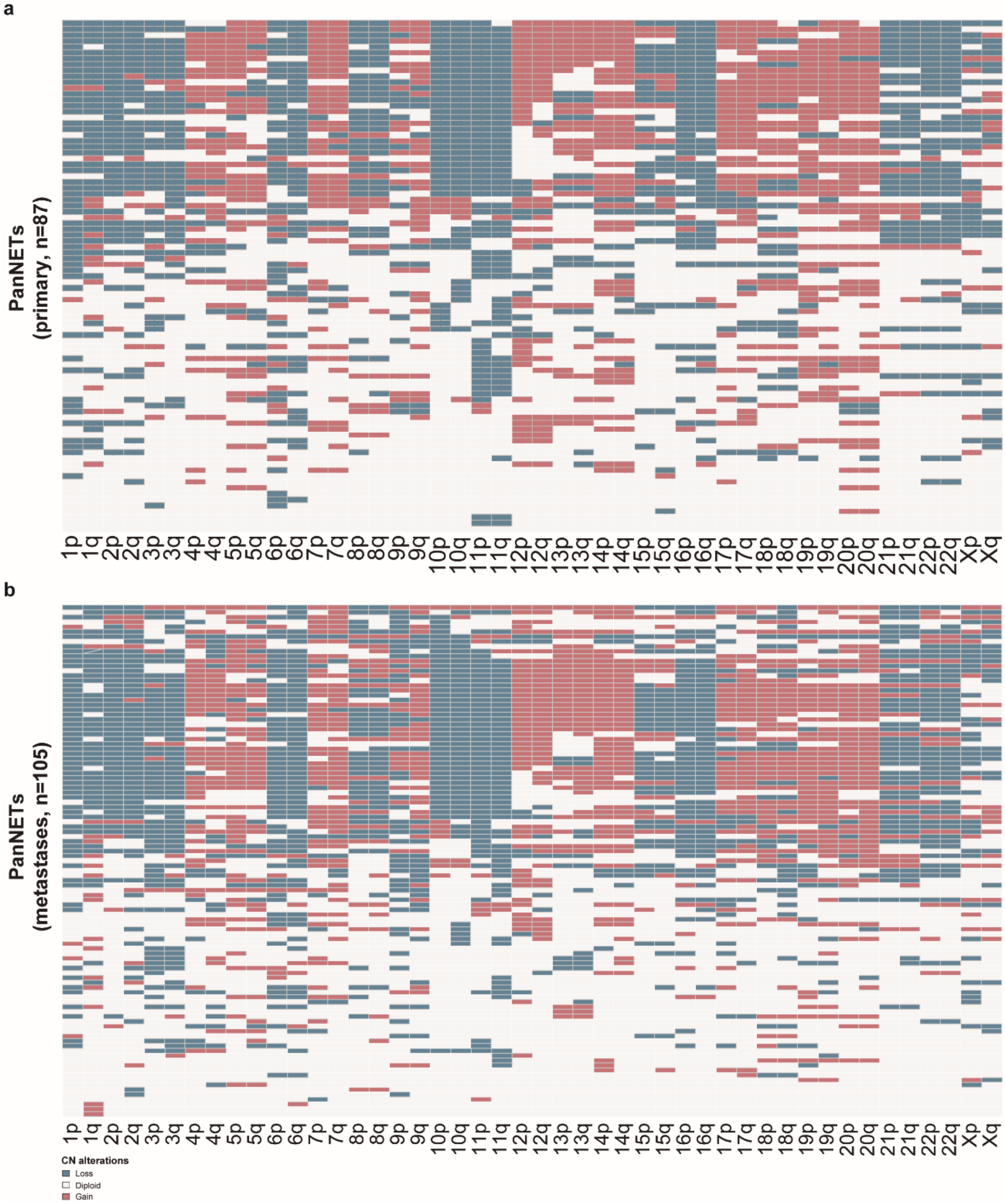

Supplementary figure 3: Copy number gains and losses of primary neoplasms and metastases from pancreatic neuroendocrine tumors. Heatmap depicting copy number alterations (gains and losses) affecting primary neoplams and metastases from pancreatic neuroendocrine tumors. Copy number alterations are color-coded according to the legend. CN, copy number; PanNETs, pancreatic neuroendocrine tumors.

Supplementary Figure S4

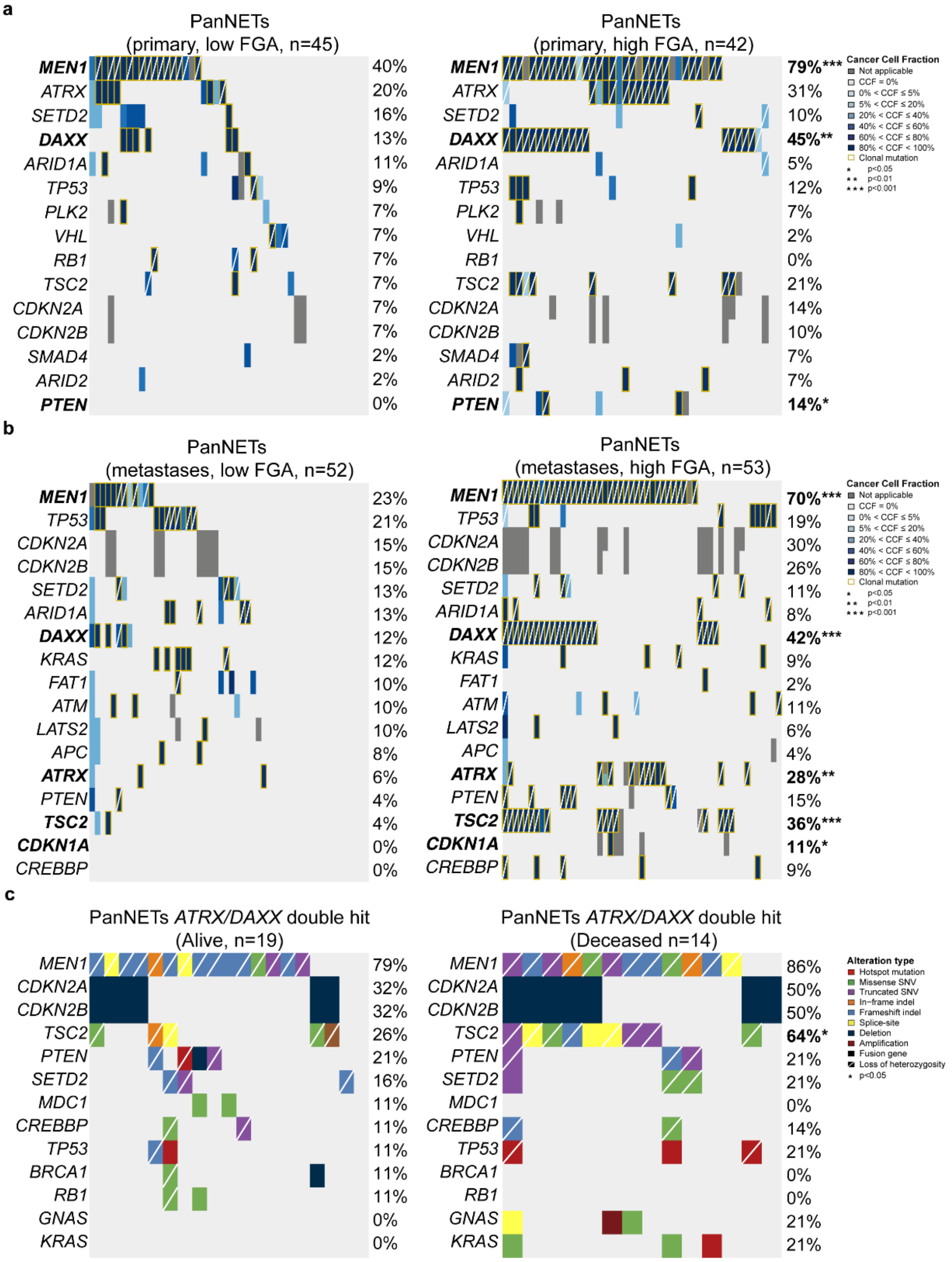

Supplementary figure 4: Cancer cell fractions of somatic mutations present in primary neoplasms and metastases from pancreatic neuroendocrine tumors with low and high fraction of the genome altered, and genomic features of PanNETs harboring *ATRX/DAXX* double genetic alterations. Heatmaps depicting cancer cell fractions of somatic mutations in primary pancreatic neuroendocrine tumors (PanNETs) (**a**) and in metastases from PanNETs (**b**) with low and high fraction of the genome altered identified by MSK-IMPACT. Comparisons between PanNETs harboring *ATRX/DAXX* double genetic alterations stratified by survival status for (**c**), recurrent somatic alterations. Cancer cell fractions are color coded according to the legend. CCF, cancer cell fraction; FGA, fraction of the genome altered; PanNETs, pancreatic neuroendocrine tumors.

Supplementary Figure S5

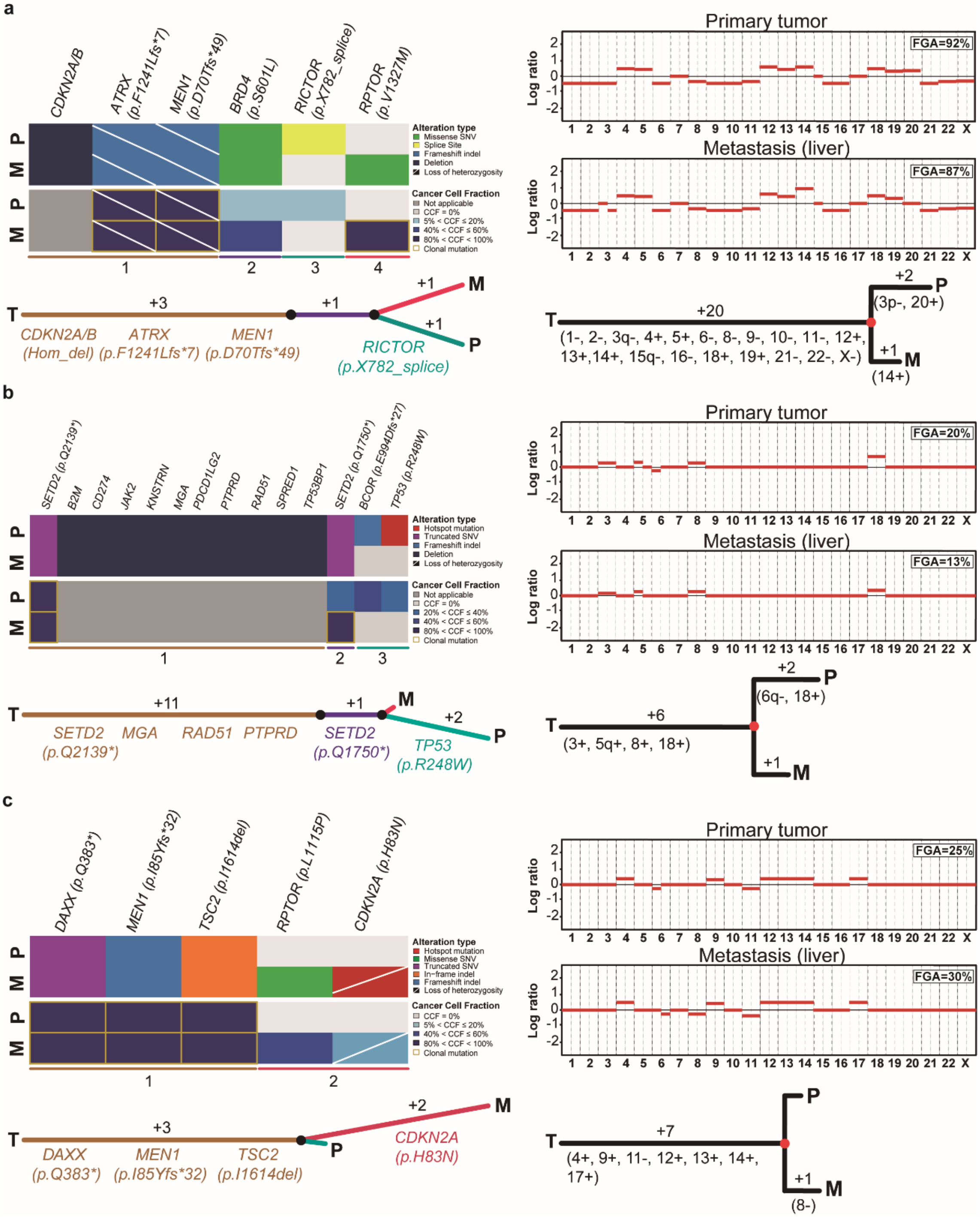

Supplementary figure 5: Clonal composition and phylogenetic analysis of primary pancreatic neuroendocrine tumors and matched metastases. Clonal composition and phylogenetic analysis of three cases (**a-c**). Heatmaps depicting the frequency of genetic alterations and cancer cell fractions of each somatic mutation in the primary pancreatic neuroendocrine tumors (PanNETs) and matched metastasis of a given case are shown (**top left**). The alteration types and cancer cell fraction are color-coded according to the legend. Copy number plots depicting segmented Log2 ratios (y-axis) according to genomic position (x-axis) of primary PanNETs and matched metastases are depicted (**top right**). Phylogenetic trees of primary PanNETs and matched metastases are shown **bottom left**). Trunk and branches are colored according to clusters, and the number of somatic mutations that result in the divergence of a clone/subclone from its

ancestor is shown. Pathogenic mutations that define a given clone are depicted. Phylogenetic trees based on copy number alterations are shown (**bottom right**). The numbers alongside the branches represent the number of copy number alterations. Gains and losses are shown in parentheses. M, metastasis; P, primary; T, trunk.

Table S1: Clinical features of primary and metastatic pancreatic neuroendocrine tumors subjected to targeted massively parallel sequencing.

**Features**	**Primary, n (%)**	**Metastases, n (%)**
**Age**		
Median	58	59
Range	26-86	28-82
**Histology**		
Pancreatic neuroendocrine	87 (45%)	105 (55%)
**Metastatic location**		
Liver	-	88 (84%)
Lymph node	-	8 (8%)
Biliary tract	-	2 (2%)
Bowel	-	2 (2%)
Adrenal gland	-	1 (1%)
Bone	-	1 (1%)
Intra-Abdominal	-	1 (1%)
Unspecified	-	2 (2%)
**Deceased status**		
Alive	73 (84%)	55 (52%)
Died of disease	14 (16%)	50 (48%)
**Overall survival**		
Median	30.9	17.1
Range	0.92-70.7	0.46-71.9

Table S2: Non-synonymous somatic mutations identified in pancreatic neuroendocrine tumors subjected to targeted massively parallel sequencing.

Sample ID	Cancer subtype	Sample type	Symbol	Aminoacid change	Hotspot	Chromosome	Position	Reference	Alternate	Mutation type	Tumor MAF	Tumor DP	LOH	Cancer cell fraction	Clonal status
P-0002369-T01-IM3	Pancreatic neuroendocrine tumor	Primary	PPP2R1A	p.R183Q	TRUE	19	52715983	G	A	Missense_Mutation	0,039390089	787	FALSE	0.13	Subclonal
P-0002369-T01-IM3	Pancreatic neuroendocrine tumor	Primary	MEN1	p.K120del	FALSE	11	64577222	CTT	-	In_Frame_Del	0,496240602	532	TRUE	1	Clonal
P-0002369-T01-IM3	Pancreatic neuroendocrine tumor	Primary	PTEN	p.X212_splice	FALSE	10	89717608	A	G	Splice_Site	0,093093093	333	FALSE	0.22	Subclonal
P-0002369-T01-IM3	Pancreatic neuroendocrine tumor	Primary	CARD11	p.A687T	FALSE	7	2962849	C	T	Missense_Mutation	0,254700855	585	FALSE	0.84	Clonal
P-0002369-T01-IM3	Pancreatic neuroendocrine tumor	Primary	ARID1A	p.K2159Pfs*64	FALSE	1	27106863	AAAGA	-	Frame_Shift_Del	0,240184758	433	FALSE	0.56	Subclonal
P-0002369-T01-IM3	Pancreatic neuroendocrine tumor	Primary	ATRX	p.E2265del	FALSE	X	76778784	CTC	-	In_Frame_Del	0,082369942	692	FALSE	0.20	Subclonal
P-0002619-T01-IM3	Pancreatic neuroendocrine tumor	Primary	ATRX	p.R1803H	FALSE	X	76874314	C	T	Missense_Mutation	0,262390671	343	FALSE	1	Clonal
P-0002619-T01-IM3	Pancreatic neuroendocrine tumor	Primary	CDH1	p.R48Tfs*5	FALSE	16	68772294	GAGGCCGCGT	-	Frame_Shift_Del	0,207792208	154	FALSE	1	Clonal
P-0002839-T01-IM3	Pancreatic neuroendocrine tumor	Primary	TP53	p.R248Q	TRUE	17	7577538	C	T	Missense_Mutation	0,199063232	854	FALSE	0.51	Subclonal
P-0002839-T01-IM3	Pancreatic neuroendocrine tumor	Primary	MEN1	p.Q513*	FALSE	11	64572117	G	A	Nonsense_Mutation	0,68338558	319	TRUE	1	Clonal
P-0002839-T01-IM3	Pancreatic neuroendocrine tumor	Primary	ATRX	p.A1272P	FALSE	X	76920263	C	G	Missense_Mutation	0,722426471	544	TRUE	1	Clonal
P-0004919-T01-IM5	Pancreatic neuroendocrine tumor	Primary	ATRX	p.T844Yfs*5	FALSE	X	76938218	-	A	Frame_Shift_Ins	0,081967213	305	FALSE	0.37	Subclonal
P-0006710-T01-IM5	Pancreatic neuroendocrine tumor	Primary	STAG2	p.R370Q	FALSE	X	123185062	G	A	Missense_Mutation	0,021153846	520	FALSE	0.10	Subclonal
P-0006710-T01-IM5	Pancreatic neuroendocrine tumor	Primary	ATRX	p.Q545*	FALSE	X	76939115	G	A	Nonsense_Mutation	0,116197183	284	FALSE	0.30	Subclonal
P-0006710-T01-IM5	Pancreatic neuroendocrine tumor	Primary	ARID1A	p.Q1650Afs*47	FALSE	1	27101662	CA	-	Frame_Shift_Del	0,149139579	523	FALSE	0.39	Subclonal
P-0006710-T01-IM5	Pancreatic neuroendocrine tumor	Primary	MEN1	p.I85Sfs*33	FALSE	11	64577330	AGAC	-	Frame_Shift_Del	0,169491525	826	FALSE	0.44	Subclonal
P-0006710-T01-IM5	Pancreatic neuroendocrine tumor	Primary	SETD2	p.G320Vfs*20	FALSE	3	47165172	A	-	Frame_Shift_Del	0,096463023	311	FALSE	0.25	Subclonal
P-0010090-T01-IM5	Pancreatic neuroendocrine tumor	Primary	MGA	p.S1778*	FALSE	15	42040955	C	G	Nonsense_Mutation	0,55726257	716	FALSE	0.97	Clonal
P-0010090-T01-IM5	Pancreatic neuroendocrine tumor	Primary	ATRX	p.K1725Mfs*10	FALSE	X	76875958	TTTT	-	Frame_Shift_Del	0,632404181	574	TRUE	1	Clonal
P-0010090-T01-IM5	Pancreatic neuroendocrine tumor	Primary	MEN1	p.D70Tfs*49	FALSE	11	64577375	G	-	Frame_Shift_Del	0,611615245	551	TRUE	1	Clonal
P-0018828-T01-IM6	Pancreatic neuroendocrine tumor	Primary	MEN1	p.P378R	FALSE	11	64573174	G	C	Missense_Mutation	0,324777887	1013	FALSE	1	Clonal
P-0018828-T01-IM6	Pancreatic neuroendocrine tumor	Primary	ATRX	p.D2136Y	FALSE	X	76814238	C	A	Missense_Mutation	0,283062645	862	FALSE	1	Clonal
P-0020967-T01-IM6	Pancreatic neuroendocrine tumor	Primary	MEN1	p.F164Lfs*26	FALSE	11	64575540	A	-	Frame_Shift_Del	0,294936709	790	TRUE	1	Clonal
P-0020967-T01-IM6	Pancreatic neuroendocrine tumor	Primary	HGF	p.V725L	FALSE	7	81331911	C	A	Missense_Mutation	0,085714286	315	FALSE	0.39	Subclonal
P-0020967-T01-IM6	Pancreatic neuroendocrine tumor	Primary	ATRX	p.E1448Kfs*42	FALSE	X	76907819	C	-	Frame_Shift_Del	0,433070866	127	FALSE	1	Clonal
P-0022327-T01-IM6	Pancreatic neuroendocrine tumor	Primary	ATRX	p.X1653_splice	FALSE	X	76888873	C	T	Splice_Site	0,858565737	502	TRUE	1	Clonal
P-0022652-T01-IM6	Pancreatic neuroendocrine tumor	Primary	MEN1	p.Q263*	FALSE	11	64575035	G	A	Nonsense_Mutation	0,411177645	501	TRUE	1	Clonal
P-0022652-T01-IM6	Pancreatic neuroendocrine tumor	Primary	ATRX	p.N486Ifs*28	FALSE	X	76939291	T	-	Frame_Shift_Del	0,334821429	448	TRUE	1	Clonal
P-0022868-T01-IM6	Pancreatic neuroendocrine tumor	Primary	MEN1	p.W225Gfs*4	FALSE	11	64575149	A	-	Frame_Shift_Del	0,911854103	329	TRUE	0.98	Clonal
P-0022868-T01-IM6	Pancreatic neuroendocrine tumor	Primary	PIK3R1	p.Y467C	FALSE	5	67589637	A	G	Missense_Mutation	0,255952381	168	FALSE	0.53	Subclonal
P-0022868-T01-IM6	Pancreatic neuroendocrine tumor	Primary	HIST1H3G	p.E51G	FALSE	6	26271461	T	C	Missense_Mutation	0,083557951	371	FALSE	0.09	Subclonal
P-0022868-T01-IM6	Pancreatic neuroendocrine tumor	Primary	ATRX	p.K242Nfs*15	FALSE	X	76940022	T	-	Frame_Shift_Del	0,933701657	181	TRUE	1	Clonal
P-0024338-T01-IM6	Pancreatic neuroendocrine tumor	Primary	PTEN	p.P248Tfs*5	FALSE	10	89717715	-	A	Frame_Shift_Ins	0,105590062	322	TRUE	0.19	Subclonal
P-0024338-T01-IM6	Pancreatic neuroendocrine tumor	Primary	MEN1	p.L34R	FALSE	11	64577481	A	C	Missense_Mutation	0,770588235	340	TRUE	1	Clonal
P-0024338-T01-IM6	Pancreatic neuroendocrine tumor	Primary	DAXX	p.R328G	FALSE	6	33288570	G	C	Missense_Mutation	0,19665272	478	TRUE	0.36	Subclonal
P-0024338-T01-IM6	Pancreatic neuroendocrine tumor	Primary	DAXX	p.R203Gfs*26	FALSE	6	33288945	G	-	Frame_Shift_Del	0,56402439	328	TRUE	1	Clonal
P-0024338-T01-IM6	Pancreatic neuroendocrine tumor	Primary	ATRX	p.I2175L	FALSE	X	76813098	T	G	Missense_Mutation	0,06557377	427	TRUE	0.15	Subclonal
P-0025493-T01-IM6	Pancreatic neuroendocrine tumor	Primary	MTOR	p.T1977R	TRUE	1	11188164	G	C	Missense_Mutation	0,220833333	480	FALSE	0.37	Subclonal
P-0025493-T01-IM6	Pancreatic neuroendocrine tumor	Primary	HLA-B	p.S121N	FALSE	6	31324201	C	T	Missense_Mutation	0,245614035	57	FALSE	0.41	Subclonal
P-0025493-T01-IM6	Pancreatic neuroendocrine tumor	Primary	MEN1	p.D185Cfs*14	FALSE	11	64575476	GATCC	-	Frame_Shift_Del	0,70777027	592	TRUE	1	Clonal
P-0025493-T01-IM6	Pancreatic neuroendocrine tumor	Primary	EP300	p.Q709R	FALSE	22	41542815	A	G	Missense_Mutation	0,235294118	306	FALSE	0.39	Subclonal
P-0025493-T01-IM6	Pancreatic neuroendocrine tumor	Primary	ATRX	p.N2140S	FALSE	X	76814225	T	C	Missense_Mutation	0,636363636	253	TRUE	1	Clonal
P-0025493-T01-IM6	Pancreatic neuroendocrine tumor	Primary	SPEN	p.P831*	FALSE	1	16255225	CCC	A	Frame_Shift_Del	0,22972973	370	TRUE	0.38	Subclonal
P-0027149-T01-IM6	Pancreatic neuroendocrine tumor	Primary	SPEN	p.K338*	FALSE	1	16235946	A	T	Nonsense_Mutation	0,253799392	658	FALSE	0.91	Clonal
P-0027149-T01-IM6	Pancreatic neuroendocrine tumor	Primary	ARID1A	p.D1025Wfs*11	FALSE	1	27094365	GACCGTTATC	-	Frame_Shift_Del	0,282841823	746	FALSE	1	Clonal
P-0027149-T01-IM6	Pancreatic neuroendocrine tumor	Primary	CDKN2C	p.L24F	FALSE	1	51436112	G	T	Missense_Mutation	0,191176471	680	FALSE	0.69	Subclonal
P-0027149-T01-IM6	Pancreatic neuroendocrine tumor	Primary	MEN1	p.S432I	FALSE	11	64572576	C	A	Missense_Mutation	0,292525773	776	FALSE	1	Clonal
P-0027149-T01-IM6	Pancreatic neuroendocrine tumor	Primary	ERBB3	p.N101K	FALSE	12	56478847	C	A	Missense_Mutation	0,080519481	1155	FALSE	0.37	Subclonal
P-0027149-T01-IM6	Pancreatic neuroendocrine tumor	Primary	ERCC5	p.M314I	FALSE	13	103514441	G	T	Missense_Mutation	0,142091153	373	FALSE	0.51	Subclonal
P-0027149-T01-IM6	Pancreatic neuroendocrine tumor	Primary	IGF1R	p.K77N	FALSE	15	99250927	G	T	Missense_Mutation	0,265842349	647	FALSE	0.96	Clonal
P-0027149-T01-IM6	Pancreatic neuroendocrine tumor	Primary	ANKRD11	p.V388F	FALSE	16	89351788	C	A	Missense_Mutation	0,076530612	980	FALSE	0.28	Subclonal
P-0027149-T01-IM6	Pancreatic neuroendocrine tumor	Primary	ALOX12B	p.L37Q	FALSE	17	7990651	A	T	Missense_Mutation	0,224281742	1079	FALSE	0.81	Subclonal
P-0027149-T01-IM6	Pancreatic neuroendocrine tumor	Primary	TMPRSS2	p.G362E	FALSE	21	42843834	C	T	Missense_Mutation	0,108225108	693	FALSE	0.39	Subclonal
P-0027149-T01-IM6	Pancreatic neuroendocrine tumor	Primary	PREX2	p.T289S	FALSE	8	68956747	A	T	Missense_Mutation	0,208641975	810	FALSE	0.75	Subclonal
P-0027149-T01-IM6	Pancreatic neuroendocrine tumor	Primary	TSC1	p.G147V	FALSE	9	135798803	C	A	Missense_Mutation	0,169902913	618	FALSE	0.61	Subclonal
P-0027149-T01-IM6	Pancreatic neuroendocrine tumor	Primary	ATRX	p.R1425Gfs*65	FALSE	X	76909636	C	-	Frame_Shift_Del	0,301282051	312	TRUE	1	Clonal
P-0027149-T01-IM6	Pancreatic neuroendocrine tumor	Primary	SPEN	p.K338*	FALSE	1	16235946	A	T	Nonsense_Mutation	0,253799392	658	FALSE	0.91	Clonal
P-0027149-T01-IM6	Pancreatic neuroendocrine tumor	Primary	ARID1A	p.D1025Wfs*11	FALSE	1	27094365	GACCGTTATC	-	Frame_Shift_Del	0,282841823	746	FALSE	1	Clonal
P-0027149-T01-IM6	Pancreatic neuroendocrine tumor	Primary	CDKN2C	p.L24F	FALSE	1	51436112	G	T	Missense_Mutation	0,191176471	680	FALSE	0.69	Subclonal
P-0027149-T01-IM6	Pancreatic neuroendocrine tumor	Primary	MEN1	p.S432I	FALSE	11	64572576	C	A	Missense_Mutation	0,292525773	776	FALSE	1	Clonal
P-0027149-T01-IM6	Pancreatic neuroendocrine tumor	Primary	ERBB3	p.N101K	FALSE	12	56478847	C	A	Missense_Mutation	0,080519481	1155	FALSE	0.37	Subclonal
P-0027149-T01-IM6	Pancreatic neuroendocrine tumor	Primary	ERCC5	p.M314I	FALSE	13	103514441	G	T	Missense_Mutation	0,142091153	373	FALSE	0.51	Subclonal
P-0027149-T01-IM6	Pancreatic neuroendocrine tumor	Primary	IGF1R	p.K77N	FALSE	15	99250927	G	T	Missense_Mutation	0,265842349	647	FALSE	0.96	Clonal
P-0027149-T01-IM6	Pancreatic neuroendocrine tumor	Primary	ANKRD11	p.V388F	FALSE	16	89351788	C	A	Missense_Mutation	0,076530612	980	FALSE	0.28	Subclonal
P-0027149-T01-IM6	Pancreatic neuroendocrine tumor	Primary	ALOX12B	p.L37Q	FALSE	17	7990651	A	T	Missense_Mutation	0,224281742	1079	FALSE	0.81	Subclonal
P-0027149-T01-IM6	Pancreatic neuroendocrine tumor	Primary	TMPRSS2	p.G362E	FALSE	21	42843834	C	T	Missense_Mutation	0,108225108	693	FALSE	0.39	Subclonal
P-0027149-T01-IM6	Pancreatic neuroendocrine tumor	Primary	PREX2	p.T289S	FALSE	8	68956747	A	T	Missense_Mutation	0,208641975	810	FALSE	0.75	Subclonal
P-0027149-T01-IM6	Pancreatic neuroendocrine tumor	Primary	TSC1	p.G147V	FALSE	9	135798803	C	A	Missense_Mutation	0,169902913	618	FALSE	0.61	Subclonal
P-0027194-T01-IM6	Pancreatic neuroendocrine tumor	Primary	MEN1	p.E222Afs*14	FALSE	11	64575366	CT	-	Frame_Shift_Del	0,635402906	757	TRUE	1	Clonal
P-0027194-T01-IM6	Pancreatic neuroendocrine tumor	Primary	TSC2	p.N1214Y	FALSE	16	2131625	A	T	Missense_Mutation	0,540983607	793	TRUE	1	Clonal
P-0027194-T01-IM6	Pancreatic neuroendocrine tumor	Primary	ATRX	p.Y1758H	FALSE	X	76875863	A	G	Missense_Mutation	0,75	172	FALSE	1	Clonal
P-0032031-T01-IM6	Pancreatic neuroendocrine tumor	Primary	HLA-A	p.R30K	FALSE	6	29910549	G	A	Missense_Mutation	0,097959184	245	FALSE	0.59	Subclonal
P-0032031-T01-IM6	Pancreatic neuroendocrine tumor	Primary	ATRX	p.E1455*	FALSE	X	76907798	C	A	Nonsense_Mutation	0,313479624	319	TRUE	1	Clonal
P-0033739-T01-IM6	Pancreatic neuroendocrine tumor	Primary	ARID1A	p.E1802*	FALSE	1	27105793	G	T	Nonsense_Mutation	0,051282051	273	FALSE	0.43	Subclonal
P-0033739-T01-IM6	Pancreatic neuroendocrine tumor	Primary	IRS2	p.S262W	FALSE	13	110437616	G	C	Missense_Mutation	0,138801262	317	FALSE	1	Clonal
P-0033739-T01-IM6	Pancreatic neuroendocrine tumor	Primary	RTEL1	p.T44M	FALSE	20	62292679	C	T	Missense_Mutation	0,09057971	276	FALSE	0.75	Subclonal
P-0033739-T01-IM6	Pancreatic neuroendocrine tumor	Primary	PGR	p.E843D	FALSE	11	100912793	C	G	Missense_Mutation	0,089171975	314	FALSE	0.74	Subclonal
P-0033739-T01-IM6	Pancreatic neuroendocrine tumor	Primary	KDM5A	p.R264Q	FALSE	12	464403	C	T	Missense_Mutation	0,093023256	387	FALSE	0.87	Clonal
P-0033739-T01-IM6	Pancreatic neuroendocrine tumor	Primary	KMT2D	p.S1043C	FALSE	12	49444243	G	C	Missense_Mutation	0,116033755	474	FALSE	0.97	Clonal

Table S2

Sample ID	Cancer subtype	Sample type	Symbol	Aminoacid change	Hotspot	Chromosome	Position	Reference	Alternate	Mutation type	Tumor MAF	Tumor DP	LOH	Cancer cell fraction	Clonal status
P-0033739-T01-IM6	Pancreatic neuroendocrine tumor	Primary	HNF1A	p.E552Q	FALSE	12	121437316	G	C	Missense_Mutation	0,133858268	381	FALSE	1	Clonal
P-0033739-T01-IM6	Pancreatic neuroendocrine tumor	Primary	CDH1	p.F189L	FALSE	16	68842631	C	G	Missense_Mutation	0,082840237	338	FALSE	0.61	Subclonal
P-0033739-T01-IM6	Pancreatic neuroendocrine tumor	Primary	STAT5B	p.E438Q	FALSE	17	40369246	C	G	Missense_Mutation	0,090464548	409	FALSE	0.75	Subclonal
P-0033739-T01-IM6	Pancreatic neuroendocrine tumor	Primary	STAT5B	p.E335Q	FALSE	17	40370335	C	G	Missense_Mutation	0,056886228	334	FALSE	0.47	Subclonal
P-0033739-T01-IM6	Pancreatic neuroendocrine tumor	Primary	CARM1	p.D190H	FALSE	19	11022869	G	C	Missense_Mutation	0,116666667	360	FALSE	0.86	Clonal
P-0033739-T01-IM6	Pancreatic neuroendocrine tumor	Primary	KMT2B	p.F2590L	FALSE	19	36228990	C	G	Missense_Mutation	0,123076923	455	FALSE	1	Clonal
P-0033739-T01-IM6	Pancreatic neuroendocrine tumor	Primary	WHSC1L1	p.E1113Q	FALSE	8	38146169	C	G	Missense_Mutation	0,082533589	521	FALSE	0.85	Clonal
P-0033739-T01-IM6	Pancreatic neuroendocrine tumor	Primary	AMER1	p.P49L	FALSE	X	63413021	G	A	Missense_Mutation	0,107238606	373	FALSE	0.80	Subclonal
P-0033739-T01-IM6	Pancreatic neuroendocrine tumor	Primary	ATRX	p.S1331F	FALSE	X	76918999	G	A	Missense_Mutation	0,07278481	316	FALSE	0.60	Subclonal
P-0034628-T01-IM6	Pancreatic neuroendocrine tumor	Primary	ATRX	p.X1900_splice	FALSE	X	76855290	C	T	Splice_Site	0,568627451	204	FALSE	1	Clonal
P-0034628-T01-IM6	Pancreatic neuroendocrine tumor	Primary	MEN1	p.M283Wfs*35	FALSE	11	64574544	GCCAGGTTCCCTAAGGCCATG	CCCAGGCCAT	Frame_Shift_Del	0,539393939	825	TRUE	1	Clonal
P-0034628-T01-IM6	Pancreatic neuroendocrine tumor	Primary	SPRED1	p.S290*	FALSE	15	38643399	C	G	Nonsense_Mutation	0,28	675	FALSE	1	Clonal
P-0034628-T01-IM6	Pancreatic neuroendocrine tumor	Primary	MEN1	p.M283Wfs*3	FALSE	11	64574564	G	-	Frame_Shift_Del	0,537212449	739	TRUE	1	Clonal
P-0037352-T01-IM6	Pancreatic neuroendocrine tumor	Primary	MEN1	p.P498Rfs*66	FALSE	11	64572161	G	-	Frame_Shift_Del	0,647798742	318	TRUE	1	Clonal
P-0037352-T01-IM6	Pancreatic neuroendocrine tumor	Primary	KDM5C	p.A928V	FALSE	X	53226066	G	A	Missense_Mutation	0,543157895	475	FALSE	1	Clonal
P-0037352-T01-IM6	Pancreatic neuroendocrine tumor	Primary	PTPRS	p.R1829W	FALSE	19	5210482	G	A	Missense_Mutation	0,415584416	385	FALSE	1	Clonal
P-0037352-T01-IM6	Pancreatic neuroendocrine tumor	Primary	KEAP1	p.S275L	FALSE	19	10602754	G	A	Missense_Mutation	0,465944272	646	FALSE	1	Clonal
P-0037352-T01-IM6	Pancreatic neuroendocrine tumor	Primary	RET	p.E621K	FALSE	10	43609105	G	A	Missense_Mutation	0,345565749	327	FALSE	1	Clonal
P-0037352-T01-IM6	Pancreatic neuroendocrine tumor	Primary	SPEN	p.A2105Lfs*33	FALSE	1	16259043	G	-	Frame_Shift_Del	0,205583756	394	FALSE	1	Clonal
P-0037352-T01-IM6	Pancreatic neuroendocrine tumor	Primary	PAX5	p.P32S	FALSE	9	37020751	G	A	Missense_Mutation	0,257217848	381	FALSE	1	Clonal
P-0037352-T01-IM6	Pancreatic neuroendocrine tumor	Primary	XPO1	p.A985T	FALSE	2	61709534	C	T	Missense_Mutation	0,616724739	287	FALSE	1	Clonal
P-0037352-T01-IM6	Pancreatic neuroendocrine tumor	Primary	CDKN1A	p.C34*	FALSE	6	36651978	TG	-	Frame_Shift_Del	0,698369565	368	TRUE	1	Clonal
P-0037352-T01-IM6	Pancreatic neuroendocrine tumor	Primary	GATA3	p.R305*	FALSE	10	8106093	C	T	Nonsense_Mutation	0,302325581	215	FALSE	1	Clonal
P-0037352-T01-IM6	Pancreatic neuroendocrine tumor	Primary	KMT2A	p.P3818S	FALSE	11	118391539	C	T	Missense_Mutation	0,705128205	234	FALSE	1	Clonal
P-0037352-T01-IM6	Pancreatic neuroendocrine tumor	Primary	PIK3C2G	p.N915Tfs*24	FALSE	12	18649065	A	-	Frame_Shift_Del	0,195652174	414	FALSE	0.87	Clonal
P-0037352-T01-IM6	Pancreatic neuroendocrine tumor	Primary	PIK3C2G	p.K1157E	FALSE	12	18715638	A	G	Missense_Mutation	0,247706422	218	FALSE	1	Clonal
P-0037352-T01-IM6	Pancreatic neuroendocrine tumor	Primary	ARID2	p.R1464H	FALSE	12	46246297	G	A	Missense_Mutation	0,391304348	437	FALSE	1	Clonal
P-0037352-T01-IM6	Pancreatic neuroendocrine tumor	Primary	IRS2	p.V830Wfs*36	FALSE	13	110435914	G	-	Frame_Shift_Del	0,230582524	412	FALSE	1	Clonal
P-0037352-T01-IM6	Pancreatic neuroendocrine tumor	Primary	ZFHX3	p.P2055L	FALSE	16	72830417	G	A	Missense_Mutation	0,1	150	FALSE	0.35	Subclonal
P-0037352-T01-IM6	Pancreatic neuroendocrine tumor	Primary	GNAS	p.Q195R	FALSE	20	57484270	A	G	Missense_Mutation	0,225543478	368	FALSE	1	Clonal
P-0037352-T01-IM6	Pancreatic neuroendocrine tumor	Primary	BAP1	p.A83T	FALSE	3	52442498	C	T	Missense_Mutation	0,23715415	253	FALSE	0.82	Clonal
P-0037352-T01-IM6	Pancreatic neuroendocrine tumor	Primary	EPHB1	p.R663W	FALSE	3	134911522	C	T	Missense_Mutation	0,616197183	284	FALSE	1	Clonal
P-0037352-T01-IM6	Pancreatic neuroendocrine tumor	Primary	TET2	p.H1727Y	FALSE	4	106196846	C	T	Missense_Mutation	0,463022508	311	FALSE	1	Clonal
P-0037352-T01-IM6	Pancreatic neuroendocrine tumor	Primary	CSF1R	p.A444D	FALSE	5	149449615	G	T	Missense_Mutation	0,159041394	459	FALSE	0.87	Clonal
P-0037352-T01-IM6	Pancreatic neuroendocrine tumor	Primary	FLT4	p.M880Cfs*2	FALSE	5	180046674	T	-	Frame_Shift_Del	0,190371991	457	FALSE	0.85	Clonal
P-0037352-T01-IM6	Pancreatic neuroendocrine tumor	Primary	EPHA7	p.V472A	FALSE	6	93982050	A	G	Missense_Mutation	0,286931818	352	FALSE	0.99	Clonal
P-0037352-T01-IM6	Pancreatic neuroendocrine tumor	Primary	JAK2	p.L510del	FALSE	9	5069936	CTT	-	In_Frame_Del	0,197802198	273	FALSE	0.88	Clonal
P-0037352-T01-IM6	Pancreatic neuroendocrine tumor	Primary	ATRX	p.X2233_splice	FALSE	X	76812920	A	G	Splice_Site	0,321192053	302	FALSE	1	Clonal
P-0046290-T01-IM6	Pancreatic neuroendocrine tumor	Primary	SETD2	p.S1624F	FALSE	3	47144882	G	A	Missense_Mutation	0,072625698	179	FALSE	0.27	Subclonal
P-0046290-T01-IM6	Pancreatic neuroendocrine tumor	Primary	SETD2	p.R1407Kfs*8	FALSE	3	47161906	-	T	Frame_Shift_Ins	0,090163934	122	FALSE	0.33	Subclonal
P-0046290-T01-IM6	Pancreatic neuroendocrine tumor	Primary	MEN1	p.L3Sfs*83	FALSE	11	64577477	CACCAGGTCCGGCTCCTCTCGGCCCAGCTCGGCAGCAAACAGGCGCACCA CGTCGTCGATGGAGCGCAGCGGGAACAGCGTCTTCTGGGCGGCCTTCAGC	-	Frame_Shift_Del	0,171597633	338	FALSE	1	Clonal
P-0046290-T01-IM6	Pancreatic neuroendocrine tumor	Primary	ATRX	p.S1390Mfs*100	FALSE	X	76912095	C	-	Frame_Shift_Del	0,376	125	FALSE	1	Clonal
P-0047499-T01-IM6	Pancreatic neuroendocrine tumor	Primary	MEN1	p.W126*	FALSE	11	64577205	C	T	Nonsense_Mutation	0,209302326	688	FALSE	0.58	Subclonal
P-0047499-T01-IM6	Pancreatic neuroendocrine tumor	Primary	ATRX	p.C1718Mfs*6	FALSE	X	76875981	ACAA	-	Frame_Shift_Del	0,090909091	286	FALSE	0.41	Subclonal
P-0047499-T01-IM6	Pancreatic neuroendocrine tumor	Primary	SETD2	p.R1459*	FALSE	3	47161751	G	A	Nonsense_Mutation	0,145118734	379	FALSE	0.4	Subclonal
P-0047499-T01-IM6	Pancreatic neuroendocrine tumor	Primary	ELF3	p.W276Hfs*22	FALSE	1	201982975	TGTGGGAG	-	Frame_Shift_Del	0,265842349	647	FALSE	0.74	Subclonal
P-0047499-T01-IM6	Pancreatic neuroendocrine tumor	Primary	MEN1	p.E471Gfs*83	FALSE	11	64572212	CGGGCTTCCTCGCCCCACGGCTCCTCGGCCT	-	Frame_Shift_Del	0,101694915	531	FALSE	0.28	Subclonal
P-0047499-T01-IM6	Pancreatic neuroendocrine tumor	Primary	MEN1	p.F93Pfs*21	FALSE	11	64577298	GCGGTGAA	-	Frame_Shift_Del	0,077571669	593	FALSE	0.21	Subclonal
P-0047499-T01-IM6	Pancreatic neuroendocrine tumor	Primary	ATRX	p.E1541Rfs*8	FALSE	X	76891468	TTGGTTTCTTCATCTTC	-	Frame_Shift_Del	0,346153846	416	FALSE	1	Clonal
P-0050209-T01-IM6	Pancreatic neuroendocrine tumor	Primary	MEN1	p.I85Sfs*33	FALSE	11	64577330	AGAC	-	Frame_Shift_Del	0,547709924	524	TRUE	1	Clonal
P-0050209-T01-IM6	Pancreatic neuroendocrine tumor	Primary	PPP4R2	p.D26G	FALSE	3	73047270	A	G	Missense_Mutation	0,468852459	305	FALSE	0.92	Clonal
P-0050209-T01-IM6	Pancreatic neuroendocrine tumor	Primary	ATRX	p.V1678F	FALSE	X	76888797	C	A	Missense_Mutation	0,721649485	291	TRUE	1	Clonal
P-0001509-T01-IM3	Pancreatic neuroendocrine tumor	Primary	MEN1	p.X266_splice	FALSE	11	64575023	C	A	Splice_Site	0.747634069400631	317	TRUE	1	Clonal
P-0001886-T01-IM3	Pancreatic neuroendocrine tumor	Primary	DAXX	p.A351P	FALSE	6	33288357	C	G	Missense_Mutation	0.690243902439024	410	TRUE	0.99	Clonal
P-0001886-T01-IM3	Pancreatic neuroendocrine tumor	Primary	MEN1	p.H46P	FALSE	11	64577445	T	G	Missense_Mutation	0.744565217391304	368	TRUE	1	Clonal
P-0001886-T01-IM3	Pancreatic neuroendocrine tumor	Primary	PTEN	p.Y76*	FALSE	10	89690819	TA	-	Frame_Shift_Del	0.44	300	FALSE	0.63	Subclonal
P-0002597-T02-IM5	Pancreatic neuroendocrine tumor	Primary	DAXX	p.H163Pfs*65	FALSE	6	33289061	AGGT	-	Frame_Shift_Del	0.314024390243902	328	FALSE	1	Clonal
P-0002597-T02-IM5	Pancreatic neuroendocrine tumor	Primary	SETD2	p.Y1604D	FALSE	3	47147516	A	C	Missense_Mutation	0.225988700564972	177	FALSE	0.91	Clonal
P-0002597-T02-IM5	Pancreatic neuroendocrine tumor	Primary	NOTCH3	p.R1981H	FALSE	19	15272497	C	T	Missense_Mutation	0.220588235294118	136	FALSE	1	Clonal
P-0003864-T01-IM5	Pancreatic neuroendocrine tumor	Primary	EPHA5	p.R7W	FALSE	4	66535442	G	A	Missense_Mutation	0.481566820276498	434	FALSE	1	Clonal
P-0003864-T01-IM5	Pancreatic neuroendocrine tumor	Primary	PIK3CG	p.H658Y	FALSE	7	106509978	C	T	Missense_Mutation	0.355848434925865	607	FALSE	0.9	Clonal
P-0003864-T01-IM5	Pancreatic neuroendocrine tumor	Primary	MLL3	p.E4863K	FALSE	7	151835937	C	T	Missense_Mutation	0.408383233532934	835	FALSE	1	Clonal
P-0003864-T01-IM5	Pancreatic neuroendocrine tumor	Primary	ARID2	p.Q798*	FALSE	12	46244298	C	T	Nonsense_Mutation	0.347297297297297	740	FALSE	0.88	Clonal
P-0003864-T01-IM5	Pancreatic neuroendocrine tumor	Primary	SH2B3	p.P166S	FALSE	12	111856445	C	T	Missense_Mutation	0.456859971711457	707	FALSE	1	Clonal
P-0003864-T01-IM5	Pancreatic neuroendocrine tumor	Primary	SH2B3	p.T172N	FALSE	12	111856464	C	A	Missense_Mutation	0.434508816120907	794	FALSE	1	Clonal
P-0003864-T01-IM5	Pancreatic neuroendocrine tumor	Primary	CD79B	p.W13*	FALSE	17	62009584	C	T	Nonsense_Mutation	0.378402903811252	1102	FALSE	0.95	Clonal
P-0004069-T01-IM5	Pancreatic neuroendocrine tumor	Primary	SETD2	p.S595*	FALSE	3	47164342	G	C	Nonsense_Mutation	0.0561224489795918	588	FALSE	0.5	Subclonal
P-0004069-T01-IM5	Pancreatic neuroendocrine tumor	Primary	IKZF1	p.X14_splice	FALSE	7	50358698	G	T	Splice_Site	0.113725490196078	255	FALSE	1	Clonal
P-0004069-T01-IM5	Pancreatic neuroendocrine tumor	Primary	MEN1	p.H204Q	FALSE	11	64575420	G	T	Missense_Mutation	0.131447587354409	601	FALSE	1	Clonal
P-0004069-T01-IM5	Pancreatic neuroendocrine tumor	Primary	MAPK3	p.R64C	FALSE	16	30133308	G	A	Missense_Mutation	0.100137174211248	729	FALSE	0.9	Clonal
P-0004069-T01-IM5	Pancreatic neuroendocrine tumor	Primary	DAXX	p.D288Mfs*6	FALSE	6	33288690	C	-	Frame_Shift_Del	0.101983002832861	353	FALSE	0.91	Clonal
P-0004069-T01-IM5	Pancreatic neuroendocrine tumor	Primary	PLK2	p.A168Lfs*4	FALSE	5	57754349	C	-	Frame_Shift_Del	0.0971428571428571	525	FALSE	0.87	Clonal
P-0004141-T01-IM5	Pancreatic neuroendocrine tumor	Primary	VHL	p.S65T	FALSE	3	10183724	T	A	Missense_Mutation	0.12463768115942	345	FALSE	0.32	Subclonal
P-0004141-T01-IM5	Pancreatic neuroendocrine tumor	Primary	MLL	p.P10L	FALSE	11	118307256	C	T	Missense_Mutation	0.276595744680851	47	FALSE	0.71	Subclonal
P-0004141-T01-IM5	Pancreatic neuroendocrine tumor	Primary	FLT1	p.R856W	FALSE	13	28908189	G	A	Missense_Mutation	0.114315139031926	971	FALSE	0.41	Subclonal
P-0004141-T01-IM5	Pancreatic neuroendocrine tumor	Primary	MEN1	p.D158Tfs*32	FALSE	11	64575560	C	-	Frame_Shift_Del	0.199029126213592	412	FALSE	0.51	Subclonal
P-0004141-T01-IM5	Pancreatic neuroendocrine tumor	Primary	PTEN	p.N117_H118del	FALSE	10	89692863	ACAATC	-	In_Frame_Del	0.335078534031414	191	FALSE	0.86	Clonal
P-0004223-T01-IM5	Pancreatic neuroendocrine tumor	Primary	TP53	p.W53*	FALSE	17	7579529	C	T	Nonsense_Mutation	0.341666666666667	120	FALSE	1	Clonal
P-0004223-T01-IM5	Pancreatic neuroendocrine tumor	Primary	SETD2	p.Q2266*	FALSE	3	47098478	G	A	Nonsense_Mutation	0.198412698412698	126	FALSE	0.65	Subclonal
P-0004223-T01-IM5	Pancreatic neuroendocrine tumor	Primary	NOTCH4	p.E1532K	FALSE	6	32166449	C	T	Missense_Mutation	0.311688311688312	154	FALSE	1	Clonal

Table S2

Sample ID	Cancer subtype	Sample type	Symbol	Aminoacid change	Hotspot	Chromosome	Position	Reference	Alternate	Mutation type	Tumor MAF	Tumor DP	LOH	Cancer cell fraction	Clonal status
P-0004223-T01-IM5	Pancreatic neuroendocrine tumor	Primary	MEN1	p.W428*	FALSE	11	64572588	C	T	Nonsense_Mutation	0.330434782608696	115	FALSE	1	Clonal
P-0004223-T01-IM5	Pancreatic neuroendocrine tumor	Primary	CBL	p.A208S	FALSE	11	119144609	G	T	Missense_Mutation	0.28	150	FALSE	0.92	Clonal
P-0004223-T01-IM5	Pancreatic neuroendocrine tumor	Primary	MLL2	p.C1510Y	FALSE	12	49440097	C	T	Missense_Mutation	0.240963855421687	166	FALSE	1	Clonal
P-0004223-T01-IM5	Pancreatic neuroendocrine tumor	Primary	TSC2	p.G1604D	FALSE	16	2136342	G	A	Missense_Mutation	0.28	100	FALSE	0.92	Clonal
P-0004223-T01-IM5	Pancreatic neuroendocrine tumor	Primary	SMAD4	p.X83_splice	FALSE	18	48573666	G	A	Splice_Site	0.227848101265823	79	FALSE	0.75	Subclonal
P-0004223-T01-IM5	Pancreatic neuroendocrine tumor	Primary	DAXX	p.A406Rfs*11	FALSE	6	33288192	CA	-	Frame_Shift_Del	0.259541984732824	131	FALSE	0.85	Clonal
P-0005922-T01-IM5	Pancreatic neuroendocrine tumor	Primary	DAXX	p.E104*	FALSE	6	33289242	C	A	Nonsense_Mutation	0.554376657824934	754	TRUE	1	Clonal
P-0005922-T01-IM5	Pancreatic neuroendocrine tumor	Primary	MLL3	p.T3884Sfs*5	FALSE	7	151855967	G	-	Frame_Shift_Del	0.110994764397906	955	FALSE	0.33	Subclonal
P-0005922-T01-IM5	Pancreatic neuroendocrine tumor	Primary	MEN1	p.C426Afs*24	FALSE	11	64572596	G	-	Frame_Shift_Del	0.540136054421769	735	TRUE	1	Clonal
P-0007355-T01-IM5	Pancreatic neuroendocrine tumor	Primary	MEN1	p.X310_splice	FALSE	11	64573828	CTGAGGCAATGCCCTGGATGGAGGTGAGGCAGAGGATCCTCAGGGAGGCA GCCCCAGCTGCCCTGCTGGCACAAATGCCCCACCAGGGCACACCCAGAAG GGGCCACAGGAAGATCCCAGGGAGTCCT	-	Splice_Site	0.600522193211488	383	TRUE	1	Clonal
P-0008162-T02-IM5	Pancreatic neuroendocrine tumor	Primary	PLK2	p.D609G	FALSE	5	57750778	T	C	Missense_Mutation	0.0584343991179713	907	FALSE	0.32	Subclonal
P-0008799-T01-IM5	Pancreatic neuroendocrine tumor	Primary	VHL	p.R167Q	FALSE	3	10191507	G	A	Missense_Mutation	0.266071428571429	560	TRUE	0.92	Clonal
P-0009515-T01-IM5	Pancreatic neuroendocrine tumor	Primary	TP53	p.R248W	TRUE	17	7577539	G	A	Missense_Mutation	0.508532423208191	586	TRUE	1	Clonal
P-0009515-T01-IM5	Pancreatic neuroendocrine tumor	Primary	KRAS	p.G12R	TRUE	12	25398285	C	G	Missense_Mutation	0.359002169197397	922	FALSE	1	Clonal
P-0009515-T01-IM5	Pancreatic neuroendocrine tumor	Primary	RB1	p.R556*	FALSE	13	48955550	C	T	Nonsense_Mutation	0.477157360406091	788	TRUE	1	Clonal
P-0009515-T01-IM5	Pancreatic neuroendocrine tumor	Primary	MED12	p.W257C	FALSE	X	70341212	G	T	Missense_Mutation	0.263115415657789	1239	FALSE	0.75	Subclonal
P-0010652-T01-IM5	Pancreatic neuroendocrine tumor	Primary	MEN1	p.X456_splice	FALSE	11	64572289	C	T	Splice_Site	0.778508771929825	912	TRUE	1	Clonal
P-0010652-T01-IM5	Pancreatic neuroendocrine tumor	Primary	JAK3	p.E739K	FALSE	19	17945515	C	T	Missense_Mutation	0.403916768665851	817	FALSE	0.96	Clonal
P-0010652-T01-IM5	Pancreatic neuroendocrine tumor	Primary	DAXX	p.M470_Q471delinsI*	FALSE	6	33287842	GC	AT	Nonsense_Mutation	0.773584905660377	901	TRUE	1	Clonal
P-0010692-T01-IM5	Pancreatic neuroendocrine tumor	Primary	TSC1	p.R484Efs*48	FALSE	9	135781515	TA	G	Frame_Shift_Del	0.823076923076923	1300	TRUE	1	Clonal
P-0011405-T01-IM5	Pancreatic neuroendocrine tumor	Primary	IKZF1	p.D436N	FALSE	7	50468071	G	A	Missense_Mutation	0.353826850690088	797	FALSE	0.81	Subclonal
P-0011405-T01-IM5	Pancreatic neuroendocrine tumor	Primary	NTRK2	p.N125S	FALSE	9	87322773	A	G	Missense_Mutation	0.423349056603774	848	FALSE	0.97	Clonal
P-0011405-T01-IM5	Pancreatic neuroendocrine tumor	Primary	TSC2	p.V1547D	FALSE	16	2135301	T	A	Missense_Mutation	0.786585365853659	656	TRUE	1	Clonal
P-0011405-T01-IM5	Pancreatic neuroendocrine tumor	Primary	PRKAR1A	p.L50Gfs*24	FALSE	17	66511686	TCCTCAGGGAATACTTTGAGAGG	-	Frame_Shift_Del	0.718146718146718	259	FALSE	0.93	Clonal
P-0011464-T01-IM5	Pancreatic neuroendocrine tumor	Primary	DAXX	p.L134P	FALSE	6	33289151	A	G	Missense_Mutation	0.656964656964657	481	TRUE	1	Clonal
P-0011464-T01-IM5	Pancreatic neuroendocrine tumor	Primary	PRDM1	p.R679H	FALSE	6	106554919	G	A	Missense_Mutation	0.0515873015873016	504	FALSE	0.15	Subclonal
P-0011464-T01-IM5	Pancreatic neuroendocrine tumor	Primary	MEN1	p.X309_splice	FALSE	11	64574482	C	T	Splice_Site	0.636887608069164	347	TRUE	1	Clonal
P-0011479-T01-IM5	Pancreatic neuroendocrine tumor	Primary	INPP4B	p.H345R	FALSE	4	143129616	T	C	Missense_Mutation	0.185671039354188	991	FALSE	0.64	Subclonal
P-0011479-T01-IM5	Pancreatic neuroendocrine tumor	Primary	DAXX	p.D738V	FALSE	6	33286530	T	A	Missense_Mutation	0.276315789473684	456	FALSE	1	Clonal
P-0012231-T01-IM5	Pancreatic neuroendocrine tumor	Primary	VHL	p.T105_H110del	FALSE	3	10183843	CACGGGCCGCCGCATCCA	-	In_Frame_Del	0.101123595505618	178	FALSE	0.35	Subclonal
P-0012231-T01-IM5	Pancreatic neuroendocrine tumor	Primary	VHL	p.F136Lfs*20	FALSE	3	10188265	TGTGCCATCTC	G	Frame_Shift_Del	0.173295454545455	704	FALSE	0.47	Subclonal
P-0012382-T01-IM5	Pancreatic neuroendocrine tumor	Primary	TERT	p.R979W	FALSE	5	1260624	G	A	Missense_Mutation	0.349320543565148	1251	FALSE	0.97	Clonal
P-0012382-T01-IM5	Pancreatic neuroendocrine tumor	Primary	MEN1	p.C246W	FALSE	11	64575084	A	C	Missense_Mutation	0.577395577395577	814	TRUE	1	Clonal
P-0012382-T01-IM5	Pancreatic neuroendocrine tumor	Primary	RB1	p.E313*	FALSE	13	48939105	G	T	Nonsense_Mutation	0.529411764705882	340	TRUE	0.94	Clonal
P-0013555-T01-IM5	Pancreatic neuroendocrine tumor	Primary	TSC2	p.Q785*	FALSE	16	2122982	C	T	Nonsense_Mutation	0.160664819944598	361	FALSE	0.6	Subclonal
P-0013555-T01-IM5	Pancreatic neuroendocrine tumor	Primary	TSC2	p.X1295_splice	FALSE	16	2133694	A	G	Splice_Site	0.140522875816993	306	FALSE	0.52	Subclonal
P-0014312-T02-IM6	Pancreatic neuroendocrine tumor	Primary	PIK3CG	p.G343S	FALSE	7	106509033	G	A	Missense_Mutation	0.0590015128593041	661	FALSE	0.22	Subclonal
P-0014549-T01-IM6	Pancreatic neuroendocrine tumor	Primary	RFWD2	p.L708P	FALSE	1	175956089	A	G	Missense_Mutation	0.220588235294118	544	FALSE	0.82	Clonal
P-0015330-T01-IM6	Pancreatic neuroendocrine tumor	Primary	MEN1	p.Q7*	FALSE	11	64577563	G	A	Nonsense_Mutation	0.538461538461538	247	TRUE	1	Clonal
P-0015330-T01-IM6	Pancreatic neuroendocrine tumor	Primary	SETD2	p.C1499Wfs*6	FALSE	3	47158150	CACATGCTATTTCACCTTGAGCTCTTTCATCTTTAGAAAGAGGTGTACACTCA	-	Frame_Shift_Del	0.177644710578842	501	FALSE	0.73	Subclonal
P-0015330-T01-IM6	Pancreatic neuroendocrine tumor	Primary	DAXX	p.R590Gfs*12	FALSE	6	33287330	G	-	Frame_Shift_Del	0.31390134529148	446	FALSE	0.97	Clonal
P-0016901-T01-IM6	Pancreatic neuroendocrine tumor	Primary	TSC2	p.F1803Lfs*23	FALSE	16	2138596	C	-	Frame_Shift_Del	0.654205607476635	642	TRUE	1	Clonal
P-0016901-T01-IM6	Pancreatic neuroendocrine tumor	Primary	DAXX	p.Q255*	FALSE	6	33288789	G	A	Nonsense_Mutation	0.569979716024341	493	TRUE	0.99	Clonal
P-0016901-T01-IM6	Pancreatic neuroendocrine tumor	Primary	KMT2C	p.Q663*	FALSE	7	151945532	G	A	Nonsense_Mutation	0.0566502463054187	406	FALSE	0.15	Subclonal
P-0016901-T01-IM6	Pancreatic neuroendocrine tumor	Primary	PTPRD	p.K1031N	FALSE	9	8485287	C	G	Missense_Mutation	0.508417508417508	297	FALSE	0.88	Clonal
P-0017012-T01-IM6	Pancreatic neuroendocrine tumor	Primary	EPAS1	p.P531S	FALSE	2	46607402	C	T	Missense_Mutation	0.263205013428827	1117	FALSE	0.98	Clonal
P-0017466-T01-IM6	Pancreatic neuroendocrine tumor	Primary	TP53	p.R213*	FALSE	17	7578212	G	A	Nonsense_Mutation	0.0312046444121916	1378	FALSE	0.15	Subclonal
P-0017466-T01-IM6	Pancreatic neuroendocrine tumor	Primary	TP53	p.L130V	TRUE	17	7578542	G	C	Missense_Mutation	0.207080828323313	1497	FALSE	0.98	Clonal
P-0017466-T01-IM6	Pancreatic neuroendocrine tumor	Primary	SESN3	p.F379S	FALSE	11	94910994	A	G	Missense_Mutation	0.759146341463415	656	FALSE	1	Clonal
P-0017466-T01-IM6	Pancreatic neuroendocrine tumor	Primary	TSC2	p.X741_splice	FALSE	16	2122849	G	A	Splice_Site	0.0512820512820513	546	TRUE	0.14	Subclonal
P-0017466-T01-IM6	Pancreatic neuroendocrine tumor	Primary	TSC2	p.X1204_splice	FALSE	16	2131595	G	A	Splice_Site	0.0893854748603352	537	TRUE	0.24	Subclonal
P-0017466-T01-IM6	Pancreatic neuroendocrine tumor	Primary	SMAD4	p.P218Lfs*23	FALSE	18	48581348	C	-	Frame_Shift_Del	0.78646934460888	473	TRUE	1	Clonal
P-0017466-T01-IM6	Pancreatic neuroendocrine tumor	Primary	DAXX	p.E731*	FALSE	6	33286552	C	A	Nonsense_Mutation	0.72113676731794	563	TRUE	1	Clonal
P-0019112-T01-IM6	Pancreatic neuroendocrine tumor	Primary	MEN1	p.F415Sfs*35	FALSE	11	64572627	A	-	Frame_Shift_Del	0.630769230769231	455	TRUE	1	Clonal
P-0019122-T01-IM6	Pancreatic neuroendocrine tumor	Primary	BCL2L1	p.S43L	FALSE	20	30309894	G	A	Missense_Mutation	0.0830860534124629	1011	FALSE	0.31	Subclonal
P-0019275-T01-IM6	Pancreatic neuroendocrine tumor	Primary	MEN1	p.G149Vfs*5	FALSE	11	64577136	C	-	Frame_Shift_Del	0.341945288753799	658	TRUE	1	Clonal
P-0019275-T01-IM6	Pancreatic neuroendocrine tumor	Primary	TSC2	p.X374_splice	FALSE	16	2111865	ACTGCAG	-	Splice_Site	0.372413793103448	435	TRUE	1	Clonal
P-0019275-T01-IM6	Pancreatic neuroendocrine tumor	Primary	DAXX	p.Y648*	FALSE	6	33286993	-	TAGC	Nonsense_Mutation	0.341040462427746	519	TRUE	1	Clonal
P-0019367-T01-IM6	Pancreatic neuroendocrine tumor	Primary	RFWD2	p.E280K	FALSE	1	176105677	C	T	Missense_Mutation	0.173024523160763	734	FALSE	0.95	Clonal
P-0019367-T01-IM6	Pancreatic neuroendocrine tumor	Primary	MEN1	p.L272*	FALSE	11	64574673	AGAGC	-	Frame_Shift_Del	0.426592797783933	722	TRUE	1	Clonal
P-0019367-T01-IM6	Pancreatic neuroendocrine tumor	Primary	KMT2D	p.E905V	FALSE	12	49444752	T	A	Missense_Mutation	0.171676686706747	1497	FALSE	0.6	Subclonal
P-0019367-T01-IM6	Pancreatic neuroendocrine tumor	Primary	STAG2	p.Q76L	FALSE	X	123164914	A	T	Missense_Mutation	0.129963898916967	554	FALSE	0.25	Subclonal
P-0019506-T01-IM6	Pancreatic neuroendocrine tumor	Primary	MEN1	p.L272R	FALSE	11	64574675	A	C	Missense_Mutation	0.678571428571429	364	TRUE	1	Clonal
P-0020349-T01-IM6	Pancreatic neuroendocrine tumor	Primary	TERT	p.promoter	FALSE	5	1295250	G	A	5'Flank	0.673228346456693	254	TRUE	1	Clonal
P-0020349-T01-IM6	Pancreatic neuroendocrine tumor	Primary	KMT2D	p.Q4103*	FALSE	12	49426181	G	A	Nonsense_Mutation	0.383367139959432	493	FALSE	0.95	Clonal
P-0020684-T01-IM6	Pancreatic neuroendocrine tumor	Primary	MEN1	p.V50Afs*67	FALSE	11	64577427	CGGTTGA	-	Frame_Shift_Del	0.491758241758242	728	TRUE	0.93	Clonal
P-0020684-T01-IM6	Pancreatic neuroendocrine tumor	Primary	MRE11A	p.M84I	FALSE	11	94219152	C	T	Missense_Mutation	0.564207650273224	732	FALSE	1	Clonal
P-0020684-T01-IM6	Pancreatic neuroendocrine tumor	Primary	SETD2	p.C1501Y	FALSE	3	47158197	C	T	Missense_Mutation	0.547826086956522	690	TRUE	1	Clonal
P-0021222-T01-IM6	Pancreatic neuroendocrine tumor	Primary	DAXX	p.L311_M312del	FALSE	6	33288616	CATGAG	-	In_Frame_Del	0.814889336016097	497	TRUE	1	Clonal
P-0021768-T01-IM6	Pancreatic neuroendocrine tumor	Primary	RASA1	p.P538S	FALSE	5	86665631	C	T	Missense_Mutation	0.409937888198758	161	FALSE	1	Clonal
P-0021768-T01-IM6	Pancreatic neuroendocrine tumor	Primary	SMAD2	p.K420E	FALSE	18	45371733	T	C	Missense_Mutation	0.402730375426621	293	FALSE	1	Clonal
P-0021917-T01-IM6	Pancreatic neuroendocrine tumor	Primary	TSC2	p.S701_D702insF	FALSE	16	2122246	-	TTT	In_Frame_Ins	0.48922056384743	603	TRUE	1	Clonal
P-0021917-T01-IM6	Pancreatic neuroendocrine tumor	Primary	DAXX	p.Y59*	FALSE	6	33289526	G	C	Nonsense_Mutation	0.527683615819209	885	TRUE	1	Clonal
P-0023400-T01-IM6	Pancreatic neuroendocrine tumor	Primary	IRS1	p.L86V	FALSE	2	227663199	G	C	Missense_Mutation	0.111111111111111	999	FALSE	0.26	Subclonal
P-0023400-T01-IM6	Pancreatic neuroendocrine tumor	Primary	VHL	p.V165D	FALSE	3	10191501	T	A	Missense_Mutation	0.326295585412668	521	TRUE	0.77	Subclonal
P-0023400-T01-IM6	Pancreatic neuroendocrine tumor	Primary	WHSC1	p.L695F	FALSE	4	1953904	C	T	Missense_Mutation	0.0533562822719449	1162	FALSE	0.13	Subclonal
P-0023717-T01-IM6	Pancreatic neuroendocrine tumor	Primary	MEN1	p.I85Yfs*32	FALSE	11	64577329	-	A	Frame_Shift_Ins	0.307407407407407	810	TRUE	0.91	Clonal
P-0023717-T01-IM6	Pancreatic neuroendocrine tumor	Primary	TSC2	p.I1614del	FALSE	16	2136370	CAT	-	In_Frame_Del	0.263452914798206	892	TRUE	0.78	Subclonal
P-0023717-T01-IM6	Pancreatic neuroendocrine tumor	Primary	DAXX	p.Q383*	FALSE	6	33288261	G	A	Nonsense_Mutation	0.306954436450839	834	TRUE	0.91	Clonal

Table S2

Sample ID	Cancer subtype	Sample type	Symbol	Aminoacid change	Hotspot	Chromosome	Position	Reference	Alternate	Mutation type	Tumor MAF	Tumor DP	LOH	Cancer cell fraction	Clonal status
P-0023732-T01-IM6	Pancreatic neuroendocrine tumor	Primary	DAXX	p.C106*	FALSE	6	33289234	G	T	Nonsense_Mutation	0.64030612244898	392	FALSE	1	Clonal
P-0023732-T01-IM6	Pancreatic neuroendocrine tumor	Primary	MEN1	p.X456_splice	FALSE	11	64572290	T	A	Splice_Site	0.697104677060134	449	TRUE	1	Clonal
P-0023732-T01-IM6	Pancreatic neuroendocrine tumor	Primary	MET	p.G756C	FALSE	7	116399446	G	T	Missense_Mutation	0.655555555555556	360	TRUE	1	Clonal
P-0024533-T01-IM6	Pancreatic neuroendocrine tumor	Primary	DAXX	p.Q177*	FALSE	6	33289023	G	A	Nonsense_Mutation	0.511066398390342	497	TRUE	1	Clonal
P-0024533-T01-IM6	Pancreatic neuroendocrine tumor	Primary	SMO	p.M781I	FALSE	7	128852271	G	A	Missense_Mutation	0.330345710627401	781	FALSE	0.99	Clonal
P-0024594-T01-IM6	Pancreatic neuroendocrine tumor	Primary	BAP1	p.R701C	FALSE	3	52436393	G	A	Missense_Mutation	0.329192546583851	644	FALSE	1	Clonal
P-0024594-T01-IM6	Pancreatic neuroendocrine tumor	Primary	ARID1A	p.X641_splice	FALSE	1	27087346	G	A	Splice_Site	0.108843537414966	147	TRUE	0.31	Subclonal
P-0024594-T01-IM6	Pancreatic neuroendocrine tumor	Primary	SETD2	p.G2046V	FALSE	3	47103808	GC	AA	Missense_Mutation	0.100334448160535	299	TRUE	0.39	Subclonal
P-0024594-T01-IM6	Pancreatic neuroendocrine tumor	Primary	HIST1H3I	p.A22D	FALSE	6	27840029	G	T	Missense_Mutation	0.0833333333333333	492	FALSE	0.41	Subclonal
P-0024594-T01-IM6	Pancreatic neuroendocrine tumor	Primary	BTK	p.L128R	FALSE	X	100624994	A	C	Missense_Mutation	0.0516129032258065	310	FALSE	0.14	Subclonal
P-0026857-T01-IM6	Pancreatic neuroendocrine tumor	Primary	NRAS	p.Q61R	TRUE	1	115256529	T	C	Missense_Mutation	0.25	632	FALSE	0.5	Subclonal
P-0026857-T01-IM6	Pancreatic neuroendocrine tumor	Primary	CTNNB1	p.S37A	TRUE	3	41266112	T	G	Missense_Mutation	0.243956043956044	455	FALSE	0.49	Subclonal
P-0027191-T01-IM6	Pancreatic neuroendocrine tumor	Primary	ARID1A	p.C2081*	FALSE	1	27106632	C	A	Nonsense_Mutation	0.226190476190476	756	FALSE	1	Clonal
P-0027191-T01-IM6	Pancreatic neuroendocrine tumor	Primary	ATM	p.T1953I	FALSE	11	108180982	C	T	Missense_Mutation	0.349112426035503	338	FALSE	1	Clonal
P-0027191-T01-IM6	Pancreatic neuroendocrine tumor	Primary	MGA	p.L636Ffs*21	FALSE	15	41989112	-	T	Frame_Shift_Ins	0.293370944992948	709	FALSE	1	Clonal
P-0027191-T01-IM6	Pancreatic neuroendocrine tumor	Primary	SMAD4	p.V163M	FALSE	18	48581183	G	A	Missense_Mutation	0.168905950095969	521	FALSE	0.75	Subclonal
P-0027620-T01-IM6	Pancreatic neuroendocrine tumor	Primary	ATR	p.M2058T	FALSE	3	142204030	A	G	Missense_Mutation	0.467075038284839	653	FALSE	0.93	Clonal
P-0028760-T01-IM6	Pancreatic neuroendocrine tumor	Primary	MEN1	p.V35Cfs*84	FALSE	11	64577479	C	-	Frame_Shift_Del	0.450738916256158	406	TRUE	0.9	Clonal
P-0029781-T01-IM6	Pancreatic neuroendocrine tumor	Primary	KDM6A	p.R1351*	FALSE	X	44969369	C	T	Nonsense_Mutation	0.356382978723404	188	FALSE	1	Clonal
P-0029781-T01-IM6	Pancreatic neuroendocrine tumor	Primary	RRAGC	p.G50D	FALSE	1	39325170	C	T	Missense_Mutation	0.306211723534558	1143	FALSE	0.61	Subclonal
P-0029781-T01-IM6	Pancreatic neuroendocrine tumor	Primary	MEN1	p.L48Wfs*71	FALSE	11	64577441	A	-	Frame_Shift_Del	0.670094936708861	1264	TRUE	1	Clonal
P-0029781-T01-IM6	Pancreatic neuroendocrine tumor	Primary	IRS2	p.G1292S	FALSE	13	110434527	C	T	Missense_Mutation	0.181871005229518	1721	FALSE	0.55	Subclonal
P-0029781-T01-IM6	Pancreatic neuroendocrine tumor	Primary	GNAQ	p.M7I	FALSE	9	80646131	C	A	Missense_Mutation	0.25	200	FALSE	0.5	Subclonal
P-0030513-T01-IM6	Pancreatic neuroendocrine tumor	Primary	TP53	p.P191del	FALSE	17	7578275	GAG	-	In_Frame_Del	0.200708382526564	847	FALSE	0.81	Subclonal
P-0030513-T01-IM6	Pancreatic neuroendocrine tumor	Primary	RB1	p.R418Sfs*9	FALSE	13	48951087	AA	-	Frame_Shift_Del	0.187683284457478	341	FALSE	0.76	Subclonal
P-0030513-T01-IM6	Pancreatic neuroendocrine tumor	Primary	TGFBR2	p.K153Afs*3	FALSE	3	30691872	AA	-	Frame_Shift_Del	0.227272727272727	462	FALSE	0.92	Clonal
P-0030513-T01-IM6	Pancreatic neuroendocrine tumor	Primary	BRD4	p.A1189V	FALSE	19	15350213	G	A	Missense_Mutation	0.224902723735409	1285	FALSE	0.91	Clonal
P-0030513-T01-IM6	Pancreatic neuroendocrine tumor	Primary	BCL6	p.R26W	FALSE	3	187451406	G	A	Missense_Mutation	0.250345781466113	723	FALSE	1	Clonal
P-0030513-T01-IM6	Pancreatic neuroendocrine tumor	Primary	DNMT3B	p.L454Afs*11	FALSE	20	31384650	-	G	Frame_Shift_Ins	0.0757575757575758	1188	FALSE	0.38	Subclonal
P-0030513-T01-IM6	Pancreatic neuroendocrine tumor	Primary	MSH3	p.K383Rfs*32	FALSE	5	79970915	A	-	Frame_Shift_Del	0.186094069529652	978	FALSE	0.75	Subclonal
P-0030513-T01-IM6	Pancreatic neuroendocrine tumor	Primary	TSC2	p.S1514*	FALSE	16	2134999	C	A	Nonsense_Mutation	0.248419150858175	1107	FALSE	1	Clonal
P-0030513-T01-IM6	Pancreatic neuroendocrine tumor	Primary	BCL2	p.L169M	FALSE	18	60985395	G	T	Missense_Mutation	0.112359550561798	1157	FALSE	0.45	Subclonal
P-0030513-T01-IM6	Pancreatic neuroendocrine tumor	Primary	CARM1	p.N311I	FALSE	19	11027167	A	T	Missense_Mutation	0.127642913077525	1277	FALSE	0.51	Subclonal
P-0030513-T01-IM6	Pancreatic neuroendocrine tumor	Primary	DAXX	p.X489_splice	FALSE	6	33287787	C	T	Splice_Site	0.252365930599369	1268	FALSE	1	Clonal
P-0030778-T01-IM6	Pancreatic neuroendocrine tumor	Primary	TP53	p.H179D	TRUE	17	7578395	G	C	Missense_Mutation	0.291390728476821	755	FALSE	1	Clonal
P-0030778-T01-IM6	Pancreatic neuroendocrine tumor	Primary	TSC2	p.P1675L	FALSE	16	2137898	C	T	Missense_Mutation	0.424198250728863	686	TRUE	1	Clonal
P-0030778-T01-IM6	Pancreatic neuroendocrine tumor	Primary	MEN1	p.X279_splice	FALSE	11	64574642	TCTACTGACCTT	-	Splice_Site	0.395470383275261	574	TRUE	1	Clonal
P-0030778-T01-IM6	Pancreatic neuroendocrine tumor	Primary	ARID2	p.Q894E	FALSE	12	46244586	C	G	Missense_Mutation	0.299565846599132	691	FALSE	1	Clonal
P-0030778-T01-IM6	Pancreatic neuroendocrine tumor	Primary	CREBBP	p.Q881*	FALSE	16	3820810	G	A	Nonsense_Mutation	0.412698412698413	819	TRUE	1	Clonal
P-0030778-T01-IM6	Pancreatic neuroendocrine tumor	Primary	WHSC1	p.R1353Q	FALSE	4	1980596	G	A	Missense_Mutation	0.294502617801047	764	FALSE	1	Clonal
P-0030778-T01-IM6	Pancreatic neuroendocrine tumor	Primary	PLK2	p.D594G	FALSE	5	57750823	T	C	Missense_Mutation	0.286486486486487	555	FALSE	1	Clonal
P-0030778-T01-IM6	Pancreatic neuroendocrine tumor	Primary	DAXX	p.G250*	FALSE	6	33288796	GACACGGCCG	-	Frame_Shift_Del	0.319047619047619	420	TRUE	0.84	Clonal
P-0031556-T01-IM6	Pancreatic neuroendocrine tumor	Primary	ERBB4	p.R927*	FALSE	2	212288967	G	A	Nonsense_Mutation	0.400398406374502	502	TRUE	0.97	Clonal
P-0031556-T01-IM6	Pancreatic neuroendocrine tumor	Primary	MTOR	p.L2334V	FALSE	1	11177077	A	C	Missense_Mutation	0.394683026584867	489	FALSE	0.95	Clonal
P-0031556-T01-IM6	Pancreatic neuroendocrine tumor	Primary	MGA	p.X1282_splice	FALSE	15	42026718	A	G	Splice_Site	0.0748440748440748	481	FALSE	0.18	Subclonal
P-0031556-T01-IM6	Pancreatic neuroendocrine tumor	Primary	DAXX	p.S641*	FALSE	6	33287175	G	C	Nonsense_Mutation	0.053484602917342	617	TRUE	0.13	Subclonal
P-0031556-T01-IM6	Pancreatic neuroendocrine tumor	Primary	DAXX	p.L330P	FALSE	6	33288563	A	G	Missense_Mutation	0.416206261510129	543	TRUE	1	Clonal
P-0033899-T01-IM6	Pancreatic neuroendocrine tumor	Primary	TP53	p.P82L	TRUE	17	7579442	G	A	Missense_Mutation	0.020671834625323	387	FALSE	0.06	Subclonal
P-0033899-T01-IM6	Pancreatic neuroendocrine tumor	Primary	TEK	p.A1086S	FALSE	9	27228259	G	T	Missense_Mutation	0.351063829787234	282	FALSE	0.7	Subclonal
P-0035279-T01-IM6	Pancreatic neuroendocrine tumor	Primary	MEN1	p.W188R	FALSE	11	64575470	A	T	Missense_Mutation	0.279527559055118	762	FALSE	1	Clonal
P-0035279-T01-IM6	Pancreatic neuroendocrine tumor	Primary	SETD2	p.E397*	FALSE	3	47164937	C	A	Nonsense_Mutation	0.189054726368159	402	FALSE	0.67	Subclonal
P-0035279-T01-IM6	Pancreatic neuroendocrine tumor	Primary	DAXX	p.X347_splice	FALSE	6	33288369	C	T	Splice_Site	0.305194805194805	462	FALSE	1	Clonal
P-0036906-T01-IM6	Pancreatic neuroendocrine tumor	Primary	MEN1	p.Q344*	FALSE	11	64573738	G	A	Nonsense_Mutation	0.0678336980306346	457	TRUE	0.12	Subclonal
P-0036906-T01-IM6	Pancreatic neuroendocrine tumor	Primary	DAXX	p.P179Qfs*50	FALSE	6	33289016	G	-	Frame_Shift_Del	0.608988764044944	445	TRUE	1	Clonal
P-0036906-T01-IM6	Pancreatic neuroendocrine tumor	Primary	MEN1	p.E292Dfs*23	FALSE	11	64574514	CCAGGGGTGGGCTCCAGCTCC	A	Frame_Shift_Del	0.418316831683168	404	TRUE	0.71	Subclonal
P-0037527-T01-IM6	Pancreatic neuroendocrine tumor	Primary	PTEN	p.R335*	FALSE	10	89720852	C	T	Nonsense_Mutation	0.356037151702786	323	TRUE	1	Clonal
P-0037527-T01-IM6	Pancreatic neuroendocrine tumor	Primary	RET	p.L790F	FALSE	10	43613906	G	T	Missense_Mutation	0.107142857142857	560	FALSE	0.57	Subclonal
P-0037527-T01-IM6	Pancreatic neuroendocrine tumor	Primary	MEN1	p.W476*	FALSE	11	64572227	C	T	Nonsense_Mutation	0.670611439842209	507	TRUE	1	Clonal
P-0037527-T01-IM6	Pancreatic neuroendocrine tumor	Primary	NKX2-1	p.F210L	FALSE	14	36987059	G	C	Missense_Mutation	0.173770491803279	915	FALSE	0.58	Subclonal
P-0037527-T01-IM6	Pancreatic neuroendocrine tumor	Primary	DAXX	p.X418_splice	FALSE	6	33288002	C	T	Splice_Site	0.683116883116883	385	TRUE	1	Clonal
P-0043954-T01-IM6	Pancreatic neuroendocrine tumor	Primary	MEN1	p.F23Lfs*96	FALSE	11	64577513	A	-	Frame_Shift_Del	0.534883720930233	387	TRUE	0.73	Subclonal
P-0044029-T01-IM6	Pancreatic neuroendocrine tumor	Primary	MEN1	p.I85Sfs*33	FALSE	11	64577330	AGAC	-	Frame_Shift_Del	0.313868613138686	548	TRUE	1	Clonal
P-0044029-T01-IM6	Pancreatic neuroendocrine tumor	Primary	ARID2	p.G1110E	FALSE	12	46245235	G	A	Missense_Mutation	0.0971337579617834	628	FALSE	0.42	Subclonal
P-0044029-T01-IM6	Pancreatic neuroendocrine tumor	Primary	SETD2	p.C667Vfs*3	FALSE	3	47164127	A	-	Frame_Shift_Del	0.218106995884774	243	FALSE	0.73	Subclonal
P-0044062-T01-IM6	Pancreatic neuroendocrine tumor	Primary	PTPRT	p.R328H	FALSE	20	41306676	C	T	Missense_Mutation	0.0583700440528634	908	FALSE	0.14	Subclonal
P-0044062-T01-IM6	Pancreatic neuroendocrine tumor	Primary	MEN1	p.D70Tfs*49	FALSE	11	64577375	G	-	Frame_Shift_Del	0.735537190082645	484	TRUE	1	Clonal
P-0044604-T01-IM6	Pancreatic neuroendocrine tumor	Primary	NOTCH1	p.T518M	FALSE	9	139411726	G	A	Missense_Mutation	0.281609195402299	348	FALSE	0.58	Subclonal
P-0044604-T01-IM6	Pancreatic neuroendocrine tumor	Primary	MEN1	p.R98*	FALSE	11	64577290	G	A	Nonsense_Mutation	0.670781893004115	486	TRUE	1	Clonal
P-0044604-T01-IM6	Pancreatic neuroendocrine tumor	Primary	DAXX	p.K591Rfs*8	FALSE	6	33287316	TCTGATTGCT	-	Frame_Shift_Del	0.724489795918367	196	TRUE	1	Clonal
P-0046693-T01-IM6	Pancreatic neuroendocrine tumor	Primary	GATA2	p.G468S	FALSE	3	128199903	C	T	Missense_Mutation	0.457253886010363	772	FALSE	1	Clonal
P-0047293-T01-IM6	Pancreatic neuroendocrine tumor	Primary	MEN1	p.D423Y	FALSE	11	64572604	C	A	Missense_Mutation	0.661399548532731	443	TRUE	0.9	Clonal
P-0043063-T01-IM6	Pancreatic neuroendocrine tumor	Primary	TP53	p.C275Y	TRUE	17	7577114	C	T	Missense_Mutation	0.4141689373297	1101	FALSE	0.98	Clonal
P-0043063-T01-IM6	Pancreatic neuroendocrine tumor	Primary	MALT1	p.D471N	FALSE	18	56401549	G	A	Missense_Mutation	0.0563758389261745	745	FALSE	0.13	Subclonal
P-0043063-T01-IM6	Pancreatic neuroendocrine tumor	Primary	MEN1	p.I85Sfs*33	FALSE	11	64577330	AGAC	-	Frame_Shift_Del	0.689149560117302	682	TRUE	0.94	Clonal
P-0043063-T01-IM6	Pancreatic neuroendocrine tumor	Primary	AR	p.G459_G473del	FALSE	X	66766357	GGCGGCGGCGGCGGCGGCGGCGGCGGCGGCGGCGGCGGCGGCGGC	-	In_Frame_Del	0.305555555555556	288	FALSE	1	Clonal
P-0011163-T01-IM5	Pancreatic neuroendocrine tumor	Primary	FGFR2	p.R190Q	FALSE	10	123310859	C	T	Missense_Mutation	0,161698284	1107	FALSE	0.38	Subclonal
P-0001313-T02-IM6	Pancreatic neuroendocrine tumor	Metastasis	ATM	p.R337H	TRUE	11	108117799	G	A	Missense_Mutation	0,778029445	883	FALSE	1	Clonal
P-0001313-T02-IM6	Pancreatic neuroendocrine tumor	Metastasis	TSC2	p.H1506R	FALSE	16	2134975	A	G	Missense_Mutation	0,679648241	796	TRUE	1	Clonal
P-0001313-T02-IM6	Pancreatic neuroendocrine tumor	Metastasis	AURKA	p.S388*	FALSE	20	54945263	G	C	Nonsense_Mutation	0,377125193	647	FALSE	1	Clonal
P-0001313-T02-IM6	Pancreatic neuroendocrine tumor	Metastasis	MEN1	p.R521Gfs*43	FALSE	11	64572093	G	-	Frame_Shift_Del	0,604490501	579	FALSE	1	Clonal
P-0001313-T02-IM6	Pancreatic neuroendocrine tumor	Metastasis	RNF43	p.P116S	FALSE	17	56448301	G	A	Missense_Mutation	0,238095238	924	FALSE	1	Clonal

Table S2

Sample ID	Cancer subtype	Sample type	Symbol	Aminoacid change	Hotspot	Chromosome	Position	Reference	Alternate	Mutation type	Tumor MAF	Tumor DP	LOH	Cancer cell fraction	Clonal status
P-0001313-T02-IM6	Pancreatic neuroendocrine tumor	Metastasis	PRKAR1A	p.T75I	FALSE	17	66518943	C	T	Missense_Mutation	0,20668693	658	FALSE	0.89	Clonal
P-0001313-T02-IM6	Pancreatic neuroendocrine tumor	Metastasis	TSC1	p.T578S	FALSE	9	135781233	T	A	Missense_Mutation	0,235213205	727	FALSE	1	Clonal
P-0004026-T01-IM5	Pancreatic neuroendocrine tumor	Metastasis	MDC1	p.V1935I	FALSE	6	30670943	C	T	Missense_Mutation	0,163722026	849	FALSE	0.53	Subclonal
P-0004026-T01-IM5	Pancreatic neuroendocrine tumor	Metastasis	ARID5B	p.D637N	FALSE	10	63851131	G	A	Missense_Mutation	0,121593291	477	FALSE	0.4	Subclonal
P-0004026-T01-IM5	Pancreatic neuroendocrine tumor	Metastasis	RARA	p.K164T	FALSE	17	38508183	A	C	Missense_Mutation	0,072727273	990	FALSE	0.31	Subclonal
P-0004026-T01-IM5	Pancreatic neuroendocrine tumor	Metastasis	ATRX	p.D2144G	FALSE	X	76814213	T	C	Missense_Mutation	0,366047745	377	TRUE	0.90	Clonal
P-0004026-T01-IM5	Pancreatic neuroendocrine tumor	Metastasis	MEN1	p.V101Rfs*16	FALSE	11	64577281	-	G	Frame_Shift_Ins	0,389770723	567	FALSE	1	Clonal
P-0004185-T02-IM6	Pancreatic neuroendocrine tumor	Metastasis	DAXX	p.N268S	FALSE	6	33288749	T	C	Missense_Mutation	0,743707094	437	TRUE	1	Clonal
P-0004185-T02-IM6	Pancreatic neuroendocrine tumor	Metastasis	ATRX	p.H2260R	FALSE	X	76778800	T	C	Missense_Mutation	0,724373576	439	TRUE	1	Clonal
P-0004185-T02-IM6	Pancreatic neuroendocrine tumor	Metastasis	MEN1	p.Y328Tfs*45	FALSE	11	64573786	A	-	Frame_Shift_Del	0,770061728	648	TRUE	1	Clonal
P-0004185-T02-IM6	Pancreatic neuroendocrine tumor	Metastasis	TSC2	p.X325_splice	FALSE	16	2108877	A	C	Splice_Site	0,782462057	593	TRUE	1	Clonal
P-0007076-T03-IM5	Pancreatic neuroendocrine tumor	Metastasis	ERRFI1	p.E413K	FALSE	1	8073422	C	T	Missense_Mutation	0,212284483	1856	FALSE	0.79	Subclonal
P-0007076-T03-IM5	Pancreatic neuroendocrine tumor	Metastasis	PIK3CD	p.G330S	FALSE	1	9777652	G	A	Missense_Mutation	0,054852321	948	FALSE	0.2	Subclonal
P-0007076-T03-IM5	Pancreatic neuroendocrine tumor	Metastasis	MTOR	p.W2304*	FALSE	1	11181324	C	T	Nonsense_Mutation	0,211940299	1005	FALSE	0.79	Subclonal
P-0007076-T03-IM5	Pancreatic neuroendocrine tumor	Metastasis	MTOR	p.L2261F	FALSE	1	11182065	G	A	Missense_Mutation	0,257836198	989	FALSE	0.96	Clonal
P-0007076-T03-IM5	Pancreatic neuroendocrine tumor	Metastasis	SPEN	p.E619K	FALSE	1	16254590	G	A	Missense_Mutation	0,075968992	1290	FALSE	0.28	Subclonal
P-0007076-T03-IM5	Pancreatic neuroendocrine tumor	Metastasis	SPEN	p.P3428S	FALSE	1	16263913	C	T	Missense_Mutation	0,206568712	1157	FALSE	0.77	Subclonal
P-0007076-T03-IM5	Pancreatic neuroendocrine tumor	Metastasis	ARID1A	p.A1304V	FALSE	1	27100115	C	T	Missense_Mutation	0,247551202	1123	FALSE	0.92	Clonal
P-0007076-T03-IM5	Pancreatic neuroendocrine tumor	Metastasis	CSF3R	p.G757S	FALSE	1	36932200	C	T	Missense_Mutation	0,240713224	1346	FALSE	0.89	Clonal
P-0007076-T03-IM5	Pancreatic neuroendocrine tumor	Metastasis	PIK3R3	p.E74K	FALSE	1	46543281	C	T	Missense_Mutation	0,178506375	1098	FALSE	0.66	Subclonal
P-0007076-T03-IM5	Pancreatic neuroendocrine tumor	Metastasis	JUN	p.Q321*	FALSE	1	59247782	G	A	Nonsense_Mutation	0,234673698	1517	FALSE	0.87	Clonal
P-0007076-T03-IM5	Pancreatic neuroendocrine tumor	Metastasis	JAK1	p.V409I	FALSE	1	65325897	C	T	Missense_Mutation	0,226824458	1014	FALSE	0.84	Clonal
P-0007076-T03-IM5	Pancreatic neuroendocrine tumor	Metastasis	NRAS	p.G13S	TRUE	1	115258745	C	T	Missense_Mutation	0,075604839	992	FALSE	0.28	Subclonal
P-0007076-T03-IM5	Pancreatic neuroendocrine tumor	Metastasis	VTCN1	p.X242_splice	FALSE	1	117695712	C	T	Splice_Site	0,196581197	1053	FALSE	0.73	Subclonal
P-0007076-T03-IM5	Pancreatic neuroendocrine tumor	Metastasis	MCL1	p.G59E	FALSE	1	150551831	C	T	Missense_Mutation	0,249056604	265	FALSE	0.92	Clonal
P-0007076-T03-IM5	Pancreatic neuroendocrine tumor	Metastasis	DDR2	p.E361K	FALSE	1	162731226	G	A	Missense_Mutation	0,069767442	946	FALSE	0.26	Subclonal
P-0007076-T03-IM5	Pancreatic neuroendocrine tumor	Metastasis	IKBKE	p.T516I	FALSE	1	206658574	C	T	Missense_Mutation	0,07204611	1041	FALSE	0.27	Subclonal
P-0007076-T03-IM5	Pancreatic neuroendocrine tumor	Metastasis	H3F3A	p.D82N	FALSE	1	226253472	G	A	Missense_Mutation	0,068143101	587	FALSE	0.25	Subclonal
P-0007076-T03-IM5	Pancreatic neuroendocrine tumor	Metastasis	FH	p.P177S	FALSE	1	241675293	G	A	Missense_Mutation	0,225498699	1153	FALSE	0.84	Clonal
P-0007076-T03-IM5	Pancreatic neuroendocrine tumor	Metastasis	MYCN	p.D272N	FALSE	2	16085638	G	A	Missense_Mutation	0,080931264	902	FALSE	0.3	Subclonal
P-0007076-T03-IM5	Pancreatic neuroendocrine tumor	Metastasis	DNMT3A	p.A791V	FALSE	2	25462035	G	A	Missense_Mutation	0,077696527	1094	FALSE	0.29	Subclonal
P-0007076-T03-IM5	Pancreatic neuroendocrine tumor	Metastasis	ASXL2	p.E239K	FALSE	2	25990512	C	T	Missense_Mutation	0,211659193	1115	FALSE	0.78	Subclonal
P-0007076-T03-IM5	Pancreatic neuroendocrine tumor	Metastasis	MSH2	p.E853K	FALSE	2	47707933	G	A	Missense_Mutation	0,224545455	1100	FALSE	0.83	Clonal
P-0007076-T03-IM5	Pancreatic neuroendocrine tumor	Metastasis	MSH6	p.D1346N	FALSE	2	48033952	G	A	Missense_Mutation	0,07881137	774	FALSE	0.29	Subclonal
P-0007076-T03-IM5	Pancreatic neuroendocrine tumor	Metastasis	XPO1	p.A377T	FALSE	2	61721145	C	T	Missense_Mutation	0,233421751	1131	FALSE	0.86	Clonal
P-0007076-T03-IM5	Pancreatic neuroendocrine tumor	Metastasis	ERCC3	p.D54N	FALSE	2	128051163	C	T	Missense_Mutation	0,058201058	1134	FALSE	0.22	Subclonal
P-0007076-T03-IM5	Pancreatic neuroendocrine tumor	Metastasis	ERBB4	p.V315I	FALSE	2	212578314	C	T	Missense_Mutation	0,058695652	920	FALSE	0.22	Subclonal
P-0007076-T03-IM5	Pancreatic neuroendocrine tumor	Metastasis	INHA	p.P162L	FALSE	2	220439632	C	T	Missense_Mutation	0,218253968	1260	FALSE	0.81	Subclonal
P-0007076-T03-IM5	Pancreatic neuroendocrine tumor	Metastasis	MLH1	p.A380V	FALSE	3	37067228	C	T	Missense_Mutation	0,080500894	1118	FALSE	0.3	Subclonal
P-0007076-T03-IM5	Pancreatic neuroendocrine tumor	Metastasis	MYD88	p.P83S	FALSE	3	38180399	C	T	Missense_Mutation	0,21107628	957	FALSE	0.78	Subclonal
P-0007076-T03-IM5	Pancreatic neuroendocrine tumor	Metastasis	SETD2	p.W1341*	FALSE	3	47162103	C	T	Nonsense_Mutation	0,066385669	949	FALSE	0.25	Subclonal
P-0007076-T03-IM5	Pancreatic neuroendocrine tumor	Metastasis	SETD2	p.Q1287*	FALSE	3	47162267	G	A	Nonsense_Mutation	0,066312997	1131	FALSE	0.25	Subclonal
P-0007076-T03-IM5	Pancreatic neuroendocrine tumor	Metastasis	RYBP	p.A222T	FALSE	3	72428228	C	T	Missense_Mutation	0,052422558	1259	FALSE	0.19	Subclonal
P-0007076-T03-IM5	Pancreatic neuroendocrine tumor	Metastasis	BCL6	p.E309K	FALSE	3	187447268	C	T	Missense_Mutation	0,207317073	820	FALSE	0.77	Subclonal
P-0007076-T03-IM5	Pancreatic neuroendocrine tumor	Metastasis	BCL6	p.A291V	FALSE	3	187447321	G	A	Missense_Mutation	0,220994475	724	FALSE	0.82	Clonal
P-0007076-T03-IM5	Pancreatic neuroendocrine tumor	Metastasis	TP63	p.D78N	FALSE	3	189456471	G	A	Missense_Mutation	0,050861362	1219	FALSE	0.19	Subclonal
P-0007076-T03-IM5	Pancreatic neuroendocrine tumor	Metastasis	KIT	p.S239F	FALSE	4	55565892	C	T	Missense_Mutation	0,135229251	1723	FALSE	0.77	Subclonal
P-0007076-T03-IM5	Pancreatic neuroendocrine tumor	Metastasis	KDR	p.X991_splice	FALSE	4	55958882	C	T	Splice_Site	0,144354839	1240	FALSE	0.82	Clonal
P-0007076-T03-IM5	Pancreatic neuroendocrine tumor	Metastasis	EPHA5	p.D902V	FALSE	4	66201797	T	A	Missense_Mutation	0,133896261	1658	FALSE	0.76	Subclonal
P-0007076-T03-IM5	Pancreatic neuroendocrine tumor	Metastasis	EPHA5	p.T246I	FALSE	4	66467532	G	A	Missense_Mutation	0,155944416	1943	FALSE	0.89	Clonal
P-0007076-T03-IM5	Pancreatic neuroendocrine tumor	Metastasis	TET2	p.E1401K	FALSE	4	106193739	G	A	Missense_Mutation	0,215547703	566	FALSE	0.8	Subclonal
P-0007076-T03-IM5	Pancreatic neuroendocrine tumor	Metastasis	TERT	p.P923S	FALSE	5	1264595	G	A	Missense_Mutation	0,052586938	1179	FALSE	0.19	Subclonal
P-0007076-T03-IM5	Pancreatic neuroendocrine tumor	Metastasis	TERT	p.P250L	FALSE	5	1294252	G	A	Missense_Mutation	0,061657033	519	FALSE	0.23	Subclonal
P-0007076-T03-IM5	Pancreatic neuroendocrine tumor	Metastasis	RICTOR	p.G356E	FALSE	5	38967523	C	T	Missense_Mutation	0,236180905	995	FALSE	0.88	Clonal
P-0007076-T03-IM5	Pancreatic neuroendocrine tumor	Metastasis	APC	p.R2565K	FALSE	5	112178985	G	A	Missense_Mutation	0,058020478	879	FALSE	0.22	Subclonal
P-0007076-T03-IM5	Pancreatic neuroendocrine tumor	Metastasis	RAD50	p.T773I	FALSE	5	131939102	C	T	Missense_Mutation	0,215646941	997	FALSE	0.8	Subclonal
P-0007076-T03-IM5	Pancreatic neuroendocrine tumor	Metastasis	CSF1R	p.G182D	FALSE	5	149459662	C	T	Missense_Mutation	0,242661448	1022	FALSE	0.9	Clonal
P-0007076-T03-IM5	Pancreatic neuroendocrine tumor	Metastasis	NSD1	p.V222I	FALSE	5	176562768	G	A	Missense_Mutation	0,071367884	1177	FALSE	0.26	Subclonal
P-0007076-T03-IM5	Pancreatic neuroendocrine tumor	Metastasis	NSD1	p.Q2313*	FALSE	5	176721306	C	T	Nonsense_Mutation	0,235294118	714	FALSE	0.87	Clonal
P-0007076-T03-IM5	Pancreatic neuroendocrine tumor	Metastasis	NOTCH4	p.R1862W	FALSE	6	32163642	G	A	Missense_Mutation	0,071186441	590	FALSE	0.26	Subclonal
P-0007076-T03-IM5	Pancreatic neuroendocrine tumor	Metastasis	NOTCH4	p.A947V	FALSE	6	32178554	G	A	Missense_Mutation	0,119648738	911	FALSE	0.44	Subclonal
P-0007076-T03-IM5	Pancreatic neuroendocrine tumor	Metastasis	NOTCH4	p.S557N	FALSE	6	32184998	C	T	Missense_Mutation	0,058666667	1125	FALSE	0.22	Subclonal
P-0007076-T03-IM5	Pancreatic neuroendocrine tumor	Metastasis	DAXX	p.F317S	FALSE	6	33288602	A	G	Missense_Mutation	0,603107345	708	TRUE	1	Clonal
P-0007076-T03-IM5	Pancreatic neuroendocrine tumor	Metastasis	PNRC1	p.S161N	FALSE	6	89791095	G	A	Missense_Mutation	0,107142857	112	FALSE	0.4	Subclonal
P-0007076-T03-IM5	Pancreatic neuroendocrine tumor	Metastasis	FYN	p.V324I	FALSE	6	112017543	C	T	Missense_Mutation	0,222977146	1619	FALSE	0.83	Clonal
P-0007076-T03-IM5	Pancreatic neuroendocrine tumor	Metastasis	TNFAIP3	p.P450S	FALSE	6	138199930	C	T	Missense_Mutation	0,11971831	568	FALSE	0.44	Subclonal
P-0007076-T03-IM5	Pancreatic neuroendocrine tumor	Metastasis	LATS1	p.P312S	FALSE	6	150005291	G	A	Missense_Mutation	0,071729958	1185	FALSE	0.27	Subclonal
P-0007076-T03-IM5	Pancreatic neuroendocrine tumor	Metastasis	LATS1	p.G195E	FALSE	6	150005641	C	T	Missense_Mutation	0,212598425	889	FALSE	0.79	Subclonal
P-0007076-T03-IM5	Pancreatic neuroendocrine tumor	Metastasis	LATS1	p.T126I	FALSE	6	150016329	G	A	Missense_Mutation	0,228501229	1221	FALSE	0.85	Clonal
P-0007076-T03-IM5	Pancreatic neuroendocrine tumor	Metastasis	ARID1B	p.P277S	FALSE	6	157099892	C	T	Missense_Mutation	0,058315335	926	FALSE	0.22	Subclonal
P-0007076-T03-IM5	Pancreatic neuroendocrine tumor	Metastasis	ARID1B	p.S645F	FALSE	6	157256607	C	T	Missense_Mutation	0,057013575	1105	FALSE	0.21	Subclonal
P-0007076-T03-IM5	Pancreatic neuroendocrine tumor	Metastasis	EGFR	p.S198N	FALSE	7	55219020	G	A	Missense_Mutation	0,153066967	1777	FALSE	0.87	Clonal
P-0007076-T03-IM5	Pancreatic neuroendocrine tumor	Metastasis	EGFR	p.W880*	FALSE	7	55260472	G	A	Nonsense_Mutation	0,143630103	2033	FALSE	0.82	Clonal
P-0007076-T03-IM5	Pancreatic neuroendocrine tumor	Metastasis	MET	p.L949F	FALSE	7	116411666	C	T	Missense_Mutation	0,157608696	1656	FALSE	0.9	Clonal
P-0007076-T03-IM5	Pancreatic neuroendocrine tumor	Metastasis	MET	p.P1283S	FALSE	7	116435757	C	T	Missense_Mutation	0,124721604	1796	FALSE	0.71	Subclonal
P-0007076-T03-IM5	Pancreatic neuroendocrine tumor	Metastasis	MLL3	p.P4110L	FALSE	7	151849987	G	A	Missense_Mutation	0,157152451	1489	FALSE	0.9	Clonal
P-0007076-T03-IM5	Pancreatic neuroendocrine tumor	Metastasis	MLL3	p.P4035S	FALSE	7	151851388	G	A	Missense_Mutation	0,058263971	1682	FALSE	0.33	Subclonal
P-0007076-T03-IM5	Pancreatic neuroendocrine tumor	Metastasis	MLL3	p.V4011I	FALSE	7	151851460	C	T	Missense_Mutation	0,097441481	1837	FALSE	0.56	Subclonal
P-0007076-T03-IM5	Pancreatic neuroendocrine tumor	Metastasis	FGFR1	p.D766N	FALSE	8	38271525	C	T	Missense_Mutation	0,078252033	984	FALSE	0.29	Subclonal
P-0007076-T03-IM5	Pancreatic neuroendocrine tumor	Metastasis	SOX17	p.D215N	FALSE	8	55371953	G	A	Missense_Mutation	0,256476684	386	FALSE	0.95	Clonal
P-0007076-T03-IM5	Pancreatic neuroendocrine tumor	Metastasis	RAD21	p.L451F	FALSE	8	117864306	G	A	Missense_Mutation	0,084938704	1142	FALSE	0.31	Subclonal

Table S2

Sample ID	Cancer subtype	Sample type	Symbol	Aminoacid change	Hotspot	Chromosome	Position	Reference	Alternate	Mutation type	Tumor MAF	Tumor DP	LOH	Cancer cell fraction	Clonal status
P-0007076-T03-IM5	Pancreatic neuroendocrine tumor	Metastasis	RAD21	p.R384K	FALSE	8	117866494	C	T	Missense_Mutation	0,082106894	1291	FALSE	0.3	Subclonal
P-0007076-T03-IM5	Pancreatic neuroendocrine tumor	Metastasis	JAK2	p.G139S	FALSE	9	5044467	G	A	Missense_Mutation	0,090304183	2104	FALSE	0.52	Subclonal
P-0007076-T03-IM5	Pancreatic neuroendocrine tumor	Metastasis	PTPRD	p.L871F	FALSE	9	8486206	G	A	Missense_Mutation	0,24159292	1130	FALSE	0.9	Clonal
P-0007076-T03-IM5	Pancreatic neuroendocrine tumor	Metastasis	GNAQ	p.R166C	FALSE	9	80412545	G	A	Missense_Mutation	0,206	1000	FALSE	0.76	Subclonal
P-0007076-T03-IM5	Pancreatic neuroendocrine tumor	Metastasis	SYK	p.Q145*	FALSE	9	93607731	C	T	Nonsense_Mutation	0,240135287	887	FALSE	0.89	Clonal
P-0007076-T03-IM5	Pancreatic neuroendocrine tumor	Metastasis	PPP6C	p.G211R	FALSE	9	127915850	C	T	Missense_Mutation	0,076135637	1563	FALSE	0.28	Subclonal
P-0007076-T03-IM5	Pancreatic neuroendocrine tumor	Metastasis	ABL1	p.V33I	FALSE	9	133729468	G	A	Missense_Mutation	0,21906924	881	FALSE	0.81	Subclonal
P-0007076-T03-IM5	Pancreatic neuroendocrine tumor	Metastasis	ABL1	p.S977N	FALSE	9	133760607	G	A	Missense_Mutation	0,238720263	1219	FALSE	0.88	Clonal
P-0007076-T03-IM5	Pancreatic neuroendocrine tumor	Metastasis	TSC1	p.P391S	FALSE	9	135786050	G	A	Missense_Mutation	0,231632653	980	FALSE	0.86	Clonal
P-0007076-T03-IM5	Pancreatic neuroendocrine tumor	Metastasis	NOTCH1	p.L2139F	FALSE	9	139391776	G	A	Missense_Mutation	0,061032864	639	FALSE	0.23	Subclonal
P-0007076-T03-IM5	Pancreatic neuroendocrine tumor	Metastasis	NOTCH1	p.X481_splice	FALSE	9	139411838	C	T	Splice_Site	0,237696335	955	FALSE	0.88	Clonal
P-0007076-T03-IM5	Pancreatic neuroendocrine tumor	Metastasis	TRAF2	p.G193D	FALSE	9	139804421	G	A	Missense_Mutation	0,2074883	1282	FALSE	0.77	Subclonal
P-0007076-T03-IM5	Pancreatic neuroendocrine tumor	Metastasis	ARID5B	p.P182S	FALSE	10	63759891	C	T	Missense_Mutation	0,108849558	1130	FALSE	0.4	Subclonal
P-0007076-T03-IM5	Pancreatic neuroendocrine tumor	Metastasis	TET1	p.R938K	FALSE	10	70405299	G	A	Missense_Mutation	0,058876812	1104	FALSE	0.22	Subclonal
P-0007076-T03-IM5	Pancreatic neuroendocrine tumor	Metastasis	TET1	p.G1121D	FALSE	10	70405848	G	A	Missense_Mutation	0,214016579	1327	FALSE	0.79	Subclonal
P-0007076-T03-IM5	Pancreatic neuroendocrine tumor	Metastasis	TET1	p.G1458D	FALSE	10	70412263	G	A	Missense_Mutation	0,056696795	1217	FALSE	0.21	Subclonal
P-0007076-T03-IM5	Pancreatic neuroendocrine tumor	Metastasis	PTEN	p.S59*	FALSE	10	89685281	C	A	Nonsense_Mutation	0,545918367	588	TRUE	1	Clonal
P-0007076-T03-IM5	Pancreatic neuroendocrine tumor	Metastasis	FGFR2	p.D321N	FALSE	10	123276956	C	T	Missense_Mutation	0,08577633	921	FALSE	0.32	Subclonal
P-0007076-T03-IM5	Pancreatic neuroendocrine tumor	Metastasis	MEN1	p.R465*	FALSE	11	64572261	G	A	Nonsense_Mutation	0,588289113	1093	TRUE	1	Clonal
P-0007076-T03-IM5	Pancreatic neuroendocrine tumor	Metastasis	EED	p.P203L	FALSE	11	85968612	C	T	Missense_Mutation	0,060220526	1179	FALSE	0.22	Subclonal
P-0007076-T03-IM5	Pancreatic neuroendocrine tumor	Metastasis	YAP1	p.E459K	FALSE	11	102100531	G	A	Missense_Mutation	0,234775641	1248	FALSE	0.87	Clonal
P-0007076-T03-IM5	Pancreatic neuroendocrine tumor	Metastasis	ATM	p.S2489F	FALSE	11	108201099	C	T	Missense_Mutation	0,211235955	890	TRUE	0.78	Subclonal
P-0007076-T03-IM5	Pancreatic neuroendocrine tumor	Metastasis	MLL	p.L499F	FALSE	11	118343369	C	T	Missense_Mutation	0,087389381	904	FALSE	0.32	Subclonal
P-0007076-T03-IM5	Pancreatic neuroendocrine tumor	Metastasis	MLL	p.V916I	FALSE	11	118344620	G	A	Missense_Mutation	0,243465273	1339	FALSE	0.9	Clonal
P-0007076-T03-IM5	Pancreatic neuroendocrine tumor	Metastasis	MLL	p.G1160D	FALSE	11	118348826	G	A	Missense_Mutation	0,071244635	1165	FALSE	0.26	Subclonal
P-0007076-T03-IM5	Pancreatic neuroendocrine tumor	Metastasis	CBL	p.X812_splice	FALSE	11	119170204	G	A	Splice_Site	0,06884058	828	FALSE	0.26	Subclonal
P-0007076-T03-IM5	Pancreatic neuroendocrine tumor	Metastasis	KDM5A	p.G129S	FALSE	12	475252	C	T	Missense_Mutation	0,13878327	1578	FALSE	0.79	Subclonal
P-0007076-T03-IM5	Pancreatic neuroendocrine tumor	Metastasis	KDM5A	p.W98*	FALSE	12	493269	C	T	Nonsense_Mutation	0,143280632	2024	FALSE	0.82	Clonal
P-0007076-T03-IM5	Pancreatic neuroendocrine tumor	Metastasis	PIK3C2G	p.Q326*	FALSE	12	18446891	C	T	Nonsense_Mutation	0,128706625	1585	FALSE	0.73	Subclonal
P-0007076-T03-IM5	Pancreatic neuroendocrine tumor	Metastasis	PIK3C2G	p.S1196F	FALSE	12	18715756	C	T	Missense_Mutation	0,133410673	1724	FALSE	0.76	Subclonal
P-0007076-T03-IM5	Pancreatic neuroendocrine tumor	Metastasis	KRAS	p.E63K	TRUE	12	25380271	C	T	Missense_Mutation	0,12716175	1966	FALSE	0.73	Subclonal
P-0007076-T03-IM5	Pancreatic neuroendocrine tumor	Metastasis	ARID2	p.P1518S	FALSE	12	46246458	C	T	Missense_Mutation	0,1221174	1908	FALSE	0.7	Subclonal
P-0007076-T03-IM5	Pancreatic neuroendocrine tumor	Metastasis	ARID2	p.P1621S	FALSE	12	46254671	C	T	Missense_Mutation	0,055714286	2100	FALSE	0.32	Subclonal
P-0007076-T03-IM5	Pancreatic neuroendocrine tumor	Metastasis	ARID2	p.E1817K	FALSE	12	46298802	G	A	Missense_Mutation	0,136624569	1742	FALSE	0.78	Subclonal
P-0007076-T03-IM5	Pancreatic neuroendocrine tumor	Metastasis	HNF1A	p.A501T	FALSE	12	121435468	G	A	Missense_Mutation	0,057615894	1510	FALSE	0.33	Subclonal
P-0007076-T03-IM5	Pancreatic neuroendocrine tumor	Metastasis	POLE	p.G996S	FALSE	12	133237629	C	T	Missense_Mutation	0,05625374	1671	FALSE	0.32	Subclonal
P-0007076-T03-IM5	Pancreatic neuroendocrine tumor	Metastasis	POLE	p.W669*	FALSE	12	133245240	C	T	Nonsense_Mutation	0,103667137	1418	FALSE	0.59	Subclonal
P-0007076-T03-IM5	Pancreatic neuroendocrine tumor	Metastasis	LATS2	p.P201S	FALSE	13	21563318	G	A	Missense_Mutation	0,14258794	1592	FALSE	0.81	Subclonal
P-0007076-T03-IM5	Pancreatic neuroendocrine tumor	Metastasis	BRCA2	p.E1695K	FALSE	13	32913575	G	A	Missense_Mutation	0,09369287	2188	FALSE	0.53	Subclonal
P-0007076-T03-IM5	Pancreatic neuroendocrine tumor	Metastasis	FOXO1	p.S287N	FALSE	13	41134768	C	T	Missense_Mutation	0,147970319	2291	FALSE	0.84	Clonal
P-0007076-T03-IM5	Pancreatic neuroendocrine tumor	Metastasis	IRS2	p.A1096V	FALSE	13	110435114	G	A	Missense_Mutation	0,050865801	924	FALSE	0.29	Subclonal
P-0007076-T03-IM5	Pancreatic neuroendocrine tumor	Metastasis	IRS2	p.P877S	FALSE	13	110435772	G	A	Missense_Mutation	0,172161172	273	FALSE	0.98	Clonal
P-0007076-T03-IM5	Pancreatic neuroendocrine tumor	Metastasis	NKX2-1	p.X155_splice	FALSE	14	36987226	C	T	Splice_Site	0,131805158	698	FALSE	0.75	Subclonal
P-0007076-T03-IM5	Pancreatic neuroendocrine tumor	Metastasis	FOXA1	p.G281E	FALSE	14	38061147	C	T	Missense_Mutation	0,056497175	354	FALSE	0.32	Subclonal
P-0007076-T03-IM5	Pancreatic neuroendocrine tumor	Metastasis	MGA	p.P2131S	FALSE	15	42042196	C	T	Missense_Mutation	0,222222222	1575	FALSE	0.82	Clonal
P-0007076-T03-IM5	Pancreatic neuroendocrine tumor	Metastasis	SMAD3	p.G17D	FALSE	15	67358542	G	A	Missense_Mutation	0,052690583	892	FALSE	0.2	Subclonal
P-0007076-T03-IM5	Pancreatic neuroendocrine tumor	Metastasis	CD276	p.A312T	FALSE	15	73996200	G	A	Missense_Mutation	0,233333333	660	FALSE	0.86	Clonal
P-0007076-T03-IM5	Pancreatic neuroendocrine tumor	Metastasis	BLM	p.T999I	FALSE	15	91334051	C	T	Missense_Mutation	0,050108932	918	FALSE	0.19	Subclonal
P-0007076-T03-IM5	Pancreatic neuroendocrine tumor	Metastasis	IGF1R	p.P262S	FALSE	15	99434697	C	T	Missense_Mutation	0,258097166	988	FALSE	0.96	Clonal
P-0007076-T03-IM5	Pancreatic neuroendocrine tumor	Metastasis	IGF1R	p.L910F	FALSE	15	99467859	C	T	Missense_Mutation	0,065236819	1119	FALSE	0.24	Subclonal
P-0007076-T03-IM5	Pancreatic neuroendocrine tumor	Metastasis	AXIN1	p.X821_splice	FALSE	16	339439	C	T	Splice_Site	0,123913043	920	FALSE	0.46	Subclonal
P-0007076-T03-IM5	Pancreatic neuroendocrine tumor	Metastasis	TSC2	p.E234*	FALSE	16	2106696	G	T	Nonsense_Mutation	0,59551495	1204	TRUE	1	Clonal
P-0007076-T03-IM5	Pancreatic neuroendocrine tumor	Metastasis	TRAF7	p.S652F	FALSE	16	2226342	C	T	Missense_Mutation	0,076624636	1031	FALSE	0.28	Subclonal
P-0007076-T03-IM5	Pancreatic neuroendocrine tumor	Metastasis	PDPK1	p.W500*	FALSE	16	2647222	G	A	Nonsense_Mutation	0,108894431	1203	FALSE	0.4	Subclonal
P-0007076-T03-IM5	Pancreatic neuroendocrine tumor	Metastasis	CREBBP	p.P1997L	FALSE	16	3779058	G	A	Missense_Mutation	0,062318841	690	TRUE	0.23	Subclonal
P-0007076-T03-IM5	Pancreatic neuroendocrine tumor	Metastasis	CREBBP	p.P1951L	FALSE	16	3779196	G	A	Missense_Mutation	0,190283401	494	TRUE	0.71	Subclonal
P-0007076-T03-IM5	Pancreatic neuroendocrine tumor	Metastasis	GRIN2A	p.S81N	FALSE	16	10274027	C	T	Missense_Mutation	0,052325581	1032	FALSE	0.19	Subclonal
P-0007076-T03-IM5	Pancreatic neuroendocrine tumor	Metastasis	CTCF	p.H485Y	FALSE	16	67660553	C	T	Missense_Mutation	0,065046462	1399	FALSE	0.24	Subclonal
P-0007076-T03-IM5	Pancreatic neuroendocrine tumor	Metastasis	ZFHX3	p.P3675S	FALSE	16	72821152	G	A	Missense_Mutation	0,059167275	1369	FALSE	0.22	Subclonal
P-0007076-T03-IM5	Pancreatic neuroendocrine tumor	Metastasis	ZFHX3	p.P3212S	FALSE	16	72822541	G	A	Missense_Mutation	0,059931507	1168	FALSE	0.22	Subclonal
P-0007076-T03-IM5	Pancreatic neuroendocrine tumor	Metastasis	ZFHX3	p.A438V	FALSE	16	72992732	G	A	Missense_Mutation	0,217178771	1432	FALSE	0.8	Subclonal
P-0007076-T03-IM5	Pancreatic neuroendocrine tumor	Metastasis	ANKRD11	p.H1598Y	FALSE	16	89348158	G	A	Missense_Mutation	0,059884095	1553	FALSE	0.22	Subclonal
P-0007076-T03-IM5	Pancreatic neuroendocrine tumor	Metastasis	ANKRD11	p.E53K	FALSE	16	89371683	C	T	Missense_Mutation	0,076095311	1301	FALSE	0.28	Subclonal
P-0007076-T03-IM5	Pancreatic neuroendocrine tumor	Metastasis	TP53	p.R175H	TRUE	17	7578406	C	T	Missense_Mutation	0,035040431	742	TRUE	0.13	Subclonal
P-0007076-T03-IM5	Pancreatic neuroendocrine tumor	Metastasis	NCOR1	p.G1311D	FALSE	17	15974943	C	T	Missense_Mutation	0,138919515	1814	FALSE	0.79	Subclonal
P-0007076-T03-IM5	Pancreatic neuroendocrine tumor	Metastasis	CDK12	p.T737I	FALSE	17	37649105	C	T	Missense_Mutation	0,148474178	1704	FALSE	0.85	Clonal
P-0007076-T03-IM5	Pancreatic neuroendocrine tumor	Metastasis	CDK12	p.G1284D	FALSE	17	37686947	G	A	Missense_Mutation	0,060697115	1664	FALSE	0.35	Subclonal
P-0007076-T03-IM5	Pancreatic neuroendocrine tumor	Metastasis	ERBB2	p.T328I	FALSE	17	37868262	C	T	Missense_Mutation	0,133412746	1679	FALSE	0.76	Subclonal
P-0007076-T03-IM5	Pancreatic neuroendocrine tumor	Metastasis	ERBB2	p.X990_splice	FALSE	17	37882913	G	A	Splice_Site	0,141796084	1481	FALSE	0.81	Subclonal
P-0007076-T03-IM5	Pancreatic neuroendocrine tumor	Metastasis	ERBB2	p.V1128I	FALSE	17	37883770	G	A	Missense_Mutation	0,149316509	1902	FALSE	0.85	Clonal
P-0007076-T03-IM5	Pancreatic neuroendocrine tumor	Metastasis	STAT5B	p.S722N	FALSE	17	40354430	C	T	Missense_Mutation	0,157590759	2424	FALSE	0.9	Clonal
P-0007076-T03-IM5	Pancreatic neuroendocrine tumor	Metastasis	SPOP	p.V161M	FALSE	17	47688819	C	T	Missense_Mutation	0,064722617	1406	FALSE	0.37	Subclonal
P-0007076-T03-IM5	Pancreatic neuroendocrine tumor	Metastasis	AXIN2	p.E563K	FALSE	17	63533467	C	T	Missense_Mutation	0,141562064	1434	FALSE	0.81	Subclonal
P-0007076-T03-IM5	Pancreatic neuroendocrine tumor	Metastasis	SRSF2	p.G12D	FALSE	17	74733208	C	T	Missense_Mutation	0,05398773	1630	FALSE	0.31	Subclonal
P-0007076-T03-IM5	Pancreatic neuroendocrine tumor	Metastasis	PIK3C3	p.E263K	FALSE	18	39575854	G	A	Missense_Mutation	0,241421569	816	FALSE	0.89	Clonal
P-0007076-T03-IM5	Pancreatic neuroendocrine tumor	Metastasis	DOT1L	p.A500T	FALSE	19	2211782	G	A	Missense_Mutation	0,077094144	1349	FALSE	0.29	Subclonal
P-0007076-T03-IM5	Pancreatic neuroendocrine tumor	Metastasis	DNMT1	p.G1402S	FALSE	19	10248549	C	T	Missense_Mutation	0,238779174	1114	FALSE	0.88	Clonal
P-0007076-T03-IM5	Pancreatic neuroendocrine tumor	Metastasis	NOTCH3	p.G1105D	FALSE	19	15290896	C	T	Missense_Mutation	0,132003799	2106	FALSE	0.75	Subclonal
P-0007076-T03-IM5	Pancreatic neuroendocrine tumor	Metastasis	BRD4	p.X404_splice	FALSE	19	15375214	C	T	Splice_Site	0,153996101	1026	FALSE	0.88	Clonal
P-0007076-T03-IM5	Pancreatic neuroendocrine tumor	Metastasis	JAK3	p.G987D	FALSE	19	17942055	C	T	Missense_Mutation	0,14265928	1444	FALSE	0.81	Subclonal
P-0007076-T03-IM5	Pancreatic neuroendocrine tumor	Metastasis	JAK3	p.V372M	FALSE	19	17952226	C	T	Missense_Mutation	0,140062598	1278	FALSE	0.8	Subclonal

Table S2

Sample ID	Cancer subtype	Sample type	Symbol	Aminoacid change	Hotspot	Chromosome	Position	Reference	Alternate	Mutation type	Tumor MAF	Tumor DP	LOH	Cancer cell fraction	Clonal status
P-0007076-T03-IM5	Pancreatic neuroendocrine tumor	Metastasis	MEF2B	p.T204M	FALSE	19	19257615	G	A	Missense_Mutation	0,142682927	1640	FALSE	0.81	Subclonal
P-0007076-T03-IM5	Pancreatic neuroendocrine tumor	Metastasis	SRC	p.R362W	FALSE	20	36030049	C	T	Missense_Mutation	0,067076923	1625	FALSE	0.38	Subclonal
P-0007076-T03-IM5	Pancreatic neuroendocrine tumor	Metastasis	GNAS	p.X324_splice	FALSE	20	57485137	G	A	Splice_Site	0,071316614	1276	FALSE	0.41	Subclonal
P-0007076-T03-IM5	Pancreatic neuroendocrine tumor	Metastasis	EP300	p.A13T	FALSE	22	41489045	G	A	Missense_Mutation	0,238556338	1136	FALSE	0.88	Clonal
P-0007076-T03-IM5	Pancreatic neuroendocrine tumor	Metastasis	EP300	p.A598T	FALSE	22	41536175	G	A	Missense_Mutation	0,207381371	1138	FALSE	0.77	Subclonal
P-0007076-T03-IM5	Pancreatic neuroendocrine tumor	Metastasis	EP300	p.P2107L	FALSE	22	41574035	C	T	Missense_Mutation	0,240559441	715	FALSE	0.89	Clonal
P-0007076-T03-IM5	Pancreatic neuroendocrine tumor	Metastasis	KDM6A	p.S718N	FALSE	X	44929053	G	A	Missense_Mutation	0,136904762	1512	FALSE	0.75	Subclonal
P-0007076-T03-IM5	Pancreatic neuroendocrine tumor	Metastasis	RBM10	p.Q664*	FALSE	X	47044898	C	T	Nonsense_Mutation	0,196531792	1038	FALSE	0.75	Subclonal
P-0007076-T03-IM5	Pancreatic neuroendocrine tumor	Metastasis	AMER1	p.D1050N	FALSE	X	63410019	C	T	Missense_Mutation	0,20749782	1147	FALSE	0.77	Subclonal
P-0007076-T03-IM5	Pancreatic neuroendocrine tumor	Metastasis	MED12	p.G1676D	FALSE	X	70356132	G	A	Missense_Mutation	0,064111498	1435	FALSE	0.24	Subclonal
P-0007076-T03-IM5	Pancreatic neuroendocrine tumor	Metastasis	MED12	p.P1978S	FALSE	X	70357681	C	T	Missense_Mutation	0,060216509	1478	FALSE	0.24	Subclonal
P-0007076-T03-IM5	Pancreatic neuroendocrine tumor	Metastasis	ATRX	p.S210N	FALSE	X	76940464	C	T	Missense_Mutation	0,063772049	1474	FALSE	0.24	Subclonal
P-0007076-T03-IM5	Pancreatic neuroendocrine tumor	Metastasis	MCL1	p.E171del	FALSE	1	150551492	TCC	-	In_Frame_Del	0,537272727	1100	FALSE	1	Clonal
P-0007076-T03-IM5	Pancreatic neuroendocrine tumor	Metastasis	IFNGR1	p.S378Ffs*6	FALSE	6	137519505	CT	-	Frame_Shift_Del	0,233	1000	FALSE	0.86	Clonal
P-0007076-T03-IM5	Pancreatic neuroendocrine tumor	Metastasis	CDKN1B	p.W60_K68delins*	FALSE	12	12870952	GGAATTTCGATTTTCAGAATCACA	-	Nonsense_Mutation	0,318914956	1364	FALSE	1	Clonal
P-0007076-T03-IM5	Pancreatic neuroendocrine tumor	Metastasis	CREBBP	p.M784Ifs*17	FALSE	16	3823845	CTGAGCGGGCGCCTGGGCC	-	Frame_Shift_Del	0,592964824	995	TRUE	1	Clonal
P-0007076-T03-IM5	Pancreatic neuroendocrine tumor	Metastasis	PTCH1	p.R1308Efs*64	FALSE	9	98209617	G	-	Frame_Shift_Del	0,065677966	472	FALSE	0.24	Subclonal
P-0010496-T02-IM6	Pancreatic neuroendocrine tumor	Metastasis	ATRX	p.N1321Kfs*3	FALSE	X	76919028	-	T	Frame_Shift_Ins	0,54308094	383	TRUE	1	Clonal
P-0010830-T01-IM5	Pancreatic neuroendocrine tumor	Metastasis	SH2B3	p.I568T	FALSE	12	111886081	T	C	Missense_Mutation	0,376884422	995	FALSE	0.98	Clonal
P-0010830-T01-IM5	Pancreatic neuroendocrine tumor	Metastasis	CREBBP	p.Q203*	FALSE	16	3900489	G	A	Nonsense_Mutation	0,677130045	446	TRUE	1	Clonal
P-0010830-T01-IM5	Pancreatic neuroendocrine tumor	Metastasis	ATRX	p.D2052V	FALSE	X	76845366	T	A	Missense_Mutation	0,643636364	275	TRUE	1	Clonal
P-0010830-T01-IM5	Pancreatic neuroendocrine tumor	Metastasis	MEN1	p.M239Gfs*14	FALSE	11	64575106	AT	-	Frame_Shift_Del	0,663461538	416	TRUE	1	Clonal
P-0013399-T01-IM5	Pancreatic neuroendocrine tumor	Metastasis	MEN1	p.I85Sfs*33	FALSE	11	64577330	AGAC	-	Frame_Shift_Del	0,630372493	349	TRUE	0.98	Clonal
P-0013399-T01-IM5	Pancreatic neuroendocrine tumor	Metastasis	BCL6	p.D437Y	FALSE	3	187446884	C	A	Missense_Mutation	0,660377358	265	FALSE	1	Clonal
P-0013399-T01-IM5	Pancreatic neuroendocrine tumor	Metastasis	CDKN1A	p.C41*	FALSE	6	36652001	C	A	Nonsense_Mutation	0,699228792	389	TRUE	1	Clonal
P-0013399-T01-IM5	Pancreatic neuroendocrine tumor	Metastasis	BRAF	p.T599K	FALSE	7	140453139	G	T	Missense_Mutation	0,398198198	555	FALSE	1	Clonal
P-0013399-T01-IM5	Pancreatic neuroendocrine tumor	Metastasis	TSC2	p.E481*	FALSE	16	2113052	G	T	Nonsense_Mutation	0,664082687	387	TRUE	1	Clonal
P-0013399-T01-IM5	Pancreatic neuroendocrine tumor	Metastasis	GNAS	p.K100E	FALSE	20	57478626	A	G	Missense_Mutation	0,437417655	759	FALSE	1	Clonal
P-0013399-T01-IM5	Pancreatic neuroendocrine tumor	Metastasis	ATRX	p.E492*	FALSE	X	76939274	C	A	Nonsense_Mutation	0,827433628	226	TRUE	1	Clonal
P-0013749-T02-IM6	Pancreatic neuroendocrine tumor	Metastasis	TSC1	p.D47N	FALSE	9	135802659	C	T	Missense_Mutation	0,05	740	FALSE	0.28	Subclonal
P-0013749-T02-IM6	Pancreatic neuroendocrine tumor	Metastasis	TP53	p.R248Q	TRUE	17	7577538	C	T	Missense_Mutation	0,080184332	1085	FALSE	0.46	Subclonal
P-0013749-T02-IM6	Pancreatic neuroendocrine tumor	Metastasis	TP53	p.R175H	TRUE	17	7578406	C	T	Missense_Mutation	0,144	1000	FALSE	0.79	Subclonal
P-0013749-T02-IM6	Pancreatic neuroendocrine tumor	Metastasis	PTEN	p.R130*	FALSE	10	89692904	C	T	Nonsense_Mutation	0,138983051	885	FALSE	0.75	Subclonal
P-0013749-T02-IM6	Pancreatic neuroendocrine tumor	Metastasis	FAT1	p.P3809L	FALSE	4	187524113	G	A	Missense_Mutation	0,056657224	1059	FALSE	0.35	Subclonal
P-0013749-T02-IM6	Pancreatic neuroendocrine tumor	Metastasis	MYOD1	p.A174T	FALSE	11	17741849	G	A	Missense_Mutation	0,080428954	373	FALSE	0.35	Subclonal
P-0013749-T02-IM6	Pancreatic neuroendocrine tumor	Metastasis	HNF1A	p.G181E	FALSE	12	121431338	G	A	Missense_Mutation	0,058551618	649	FALSE	0.28	Subclonal
P-0013749-T02-IM6	Pancreatic neuroendocrine tumor	Metastasis	SMAD3	p.G73D	FALSE	15	67457244	G	A	Missense_Mutation	0,056372549	816	FALSE	0.26	Subclonal
P-0013749-T02-IM6	Pancreatic neuroendocrine tumor	Metastasis	NOTCH3	p.G2112D	FALSE	19	15272104	C	T	Missense_Mutation	0,050164474	1216	FALSE	0.33	Subclonal
P-0013749-T02-IM6	Pancreatic neuroendocrine tumor	Metastasis	ERG	p.P275S	FALSE	21	39763629	G	A	Missense_Mutation	0,062080537	596	FALSE	0.29	Subclonal
P-0013749-T02-IM6	Pancreatic neuroendocrine tumor	Metastasis	BRD4	p.P311S	FALSE	19	15375496	G	A	Missense_Mutation	0,052345343	1471	FALSE	0.35	Subclonal
P-0013749-T02-IM6	Pancreatic neuroendocrine tumor	Metastasis	ANKRD11	p.G1194E	FALSE	16	89349369	C	T	Missense_Mutation	0,058252427	1133	FALSE	0.28	Subclonal
P-0013749-T02-IM6	Pancreatic neuroendocrine tumor	Metastasis	ERBB3	p.D581N	FALSE	12	56488222	G	A	Missense_Mutation	0,05	980	FALSE	0.35	Subclonal
P-0013749-T02-IM6	Pancreatic neuroendocrine tumor	Metastasis	MAP3K13	p.G50E	FALSE	3	185146518	G	A	Missense_Mutation	0,058823529	782	FALSE	0.28	Subclonal
P-0013749-T02-IM6	Pancreatic neuroendocrine tumor	Metastasis	DAXX	p.F613Lfs*31	FALSE	6	33287255	ATTG	-	Frame_Shift_Del	0,14797136	838	FALSE	0.78	Subclonal
P-0013749-T02-IM6	Pancreatic neuroendocrine tumor	Metastasis	ATRX	p.M118I	FALSE	X	76952081	C	T	Missense_Mutation	0,061776062	777	FALSE	0.33	Subclonal
P-0013749-T02-IM6	Pancreatic neuroendocrine tumor	Metastasis	ERRFI1	p.D405N	FALSE	1	8073446	C	T	Missense_Mutation	0,056010929	732	FALSE	0.29	Subclonal
P-0013749-T02-IM6	Pancreatic neuroendocrine tumor	Metastasis	ARID1A	p.G1617D	FALSE	1	27101568	G	A	Missense_Mutation	0,055508113	1171	FALSE	0.35	Subclonal
P-0013749-T02-IM6	Pancreatic neuroendocrine tumor	Metastasis	ARID1A	p.A1932T	FALSE	1	27106183	G	A	Missense_Mutation	0,064479638	884	FALSE	0.28	Subclonal
P-0013749-T02-IM6	Pancreatic neuroendocrine tumor	Metastasis	ARID1A	p.A2097T	FALSE	1	27106678	G	A	Missense_Mutation	0,076581576	901	FALSE	0.35	Subclonal
P-0013749-T02-IM6	Pancreatic neuroendocrine tumor	Metastasis	SESN2	p.V453I	FALSE	1	28607227	G	A	Missense_Mutation	0,068627451	714	FALSE	0.28	Subclonal
P-0013749-T02-IM6	Pancreatic neuroendocrine tumor	Metastasis	MYCL1	p.A272V	FALSE	1	40363414	G	A	Missense_Mutation	0,059811123	953	FALSE	0.26	Subclonal
P-0013749-T02-IM6	Pancreatic neuroendocrine tumor	Metastasis	PIK3R3	p.P127L	FALSE	1	46532698	G	A	Missense_Mutation	0,074820144	695	FALSE	0.33	Subclonal
P-0013749-T02-IM6	Pancreatic neuroendocrine tumor	Metastasis	CDKN2C	p.C141Y	FALSE	1	51439857	G	A	Missense_Mutation	0,059654631	637	FALSE	0.29	Subclonal
P-0013749-T02-IM6	Pancreatic neuroendocrine tumor	Metastasis	NEGR1	p.P311S	FALSE	1	72058509	G	A	Missense_Mutation	0,068676717	597	FALSE	0.35	Subclonal
P-0013749-T02-IM6	Pancreatic neuroendocrine tumor	Metastasis	FUBP1	p.R284K	FALSE	1	78430028	C	T	Missense_Mutation	0,072380952	525	FALSE	0.35	Subclonal
P-0013749-T02-IM6	Pancreatic neuroendocrine tumor	Metastasis	FAM46C	p.E136K	FALSE	1	118165896	G	A	Missense_Mutation	0,078152753	563	FALSE	0.35	Subclonal
P-0013749-T02-IM6	Pancreatic neuroendocrine tumor	Metastasis	DDR2	p.D154N	FALSE	1	162724988	G	A	Missense_Mutation	0,062750334	749	FALSE	0.28	Subclonal
P-0013749-T02-IM6	Pancreatic neuroendocrine tumor	Metastasis	CDC73	p.G49D	FALSE	1	193094256	G	A	Missense_Mutation	0,053191489	752	FALSE	0.35	Subclonal
P-0013749-T02-IM6	Pancreatic neuroendocrine tumor	Metastasis	IKBKE	p.A245V	FALSE	1	206651124	C	T	Missense_Mutation	0,051639344	1220	FALSE	0.28	Subclonal
P-0013749-T02-IM6	Pancreatic neuroendocrine tumor	Metastasis	SMYD3	p.D114N	FALSE	1	246493836	C	T	Missense_Mutation	0,055408971	758	FALSE	0.26	Subclonal
P-0013749-T02-IM6	Pancreatic neuroendocrine tumor	Metastasis	PTEN	p.R55K	FALSE	10	89653866	G	A	Missense_Mutation	0,055793991	699	FALSE	0.33	Subclonal
P-0013749-T02-IM6	Pancreatic neuroendocrine tumor	Metastasis	SUFU	p.G33E	FALSE	10	104264007	G	A	Missense_Mutation	0,05	1040	FALSE	0.29	Subclonal
P-0013749-T02-IM6	Pancreatic neuroendocrine tumor	Metastasis	TCF7L2	p.A543T	FALSE	10	114925549	G	A	Missense_Mutation	0,062032086	935	FALSE	0.35	Subclonal
P-0013749-T02-IM6	Pancreatic neuroendocrine tumor	Metastasis	MYOD1	p.P267L	FALSE	11	17742892	C	T	Missense_Mutation	0,066815145	898	FALSE	0.28	Subclonal
P-0013749-T02-IM6	Pancreatic neuroendocrine tumor	Metastasis	WT1	p.P110S	FALSE	11	32456564	G	A	Missense_Mutation	0,077181208	298	FALSE	0.35	Subclonal
P-0013749-T02-IM6	Pancreatic neuroendocrine tumor	Metastasis	YAP1	p.A239V	FALSE	11	102056776	C	T	Missense_Mutation	0,059202059	777	FALSE	0.28	Subclonal
P-0013749-T02-IM6	Pancreatic neuroendocrine tumor	Metastasis	ATM	p.A1211T	FALSE	11	108153491	G	A	Missense_Mutation	0,055485498	793	FALSE	0.26	Subclonal
P-0013749-T02-IM6	Pancreatic neuroendocrine tumor	Metastasis	ATM	p.N1377K	FALSE	11	108159725	T	A	Missense_Mutation	0,054640069	1153	FALSE	0.33	Subclonal
P-0013749-T02-IM6	Pancreatic neuroendocrine tumor	Metastasis	CBL	p.P809L	FALSE	11	119169242	C	T	Missense_Mutation	0,057863501	674	FALSE	0.29	Subclonal
P-0013749-T02-IM6	Pancreatic neuroendocrine tumor	Metastasis	KDM5A	p.R1222K	FALSE	12	416885	C	T	Missense_Mutation	0,053314121	694	FALSE	0.35	Subclonal
P-0013749-T02-IM6	Pancreatic neuroendocrine tumor	Metastasis	PIK3C2G	p.E13K	FALSE	12	18435052	G	A	Missense_Mutation	0,132231405	726	FALSE	0.75	Subclonal
P-0013749-T02-IM6	Pancreatic neuroendocrine tumor	Metastasis	MLL2	p.A2357T	FALSE	12	49434484	C	T	Missense_Mutation	0,05387931	1392	FALSE	0.28	Subclonal
P-0013749-T02-IM6	Pancreatic neuroendocrine tumor	Metastasis	LATS2	p.V762I	FALSE	13	21557561	C	T	Missense_Mutation	0,052631579	1083	FALSE	0.35	Subclonal
P-0013749-T02-IM6	Pancreatic neuroendocrine tumor	Metastasis	FLT1	p.V9I	FALSE	13	29068956	C	T	Missense_Mutation	0,050670641	671	FALSE	0.28	Subclonal
P-0013749-T02-IM6	Pancreatic neuroendocrine tumor	Metastasis	BRCA2	p.G2816S	FALSE	13	32944653	G	A	Missense_Mutation	0,05399061	852	FALSE	0.26	Subclonal
P-0013749-T02-IM6	Pancreatic neuroendocrine tumor	Metastasis	BRCA2	p.A2864T	FALSE	13	32945195	G	A	Missense_Mutation	0,056968464	983	FALSE	0.33	Subclonal
P-0013749-T02-IM6	Pancreatic neuroendocrine tumor	Metastasis	NKX2-1	p.A310V	FALSE	14	36986760	G	A	Missense_Mutation	0,06741573	356	FALSE	0.29	Subclonal
P-0013749-T02-IM6	Pancreatic neuroendocrine tumor	Metastasis	NTRK3	p.P792S	FALSE	15	88420312	G	A	Missense_Mutation	0,05588585	841	FALSE	0.35	Subclonal
P-0013749-T02-IM6	Pancreatic neuroendocrine tumor	Metastasis	PALB2	p.G232D	FALSE	16	23647172	C	T	Missense_Mutation	0,051100629	1272	FALSE	0.28	Subclonal
P-0013749-T02-IM6	Pancreatic neuroendocrine tumor	Metastasis	MAPK3	p.G232S	FALSE	16	30129072	C	T	Missense_Mutation	0,054030115	1129	FALSE	0.35	Subclonal
P-0013749-T02-IM6	Pancreatic neuroendocrine tumor	Metastasis	PLCG2	p.S55N	FALSE	16	81819758	G	A	Missense_Mutation	0,054621849	714	FALSE	0.28	Subclonal

Table S2

Sample ID	Cancer subtype	Sample type	Symbol	Aminoacid change	Hotspot	Chromosome	Position	Reference	Alternate	Mutation type	Tumor MAF	Tumor DP	LOH	Cancer cell fraction	Clonal status
P-0013749-T02-IM6	Pancreatic neuroendocrine tumor	Metastasis	FANCA	p.G1388D	FALSE	16	89805545	C	T	Missense_Mutation	0,053404539	749	FALSE	0.26	Subclonal
P-0013749-T02-IM6	Pancreatic neuroendocrine tumor	Metastasis	NCOR1	p.V2119I	FALSE	17	15960865	C	T	Missense_Mutation	0,054507338	477	FALSE	0.33	Subclonal
P-0013749-T02-IM6	Pancreatic neuroendocrine tumor	Metastasis	NCOR1	p.D100N	FALSE	17	16075254	C	T	Missense_Mutation	0,050605061	909	FALSE	0.29	Subclonal
P-0013749-T02-IM6	Pancreatic neuroendocrine tumor	Metastasis	NF1	p.N2659I	FALSE	17	29685503	A	T	Missense_Mutation	0,149466192	562	FALSE	0.79	Subclonal
P-0013749-T02-IM6	Pancreatic neuroendocrine tumor	Metastasis	STAT5A	p.P27L	FALSE	17	40441509	C	T	Missense_Mutation	0,053724928	1396	FALSE	0.28	Subclonal
P-0013749-T02-IM6	Pancreatic neuroendocrine tumor	Metastasis	PPM1D	p.M521I	FALSE	17	58740658	G	A	Missense_Mutation	0,054195804	1144	FALSE	0.35	Subclonal
P-0013749-T02-IM6	Pancreatic neuroendocrine tumor	Metastasis	H3F3B	p.A22T	FALSE	17	73775192	C	T	Missense_Mutation	0,057833859	951	FALSE	0.28	Subclonal
P-0013749-T02-IM6	Pancreatic neuroendocrine tumor	Metastasis	RPTOR	p.S1167N	FALSE	17	78933900	G	A	Missense_Mutation	0,053639847	1044	FALSE	0.26	Subclonal
P-0013749-T02-IM6	Pancreatic neuroendocrine tumor	Metastasis	YES1	p.G294E	FALSE	18	743097	C	T	Missense_Mutation	0,054587689	861	FALSE	0.33	Subclonal
P-0013749-T02-IM6	Pancreatic neuroendocrine tumor	Metastasis	PIK3C3	p.A80T	FALSE	18	39537704	G	A	Missense_Mutation	0,051529791	621	FALSE	0.29	Subclonal
P-0013749-T02-IM6	Pancreatic neuroendocrine tumor	Metastasis	DOT1L	p.A1345T	FALSE	19	2226553	G	A	Missense_Mutation	0,057619409	1319	FALSE	0.35	Subclonal
P-0013749-T02-IM6	Pancreatic neuroendocrine tumor	Metastasis	DOT1L	p.G1490S	FALSE	19	2226988	G	A	Missense_Mutation	0,060663507	1055	FALSE	0.28	Subclonal
P-0013749-T02-IM6	Pancreatic neuroendocrine tumor	Metastasis	DNAJB1	p.R30L	FALSE	19	14629073	C	A	Missense_Mutation	0,054755043	694	FALSE	0.35	Subclonal
P-0013749-T02-IM6	Pancreatic neuroendocrine tumor	Metastasis	NOTCH3	p.T2282I	FALSE	19	15271594	G	A	Missense_Mutation	0,050970874	1648	FALSE	0.28	Subclonal
P-0013749-T02-IM6	Pancreatic neuroendocrine tumor	Metastasis	NOTCH3	p.G413S	FALSE	19	15299941	C	T	Missense_Mutation	0,061293031	1191	FALSE	0.26	Subclonal
P-0013749-T02-IM6	Pancreatic neuroendocrine tumor	Metastasis	NOTCH3	p.P197S	FALSE	19	15302861	G	A	Missense_Mutation	0,050624133	1442	FALSE	0.33	Subclonal
P-0013749-T02-IM6	Pancreatic neuroendocrine tumor	Metastasis	MLL4	p.G1817D	FALSE	19	36222821	G	A	Missense_Mutation	0,057719054	1438	FALSE	0.29	Subclonal
P-0013749-T02-IM6	Pancreatic neuroendocrine tumor	Metastasis	ALK	p.G1236D	FALSE	2	29436886	C	T	Missense_Mutation	0,055982437	911	FALSE	0.35	Subclonal
P-0013749-T02-IM6	Pancreatic neuroendocrine tumor	Metastasis	ALK	p.W554*	FALSE	2	29519909	C	T	Nonsense_Mutation	0,064120055	733	FALSE	0.28	Subclonal
P-0013749-T02-IM6	Pancreatic neuroendocrine tumor	Metastasis	ERBB4	p.D1257N	FALSE	2	212248498	C	T	Missense_Mutation	0,054242003	719	FALSE	0.35	Subclonal
P-0013749-T02-IM6	Pancreatic neuroendocrine tumor	Metastasis	ERBB4	p.D1179N	FALSE	2	212248732	C	T	Missense_Mutation	0,055674518	467	FALSE	0.28	Subclonal
P-0013749-T02-IM6	Pancreatic neuroendocrine tumor	Metastasis	IRS1	p.R353K	FALSE	2	227662397	C	T	Missense_Mutation	0,075887393	817	FALSE	0.26	Subclonal
P-0013749-T02-IM6	Pancreatic neuroendocrine tumor	Metastasis	IRS1	p.G234E	FALSE	2	227662754	C	T	Missense_Mutation	0,058718861	1124	FALSE	0.33	Subclonal
P-0013749-T02-IM6	Pancreatic neuroendocrine tumor	Metastasis	PTPRT	p.S1458N	FALSE	20	40709529	C	T	Missense_Mutation	0,057630736	937	FALSE	0.29	Subclonal
P-0013749-T02-IM6	Pancreatic neuroendocrine tumor	Metastasis	PTPRT	p.G905S	FALSE	20	40790018	C	T	Missense_Mutation	0,055350554	813	FALSE	0.35	Subclonal
P-0013749-T02-IM6	Pancreatic neuroendocrine tumor	Metastasis	RTEL1	p.X132_splice	FALSE	20	62293297	G	A	Splice_Site	0,057692308	936	FALSE	0.28	Subclonal
P-0013749-T02-IM6	Pancreatic neuroendocrine tumor	Metastasis	RTEL1	p.G431S	FALSE	20	62316903	G	A	Missense_Mutation	0,050813008	984	FALSE	0.35	Subclonal
P-0013749-T02-IM6	Pancreatic neuroendocrine tumor	Metastasis	RTEL1	p.P911L	FALSE	20	62324165	C	T	Missense_Mutation	0,060381356	944	FALSE	0.28	Subclonal
P-0013749-T02-IM6	Pancreatic neuroendocrine tumor	Metastasis	CHEK2	p.S35F	FALSE	22	29130606	G	A	Missense_Mutation	0,054545455	770	FALSE	0.26	Subclonal
P-0013749-T02-IM6	Pancreatic neuroendocrine tumor	Metastasis	EP300	p.X1342_splice	FALSE	22	41564604	G	A	Splice_Site	0,059912854	918	FALSE	0.33	Subclonal
P-0013749-T02-IM6	Pancreatic neuroendocrine tumor	Metastasis	MLH1	p.Q427*	FALSE	3	37067368	C	T	Nonsense_Mutation	0,128767123	730	FALSE	0.79	Subclonal
P-0013749-T02-IM6	Pancreatic neuroendocrine tumor	Metastasis	SETD2	p.A1591V	FALSE	3	47147554	G	A	Missense_Mutation	0,055555556	846	FALSE	0.35	Subclonal
P-0013749-T02-IM6	Pancreatic neuroendocrine tumor	Metastasis	SETD2	p.S1276N	FALSE	3	47162299	C	T	Missense_Mutation	0,054673721	567	FALSE	0.28	Subclonal
P-0013749-T02-IM6	Pancreatic neuroendocrine tumor	Metastasis	MST1R	p.G1155D	FALSE	3	49928902	C	T	Missense_Mutation	0,056505576	1345	FALSE	0.35	Subclonal
P-0013749-T02-IM6	Pancreatic neuroendocrine tumor	Metastasis	MST1R	p.A891T	FALSE	3	49933519	C	T	Missense_Mutation	0,053888131	1466	FALSE	0.28	Subclonal
P-0013749-T02-IM6	Pancreatic neuroendocrine tumor	Metastasis	PBRM1	p.A482T	FALSE	3	52661386	C	T	Missense_Mutation	0,051896208	501	FALSE	0.26	Subclonal
P-0013749-T02-IM6	Pancreatic neuroendocrine tumor	Metastasis	GATA2	p.P41S	FALSE	3	128205754	G	A	Missense_Mutation	0,052863436	1135	FALSE	0.33	Subclonal
P-0013749-T02-IM6	Pancreatic neuroendocrine tumor	Metastasis	ATR	p.L107F	FALSE	3	142281925	G	A	Missense_Mutation	0,050235479	637	FALSE	0.29	Subclonal
P-0013749-T02-IM6	Pancreatic neuroendocrine tumor	Metastasis	FGFR3	p.S400N	FALSE	4	1806180	G	A	Missense_Mutation	0,05	1400	FALSE	0.35	Subclonal
P-0013749-T02-IM6	Pancreatic neuroendocrine tumor	Metastasis	WHSC1	p.S30N	FALSE	4	1902470	G	A	Missense_Mutation	0,050895382	1061	FALSE	0.28	Subclonal
P-0013749-T02-IM6	Pancreatic neuroendocrine tumor	Metastasis	FAT1	p.P4216S	FALSE	4	187518048	G	A	Missense_Mutation	0,051369863	876	FALSE	0.35	Subclonal
P-0013749-T02-IM6	Pancreatic neuroendocrine tumor	Metastasis	TERT	p.A750V	FALSE	5	1278793	G	A	Missense_Mutation	0,052410901	954	FALSE	0.28	Subclonal
P-0013749-T02-IM6	Pancreatic neuroendocrine tumor	Metastasis	TERT	p.T328I	FALSE	5	1294018	G	A	Missense_Mutation	0,050522648	1722	FALSE	0.26	Subclonal
P-0013749-T02-IM6	Pancreatic neuroendocrine tumor	Metastasis	RASA1	p.D469N	FALSE	5	86658440	G	A	Missense_Mutation	0,087954111	523	FALSE	0.33	Subclonal
P-0013749-T02-IM6	Pancreatic neuroendocrine tumor	Metastasis	APC	p.E892K	FALSE	5	112173965	G	A	Missense_Mutation	0,051748252	715	FALSE	0.29	Subclonal
P-0013749-T02-IM6	Pancreatic neuroendocrine tumor	Metastasis	NSD1	p.A1843T	FALSE	5	176700690	G	A	Missense_Mutation	0,050429185	932	FALSE	0.35	Subclonal
P-0013749-T02-IM6	Pancreatic neuroendocrine tumor	Metastasis	MDC1	p.V940M	FALSE	6	30675538	C	T	Missense_Mutation	0,073975045	1122	FALSE	0.35	Subclonal
P-0013749-T02-IM6	Pancreatic neuroendocrine tumor	Metastasis	STK19	p.W285*	FALSE	6	31948279	G	A	Nonsense_Mutation	0,053037608	1037	FALSE	0.28	Subclonal
P-0013749-T02-IM6	Pancreatic neuroendocrine tumor	Metastasis	TAP2	p.P186L	FALSE	6	32805365	G	A	Missense_Mutation	0,050373134	1072	FALSE	0.35	Subclonal
P-0013749-T02-IM6	Pancreatic neuroendocrine tumor	Metastasis	DAXX	p.A487T	FALSE	6	33287794	C	T	Missense_Mutation	0,051020408	1274	FALSE	0.28	Subclonal
P-0013749-T02-IM6	Pancreatic neuroendocrine tumor	Metastasis	ARID1B	p.P176L	FALSE	6	157099590	C	T	Missense_Mutation	0,098360656	305	FALSE	0.56	Subclonal
P-0013749-T02-IM6	Pancreatic neuroendocrine tumor	Metastasis	PMS2	p.S8N	FALSE	7	6048628	C	T	Missense_Mutation	0,053772767	1153	FALSE	0.33	Subclonal
P-0013749-T02-IM6	Pancreatic neuroendocrine tumor	Metastasis	MET	p.R304K	FALSE	7	116340049	G	A	Missense_Mutation	0,050108932	459	FALSE	0.29	Subclonal
P-0013749-T02-IM6	Pancreatic neuroendocrine tumor	Metastasis	MET	p.V1155M	FALSE	7	116418952	G	A	Missense_Mutation	0,061538462	650	FALSE	0.35	Subclonal
P-0013749-T02-IM6	Pancreatic neuroendocrine tumor	Metastasis	BRAF	p.G596D	TRUE	7	140453148	C	T	Missense_Mutation	0,026058632	921	FALSE	0.28	Subclonal
P-0013749-T02-IM6	Pancreatic neuroendocrine tumor	Metastasis	BRAF	p.E451K	FALSE	7	140481457	C	T	Missense_Mutation	0,058311575	1149	FALSE	0.35	Subclonal
P-0013749-T02-IM6	Pancreatic neuroendocrine tumor	Metastasis	MLL3	p.M4124I	FALSE	7	151849944	C	T	Missense_Mutation	0,05	800	FALSE	0.28	Subclonal
P-0013749-T02-IM6	Pancreatic neuroendocrine tumor	Metastasis	SOX17	p.H368Y	FALSE	8	55372412	C	T	Missense_Mutation	0,054200542	1476	FALSE	0.26	Subclonal
P-0013749-T02-IM6	Pancreatic neuroendocrine tumor	Metastasis	LYN	p.W240*	FALSE	8	56866473	G	A	Nonsense_Mutation	0,055408971	1137	FALSE	0.33	Subclonal
P-0013749-T02-IM6	Pancreatic neuroendocrine tumor	Metastasis	JAK2	p.X1060_splice	FALSE	9	5126332	G	A	Splice_Site	0,063694268	471	FALSE	0.29	Subclonal
P-0013749-T02-IM6	Pancreatic neuroendocrine tumor	Metastasis	PTPRD	p.P1129S	FALSE	9	8484147	G	A	Missense_Mutation	0,056192661	872	FALSE	0.35	Subclonal
P-0013749-T02-IM6	Pancreatic neuroendocrine tumor	Metastasis	PAX5	p.G378E	FALSE	9	36840600	C	T	Missense_Mutation	0,058823529	1292	FALSE	0.28	Subclonal
P-0013749-T02-IM6	Pancreatic neuroendocrine tumor	Metastasis	SYK	p.E144K	FALSE	9	93607728	G	A	Missense_Mutation	0,051851852	540	FALSE	0.35	Subclonal
P-0013749-T02-IM6	Pancreatic neuroendocrine tumor	Metastasis	SYK	p.H465Y	FALSE	9	93641047	C	T	Missense_Mutation	0,063333333	600	FALSE	0.28	Subclonal
P-0013749-T02-IM6	Pancreatic neuroendocrine tumor	Metastasis	PTCH1	p.G1396E	FALSE	9	98209351	C	T	Missense_Mutation	0,052508751	857	FALSE	0.26	Subclonal
P-0013749-T02-IM6	Pancreatic neuroendocrine tumor	Metastasis	ABL1	p.P978S	FALSE	9	133760609	C	T	Missense_Mutation	0,051108374	1624	FALSE	0.33	Subclonal
P-0013749-T02-IM6	Pancreatic neuroendocrine tumor	Metastasis	NOTCH1	p.P1236L	FALSE	9	139401362	G	A	Missense_Mutation	0,060016221	1233	FALSE	0.29	Subclonal
P-0013749-T02-IM6	Pancreatic neuroendocrine tumor	Metastasis	MED12	p.A767T	FALSE	X	70345273	G	A	Missense_Mutation	0,052795031	1288	FALSE	0.35	Subclonal
P-0014036-T01-IM5	Pancreatic neuroendocrine tumor	Metastasis	KRAS	p.L19F	TRUE	12	25398262	C	G	Missense_Mutation	0,359560842	1093	FALSE	0.98	Clonal
P-0014036-T01-IM5	Pancreatic neuroendocrine tumor	Metastasis	ATRX	p.S1326*	FALSE	X	76919014	G	C	Nonsense_Mutation	0,630065359	765	TRUE	1	Clonal
P-0014036-T01-IM5	Pancreatic neuroendocrine tumor	Metastasis	MEN1	p.R92Qfs*25	FALSE	11	64577305	AGC	GCTT	Frame_Shift_Ins	0,634517766	788	TRUE	1	Clonal
P-0015723-T01-IM6	Pancreatic neuroendocrine tumor	Metastasis	TSC2	p.R1639H	FALSE	16	2136799	G	A	Missense_Mutation	0,528055342	1301	TRUE	1	Clonal
P-0015723-T01-IM6	Pancreatic neuroendocrine tumor	Metastasis	TP53	p.P190S	TRUE	17	7578281	G	A	Missense_Mutation	0,465277778	864	TRUE	1	Clonal
P-0015723-T01-IM6	Pancreatic neuroendocrine tumor	Metastasis	CUL3	p.R756Q	FALSE	2	225339002	C	T	Missense_Mutation	0,050209205	717	FALSE	0.12	Subclonal
P-0015723-T01-IM6	Pancreatic neuroendocrine tumor	Metastasis	ARID2	p.V1486A	FALSE	12	46246363	T	C	Missense_Mutation	0,33877551	735	FALSE	1	Clonal
P-0015723-T01-IM6	Pancreatic neuroendocrine tumor	Metastasis	ERBB3	p.G508R	FALSE	12	56487589	G	A	Missense_Mutation	0,316883117	1155	FALSE	1	Clonal
P-0015723-T01-IM6	Pancreatic neuroendocrine tumor	Metastasis	TERT	p.R865C	FALSE	5	1266640	G	A	Missense_Mutation	0,325852783	1114	FALSE	1	Clonal
P-0015723-T01-IM6	Pancreatic neuroendocrine tumor	Metastasis	PTP4A1	p.L117V	FALSE	6	64289181	C	G	Missense_Mutation	0,640955005	1089	FALSE	1	Clonal
P-0015723-T01-IM6	Pancreatic neuroendocrine tumor	Metastasis	PRDM1	p.E252Q	FALSE	6	106552789	G	C	Missense_Mutation	0,415026834	1118	FALSE	1	Clonal
P-0015723-T01-IM6	Pancreatic neuroendocrine tumor	Metastasis	ATRX	p.R2153L	FALSE	X	76814186	C	A	Missense_Mutation	0,461832061	524	TRUE	1	Clonal
P-0017348-T01-IM6	Pancreatic neuroendocrine tumor	Metastasis	MEN1	p.W441L	FALSE	11	64572549	C	A	Missense_Mutation	0,58526603	733	TRUE	1	Clonal

Table S2

Sample ID	Cancer subtype	Sample type	Symbol	Aminoacid change	Hotspot	Chromosome	Position	Reference	Alternate	Mutation type	Tumor MAF	Tumor DP	LOH	Cancer cell fraction	Clonal status
P-0017348-T01-IM6	Pancreatic neuroendocrine tumor	Metastasis	TSC2	p.X880_splice	FALSE	16	2125942	GGCCTTGCGGCAGAAAGCCCCGGCAGCCTTTGTCCCCAAGGCCTGAGCGC CTCGGTTTTTTGCACTTCATGCCCTGGGGATGTTTCCCTGCTGCCAGGATGG AGTGCCAGCCCCCTTCTCATCTCAGGTTTAATCAGTACATCGTGTGTCTGGC CCATCACGTCATAGCCATGTGGTTCATCAGGTGCCGCCTGCCCTTCCGGAA GGATTTTGTCCCTTTCATCACTAAGGTGGGCTCAGGGCCGGTGAAGGCTGT GTCTCTCGGTAGGCCAGGGCTTGCTTTGCCCTTGGCTGTCCATGGTCGGGC AGAGTGACAGGCAGGTGGAGGGCAGTGGGAGGGTGTTTGGGGCTGTGCCT GTGGCGCTGGGGGCTCTGCTGGGCAGCCTGCAGGGCTTTGATGCGCGGCA GGCATTGAGGGGTGGGAGCTGGGTGCCGCCGCCTTGCCCCTAGCCTGCAG CTTGTCCCTGGCCAGGGGGCACCCGGCAGGCCTGGTGAGGGCCTCCAGCC CCCATTGCCACCCCTCACTGTCTG	-	Splice_Site	0,677057357	802	TRUE	1	Clonal
P-0017348-T01-IM6	Pancreatic neuroendocrine tumor	Metastasis	ATRX	p.K1936Rfs*19	FALSE	X	76855029	T	-	Frame_Shift_Del	0,354700855	468	TRUE	1	Clonal
P-0017852-T01-IM6	Pancreatic neuroendocrine tumor	Metastasis	ATRX	p.V1625Sfs*3	FALSE	X	76889133	CAAAC	-	Frame_Shift_Del	0,247557003	307	FALSE	1	Clonal
P-0026064-T01-IM6	Pancreatic neuroendocrine tumor	Metastasis	TERT	p.A288V	FALSE	5	1294138	G	A	Missense_Mutation	0,436775262	1811	FALSE	1	Clonal
P-0026064-T01-IM6	Pancreatic neuroendocrine tumor	Metastasis	MEN1	p.X223_splice	FALSE	11	64575362	C	A	Splice_Site	0,797658863	598	TRUE	1	Clonal
P-0026064-T01-IM6	Pancreatic neuroendocrine tumor	Metastasis	ATM	p.Y380C	FALSE	11	108119733	A	G	Missense_Mutation	0,113157895	380	TRUE	0.32	Subclonal
P-0026064-T01-IM6	Pancreatic neuroendocrine tumor	Metastasis	GLI1	p.R418W	FALSE	12	57861951	C	T	Missense_Mutation	0,413957935	1046	FALSE	1	Clonal
P-0026064-T01-IM6	Pancreatic neuroendocrine tumor	Metastasis	ERCC5	p.I634T	FALSE	13	103515400	T	C	Missense_Mutation	0,051006711	745	FALSE	0.24	Subclonal
P-0026064-T01-IM6	Pancreatic neuroendocrine tumor	Metastasis	EP300	p.R1732C	FALSE	22	41572909	C	T	Missense_Mutation	0,775596073	713	FALSE	1	Clonal
P-0026064-T01-IM6	Pancreatic neuroendocrine tumor	Metastasis	E2F3	p.T342A	FALSE	6	20488368	A	G	Missense_Mutation	0,063380282	710	FALSE	0.18	Subclonal
P-0031836-T01-IM6	Pancreatic neuroendocrine tumor	Metastasis	RBM10	p.G342D	FALSE	X	47040624	G	A	Missense_Mutation	0,140322581	620	FALSE	0.35	Subclonal
P-0031836-T01-IM6	Pancreatic neuroendocrine tumor	Metastasis	MEN1	p.S114Gfs*67	FALSE	11	64577232	AGCTCACGGCT	-	Frame_Shift_Del	0,689393939	528	TRUE	1	Clonal
P-0031836-T01-IM6	Pancreatic neuroendocrine tumor	Metastasis	SOX9	p.D482N	FALSE	17	70120442	G	A	Missense_Mutation	0,081444759	1412	FALSE	0.2	Subclonal
P-0031836-T01-IM6	Pancreatic neuroendocrine tumor	Metastasis	ATRX	p.E2264A	FALSE	X	76778788	T	G	Missense_Mutation	0,418803419	702	FALSE	1	Clonal
P-0032629-T01-IM6	Pancreatic neuroendocrine tumor	Metastasis	MEN1	p.P12_R29dup	FALSE	11	64577494	-	TCGGCCCAGCTCGGCAGC AAACAGGCGCACCACGTCG TCGATGGAGCGCAGCGG	In_Frame_Ins	0,061363636	440	FALSE	0.37	Subclonal
P-0032629-T01-IM6	Pancreatic neuroendocrine tumor	Metastasis	ATRX	p.S978_E979delins*	FALSE	X	76937813	CAG	-	Nonsense_Mutation	0,256756757	222	FALSE	1	Clonal
P-0035025-T02-IM6	Pancreatic neuroendocrine tumor	Metastasis	RB1	p.R661W	FALSE	13	49033844	C	T	Missense_Mutation	0,815856777	391	TRUE	1	Clonal
P-0035025-T02-IM6	Pancreatic neuroendocrine tumor	Metastasis	RPS6KB2	p.R329W	FALSE	11	67201684	C	T	Missense_Mutation	0,823104693	554	FALSE	1	Clonal
P-0035025-T02-IM6	Pancreatic neuroendocrine tumor	Metastasis	RBM10	p.R648*	FALSE	X	47044850	C	T	Nonsense_Mutation	0,378600823	729	FALSE	0.95	Clonal
P-0035025-T02-IM6	Pancreatic neuroendocrine tumor	Metastasis	INPPL1	p.N441Kfs*25	FALSE	11	71942052	-	A	Frame_Shift_Ins	0,76993865	652	FALSE	1	Clonal
P-0035025-T02-IM6	Pancreatic neuroendocrine tumor	Metastasis	CSDE1	p.T368A	FALSE	1	115275311	T	C	Missense_Mutation	0,79491256	629	FALSE	1	Clonal
P-0035025-T02-IM6	Pancreatic neuroendocrine tumor	Metastasis	MEN1	p.A5Gfs*111	FALSE	11	64577567	GG	-	Frame_Shift_Del	0,807162534	363	TRUE	1	Clonal
P-0035025-T02-IM6	Pancreatic neuroendocrine tumor	Metastasis	ZFHX3	p.V418Afs*39	FALSE	16	72992792	A	-	Frame_Shift_Del	0,783542039	559	FALSE	1	Clonal
P-0035025-T02-IM6	Pancreatic neuroendocrine tumor	Metastasis	ATRX	p.D1383Ffs*106	FALSE	X	76912114	AATC	-	Frame_Shift_Del	0,8	315	TRUE	1	Clonal
P-0035025-T02-IM6	Pancreatic neuroendocrine tumor	Metastasis	NOTCH3	p.G2059R	FALSE	19	15272264	C	T	Missense_Mutation	0,368631369	1001	FALSE	1	Clonal
P-0035025-T02-IM6	Pancreatic neuroendocrine tumor	Metastasis	MDC1	p.V1078I	FALSE	6	30673728	C	T	Missense_Mutation	0,121259843	635	FALSE	0.32	Subclonal
P-0035025-T02-IM6	Pancreatic neuroendocrine tumor	Metastasis	PARK2	p.Q317K	FALSE	6	161970020	G	T	Missense_Mutation	0,300324675	616	FALSE	0.8	Subclonal
P-0040534-T01-IM6	Pancreatic neuroendocrine tumor	Metastasis	KEAP1	p.D422N	FALSE	19	10602314	C	T	Missense_Mutation	0,052332913	1586	FALSE	0.17	Subclonal
P-0040534-T01-IM6	Pancreatic neuroendocrine tumor	Metastasis	MEN1	p.W441*	FALSE	11	64572549	C	T	Nonsense_Mutation	0,485743381	982	TRUE	1	Clonal
P-0040534-T01-IM6	Pancreatic neuroendocrine tumor	Metastasis	ATRX	p.P1711L	FALSE	X	76888697	G	A	Missense_Mutation	0,654867257	339	TRUE	1	Clonal
P-0040973-T01-IM6	Pancreatic neuroendocrine tumor	Metastasis	PREX2	p.E550Q	FALSE	8	68992683	G	C	Missense_Mutation	0,417763158	304	FALSE	1	Clonal
P-0040973-T01-IM6	Pancreatic neuroendocrine tumor	Metastasis	MEN1	p.L41Rfs*71	FALSE	11	64577439	AGAAAATGCTCCACGAAGCCCA	-	Frame_Shift_Del	0,634328358	536	TRUE	0.97	Clonal
P-0040973-T01-IM6	Pancreatic neuroendocrine tumor	Metastasis	PIK3CG	p.L1018V	FALSE	7	106545575	C	G	Missense_Mutation	0,090909091	396	FALSE	0.23	Subclonal
P-0040973-T01-IM6	Pancreatic neuroendocrine tumor	Metastasis	ATRX	p.E447A	FALSE	X	76939408	T	G	Missense_Mutation	0,147058824	68	FALSE	0.43	Subclonal
P-0000879-T03-IM5	Pancreatic neuroendocrine tumor	Metastasis	BRAF	p.V600E	TRUE	7	140453136	A	T	Missense_Mutation	0,418478261	736	FALSE	1	Clonal
P-0000879-T03-IM5	Pancreatic neuroendocrine tumor	Metastasis	NRAS	p.Q61R	TRUE	1	115256529	T	C	Missense_Mutation	0,371541502	506	FALSE	0.96	Clonal
P-0000879-T03-IM5	Pancreatic neuroendocrine tumor	Metastasis	PIK3CG	p.V1014A	FALSE	7	106545564	T	C	Missense_Mutation	0,187853107	708	FALSE	0.86	Clonal
P-0000879-T03-IM5	Pancreatic neuroendocrine tumor	Metastasis	YAP1	p.I167T	FALSE	11	101985053	T	C	Missense_Mutation	0,270992366	786	FALSE	0.97	Clonal
P-0000879-T03-IM5	Pancreatic neuroendocrine tumor	Metastasis	SH2B3	p.L429P	FALSE	12	111885509	T	C	Missense_Mutation	0,130870712	1895	FALSE	0.47	Subclonal
P-0000879-T03-IM5	Pancreatic neuroendocrine tumor	Metastasis	BRIP1	p.S988Y	FALSE	17	59761444	G	T	Missense_Mutation	0,235	600	FALSE	0.84	Clonal
P-0001526-T02-IM5	Pancreatic neuroendocrine tumor	Metastasis	MEN1	p.X267_splice	FALSE	11	64574693	T	C	Splice_Site	0,545064378	466	TRUE	1	Clonal
P-0001526-T02-IM5	Pancreatic neuroendocrine tumor	Metastasis	EPHB1	p.Y308C	FALSE	3	134825407	A	G	Missense_Mutation	0,150763359	524	FALSE	0.3	Subclonal
P-0001526-T02-IM5	Pancreatic neuroendocrine tumor	Metastasis	DAXX	p.E410*	FALSE	6	33288180	C	A	Nonsense_Mutation	0,482758621	261	TRUE	0.97	Clonal
P-0001526-T02-IM5	Pancreatic neuroendocrine tumor	Metastasis	ATM	p.N1719D	FALSE	11	108170590	A	G	Missense_Mutation	0,183962264	212	FALSE	0.37	Subclonal
P-0001526-T02-IM5	Pancreatic neuroendocrine tumor	Metastasis	BRCA2	p.S1968P	FALSE	13	32914394	T	C	Missense_Mutation	0,121281465	437	FALSE	0.36	Subclonal
P-0001943-T02-IM5	Pancreatic neuroendocrine tumor	Metastasis	ARID1A	p.X1180_splice	FALSE	1	27099124	G	T	Splice_Site	0,109433962	265	FALSE	0.43	Subclonal
P-0001943-T02-IM5	Pancreatic neuroendocrine tumor	Metastasis	SETD2	p.E2034*	FALSE	3	47108569	C	A	Nonsense_Mutation	0,16254417	283	FALSE	0.63	Subclonal
P-0001943-T02-IM5	Pancreatic neuroendocrine tumor	Metastasis	FAT1	p.V1653G	FALSE	4	187542782	A	C	Missense_Mutation	0,158490566	265	FALSE	0.62	Subclonal
P-0001993-T01-IM3	Pancreatic neuroendocrine tumor	Metastasis	MEN1	p.Q64*	FALSE	11	64577392	G	A	Nonsense_Mutation	0,455205811	413	TRUE	0.73	Subclonal
P-0001993-T01-IM3	Pancreatic neuroendocrine tumor	Metastasis	TSC2	p.X161_splice	FALSE	16	2105402	G	A	Splice_Site	0,260869565	253	FALSE	0.68	Subclonal
P-0001993-T01-IM3	Pancreatic neuroendocrine tumor	Metastasis	DAXX	p.L191Wfs*38	FALSE	6	33288980	A	-	Frame_Shift_Del	0,525114155	438	TRUE	1	Clonal
P-0002103-T01-IM3	Pancreatic neuroendocrine tumor	Metastasis	INPP4B	p.L423R	FALSE	4	143094876	A	C	Missense_Mutation	0,084444444	1575	FALSE	0.73	Subclonal
P-0002103-T01-IM3	Pancreatic neuroendocrine tumor	Metastasis	NOTCH4	p.G1070C	FALSE	6	32171570	C	A	Missense_Mutation	0,111595466	1147	FALSE	0.96	Clonal
P-0002103-T01-IM3	Pancreatic neuroendocrine tumor	Metastasis	TSC1	p.K629*	FALSE	9	135781080	T	A	Nonsense_Mutation	0,118953212	1261	FALSE	1	Clonal
P-0002202-T01-IM3	Pancreatic neuroendocrine tumor	Metastasis	SMAD4	p.D537V	FALSE	18	48604788	A	T	Missense_Mutation	0,830892144	751	TRUE	0.98	Clonal
P-0002202-T01-IM3	Pancreatic neuroendocrine tumor	Metastasis	DAXX	p.L532*	FALSE	6	33287502	A	T	Nonsense_Mutation	0,838797814	732	TRUE	0.99	Clonal
P-0002202-T01-IM3	Pancreatic neuroendocrine tumor	Metastasis	MEN1	p.A169V	FALSE	11	64575526	G	A	Missense_Mutation	0,831967213	732	TRUE	0.98	Clonal
P-0002253-T02-IM5	Pancreatic neuroendocrine tumor	Metastasis	SETD2	p.R1424Efs*8	FALSE	3	47161856	T	-	Frame_Shift_Del	0,1328125	768	FALSE	0.38	Subclonal
P-0002253-T02-IM5	Pancreatic neuroendocrine tumor	Metastasis	DAXX	p.A103Sfs*40	FALSE	6	33289242	CCGC	-	Frame_Shift_Del	0,332467532	385	FALSE	0.95	Clonal
P-0002253-T02-IM5	Pancreatic neuroendocrine tumor	Metastasis	MEN1	p.N194Mfs*35	FALSE	11	64575456	C	-	Frame_Shift_Del	0,32123412	551	FALSE	0.92	Clonal
P-0002253-T02-IM5	Pancreatic neuroendocrine tumor	Metastasis	RHEB	p.Y35S	FALSE	7	151188049	T	G	Missense_Mutation	0,138263666	933	FALSE	0.39	Subclonal
P-0004307-T02-IM6	Pancreatic neuroendocrine tumor	Metastasis	DAXX	p.Q193*	FALSE	6	33288975	G	A	Nonsense_Mutation	0,734741784	426	TRUE	1	Clonal
P-0004307-T02-IM6	Pancreatic neuroendocrine tumor	Metastasis	MEN1	p.X263_splice	FALSE	11	64575021	CACCTGCTGCAGCTG	-	Splice_Site	0,752	375	TRUE	1	Clonal
P-0004364-T01-IM5	Pancreatic neuroendocrine tumor	Metastasis	BRAF	p.V600E	TRUE	7	140453136	A	T	Missense_Mutation	0,677816901	568	FALSE	1	Clonal
P-0004364-T01-IM5	Pancreatic neuroendocrine tumor	Metastasis	ARID1A	p.K1808*	FALSE	1	27105811	A	T	Nonsense_Mutation	0,596205962	369	TRUE	1	Clonal
P-0004364-T01-IM5	Pancreatic neuroendocrine tumor	Metastasis	NOTCH3	p.R1848H	FALSE	19	15276722	C	T	Missense_Mutation	0,284061697	778	FALSE	1	Clonal
P-0004595-T02-IM5	Pancreatic neuroendocrine tumor	Metastasis	TP53	p.R273C	TRUE	17	7577121	G	A	Missense_Mutation	0,445157526	1714	FALSE	1	Clonal
P-0004595-T02-IM5	Pancreatic neuroendocrine tumor	Metastasis	TSC2	p.X161_splice	FALSE	16	2105402	G	A	Splice_Site	0,690288714	762	TRUE	1	Clonal
P-0004595-T02-IM5	Pancreatic neuroendocrine tumor	Metastasis	CREBBP	p.P1488L	FALSE	16	3786748	G	A	Missense_Mutation	0,688785047	1070	TRUE	1	Clonal
P-0004595-T02-IM5	Pancreatic neuroendocrine tumor	Metastasis	BRAF	p.T310I	FALSE	7	140500213	G	A	Missense_Mutation	0,728689276	1091	TRUE	1	Clonal

Table S2

Sample ID	Cancer subtype	Sample type	Symbol	Aminoacid change	Hotspot	Chromosome	Position	Reference	Alternate	Mutation type	Tumor MAF	Tumor DP	LOH	Cancer cell fraction	Clonal status
P-0004595-T02-IM5	Pancreatic neuroendocrine tumor	Metastasis	MEN1	p.W346Gfs*27	FALSE	11	64573732	A	-	Frame_Shift_Del	0,68115942	690	TRUE	1	Clonal
P-0004595-T02-IM5	Pancreatic neuroendocrine tumor	Metastasis	LATS2	p.A428T	FALSE	13	21562637	C	T	Missense_Mutation	0,459051724	464	FALSE	1	Clonal
P-0004595-T02-IM5	Pancreatic neuroendocrine tumor	Metastasis	RB1	p.V493I	FALSE	13	48954356	G	A	Missense_Mutation	0,338907469	897	FALSE	0.85	Clonal
P-0004595-T02-IM5	Pancreatic neuroendocrine tumor	Metastasis	DIS3	p.X931_splice	FALSE	13	73334666	C	T	Splice_Site	0,371478873	1136	FALSE	0.94	Clonal
P-0004595-T02-IM5	Pancreatic neuroendocrine tumor	Metastasis	BRCA1	p.S288N	FALSE	17	41246685	C	T	Missense_Mutation	0,3977433	1418	FALSE	1	Clonal
P-0004595-T02-IM5	Pancreatic neuroendocrine tumor	Metastasis	AKT2	p.G16D	FALSE	19	40762961	C	T	Missense_Mutation	0,398779247	983	FALSE	1	Clonal
P-0004595-T02-IM5	Pancreatic neuroendocrine tumor	Metastasis	ASXL2	p.D609N	FALSE	2	25972600	C	T	Missense_Mutation	0,707637232	838	FALSE	1	Clonal
P-0004595-T02-IM5	Pancreatic neuroendocrine tumor	Metastasis	MSH2	p.Q662*	FALSE	2	47702388	C	T	Nonsense_Mutation	0,643527205	533	FALSE	0.98	Clonal
P-0004595-T02-IM5	Pancreatic neuroendocrine tumor	Metastasis	ASXL1	p.A158T	FALSE	20	31017141	G	A	Missense_Mutation	0,409052092	1171	FALSE	1	Clonal
P-0004595-T02-IM5	Pancreatic neuroendocrine tumor	Metastasis	SETD2	p.P195Sfs*38	FALSE	3	47165534	GGGCAGGTGGA	-	Frame_Shift_Del	0,685404339	1014	TRUE	1	Clonal
P-0004595-T02-IM5	Pancreatic neuroendocrine tumor	Metastasis	DAXX	p.S102L	FALSE	6	33289247	G	A	Missense_Mutation	0,692073171	656	TRUE	1	Clonal
P-0004595-T02-IM5	Pancreatic neuroendocrine tumor	Metastasis	ARID1B	p.P438S	FALSE	6	157100375	C	T	Missense_Mutation	0,747774481	337	TRUE	1	Clonal
P-0004595-T02-IM5	Pancreatic neuroendocrine tumor	Metastasis	NOTCH1	p.G2348D	FALSE	9	139391148	C	T	Missense_Mutation	0,431203931	814	FALSE	1	Clonal
P-0004644-T01-IM5	Pancreatic neuroendocrine tumor	Metastasis	ATM	p.M2935I	FALSE	11	108225556	G	T	Missense_Mutation	0,22815534	824	FALSE	0.32	Subclonal
P-0004644-T01-IM5	Pancreatic neuroendocrine tumor	Metastasis	SETD2	p.L2328Rfs*36	FALSE	3	47088079	CTGGATATACTGAA	-	Frame_Shift_Del	0,256097561	492	TRUE	0.36	Subclonal
P-0005434-T01-IM5	Pancreatic neuroendocrine tumor	Metastasis	CTNNB1	p.S45F	TRUE	3	41266137	C	T	Missense_Mutation	0,35345667	1027	FALSE	1	Clonal
P-0005434-T01-IM5	Pancreatic neuroendocrine tumor	Metastasis	ARID2	p.Q507*	FALSE	12	46240659	C	T	Nonsense_Mutation	0,327731092	952	FALSE	0.96	Clonal
P-0005434-T01-IM5	Pancreatic neuroendocrine tumor	Metastasis	TP53	p.H179Y	TRUE	17	7578395	G	A	Missense_Mutation	0,561111111	360	TRUE	1	Clonal
P-0005434-T01-IM5	Pancreatic neuroendocrine tumor	Metastasis	MLL2	p.R4746W	FALSE	12	49422859	G	A	Missense_Mutation	0,102179837	734	FALSE	0.3	Subclonal
P-0005434-T01-IM5	Pancreatic neuroendocrine tumor	Metastasis	CTCF	p.D608Y	FALSE	16	67663421	G	T	Missense_Mutation	0,355521936	1322	FALSE	1	Clonal
P-0005434-T01-IM5	Pancreatic neuroendocrine tumor	Metastasis	ARID2	p.E74Gfs*11	FALSE	12	46125032	-	G	Frame_Shift_Ins	0,333036509	1123	FALSE	0.97	Clonal
P-0005980-T01-IM5	Pancreatic neuroendocrine tumor	Metastasis	KRAS	p.Q61H	TRUE	12	25380275	T	A	Missense_Mutation	0,530693069	505	TRUE	1	Clonal
P-0005980-T01-IM5	Pancreatic neuroendocrine tumor	Metastasis	IFNGR1	p.V291I	FALSE	6	137519767	C	T	Missense_Mutation	0,170588235	340	FALSE	0.79	Subclonal
P-0005980-T01-IM5	Pancreatic neuroendocrine tumor	Metastasis	KLF4	p.D329Y	FALSE	9	110249690	C	A	Missense_Mutation	0,346534653	303	FALSE	0.92	Clonal
P-0005980-T01-IM5	Pancreatic neuroendocrine tumor	Metastasis	IGF1R	p.S1197Y	FALSE	15	99491805	C	A	Missense_Mutation	0,244155844	385	FALSE	0.89	Clonal
P-0005980-T01-IM5	Pancreatic neuroendocrine tumor	Metastasis	SETD2	p.N604Ifs*18	FALSE	3	47164317	A	-	Frame_Shift_Del	0,600732601	273	TRUE	1	Clonal
P-0006018-T01-IM5	Pancreatic neuroendocrine tumor	Metastasis	MEN1	p.Q171*	FALSE	11	64575521	G	A	Nonsense_Mutation	0,483529412	850	TRUE	0.99	Clonal
P-0006018-T01-IM5	Pancreatic neuroendocrine tumor	Metastasis	DAXX	p.D738Rfs*3	FALSE	6	33286531	-	T	Frame_Shift_Ins	0,425619835	242	TRUE	0.87	Clonal
P-0006382-T02-IM5	Pancreatic neuroendocrine tumor	Metastasis	ARID1A	p.E1766*	FALSE	1	27105685	G	T	Nonsense_Mutation	0,5472103	466	TRUE	1	Clonal
P-0006382-T02-IM5	Pancreatic neuroendocrine tumor	Metastasis	DAXX	p.E454*	FALSE	6	33287893	C	A	Nonsense_Mutation	0,515358362	586	TRUE	1	Clonal
P-0006382-T02-IM5	Pancreatic neuroendocrine tumor	Metastasis	DAXX	p.G250C	FALSE	6	33288804	C	A	Missense_Mutation	0,529247911	359	TRUE	1	Clonal
P-0006382-T02-IM5	Pancreatic neuroendocrine tumor	Metastasis	HRAS	p.A134S	FALSE	11	533503	C	A	Missense_Mutation	0,511744966	596	FALSE	1	Clonal
P-0006382-T02-IM5	Pancreatic neuroendocrine tumor	Metastasis	MEN1	p.E260*	FALSE	11	64575044	C	A	Nonsense_Mutation	0,50955414	471	TRUE	1	Clonal
P-0006382-T02-IM5	Pancreatic neuroendocrine tumor	Metastasis	AKT2	p.S398R	FALSE	19	40741239	G	T	Missense_Mutation	0,335343788	829	FALSE	1	Clonal
P-0006382-T02-IM5	Pancreatic neuroendocrine tumor	Metastasis	CXCR4	p.L86H	FALSE	2	136873241	A	T	Missense_Mutation	0,504310345	696	FALSE	1	Clonal
P-0006382-T02-IM5	Pancreatic neuroendocrine tumor	Metastasis	FGFR4	p.A444S	FALSE	5	176520485	G	T	Missense_Mutation	0,346774194	744	FALSE	1	Clonal
P-0006382-T02-IM5	Pancreatic neuroendocrine tumor	Metastasis	E2F3	p.R216I	FALSE	6	20481578	G	T	Missense_Mutation	0,513711152	547	FALSE	1	Clonal
P-0006382-T02-IM5	Pancreatic neuroendocrine tumor	Metastasis	MLL3	p.S806Y	FALSE	7	151945102	G	T	Missense_Mutation	0,111888112	286	FALSE	0.33	Subclonal
P-0006382-T02-IM5	Pancreatic neuroendocrine tumor	Metastasis	FGFR2	p.G613V	FALSE	10	123256071	C	A	Missense_Mutation	0,496441281	562	FALSE	0.98	Clonal
P-0006382-T02-IM5	Pancreatic neuroendocrine tumor	Metastasis	TSC2	p.W1208C	FALSE	16	2131609	G	T	Missense_Mutation	0,541044776	536	TRUE	1	Clonal
P-0006451-T01-IM5	Pancreatic neuroendocrine tumor	Metastasis	BCOR	p.Y653Tfs*16	FALSE	X	39932646	A	-	Frame_Shift_Del	0,253058596	1553	FALSE	1	Clonal
P-0006494-T01-IM5	Pancreatic neuroendocrine tumor	Metastasis	DAXX	p.Q638*	FALSE	6	33287185	G	A	Nonsense_Mutation	0,705223881	536	TRUE	1	Clonal
P-0006494-T01-IM5	Pancreatic neuroendocrine tumor	Metastasis	BARD1	p.N744S	FALSE	2	215593503	T	C	Missense_Mutation	0,309352518	417	FALSE	0.89	Clonal
P-0006494-T01-IM5	Pancreatic neuroendocrine tumor	Metastasis	BRCA2	p.G2353V	FALSE	13	32929048	G	T	Missense_Mutation	0,197098549	1999	FALSE	0.96	Clonal
P-0006494-T01-IM5	Pancreatic neuroendocrine tumor	Metastasis	TSC2	p.M1?	FALSE	16	2098617	A	G	Translation_Start_Site	0,748878924	892	TRUE	1	Clonal
P-0006494-T01-IM5	Pancreatic neuroendocrine tumor	Metastasis	ARID1A	p.V2041Gfs*58	FALSE	1	27106506	-	G	Frame_Shift_Ins	0,352697095	1205	TRUE	1	Clonal
P-0007022-T01-IM5	Pancreatic neuroendocrine tumor	Metastasis	RNF43	p.M83T	FALSE	17	56492691	A	G	Missense_Mutation	0,303030303	792	FALSE	0.92	Clonal
P-0008043-T01-IM5	Pancreatic neuroendocrine tumor	Metastasis	MEN1	p.E60Sfs*59	FALSE	11	64577408	A	-	Frame_Shift_Del	0,227080395	709	TRUE	0.46	Subclonal
P-0008552-T01-IM5	Pancreatic neuroendocrine tumor	Metastasis	IRS2	p.V798I	FALSE	13	110436009	C	T	Missense_Mutation	0,50308642	324	TRUE	1	Clonal
P-0008552-T01-IM5	Pancreatic neuroendocrine tumor	Metastasis	TP53	p.L257R	TRUE	17	7577511	A	C	Missense_Mutation	0,579022989	696	FALSE	1	Clonal
P-0008552-T01-IM5	Pancreatic neuroendocrine tumor	Metastasis	TP53	p.K321*	FALSE	17	7576885	T	A	Nonsense_Mutation	0,2997543	814	FALSE	1	Clonal
P-0008552-T01-IM5	Pancreatic neuroendocrine tumor	Metastasis	PTPRD	p.T1261M	FALSE	9	8460504	G	A	Missense_Mutation	0,262295082	610	FALSE	0.92	Clonal
P-0008552-T01-IM5	Pancreatic neuroendocrine tumor	Metastasis	RAD52	p.R278*	FALSE	12	1025543	G	A	Nonsense_Mutation	0,230709877	1296	FALSE	1	Clonal
P-0008552-T01-IM5	Pancreatic neuroendocrine tumor	Metastasis	MAX	p.Q15R	FALSE	14	65568283	T	C	Missense_Mutation	0,384397163	705	FALSE	1	Clonal
P-0008552-T01-IM5	Pancreatic neuroendocrine tumor	Metastasis	AURKB	p.E174K	FALSE	17	8110085	C	T	Missense_Mutation	0,309178744	414	FALSE	1	Clonal
P-0010162-T01-IM5	Pancreatic neuroendocrine tumor	Metastasis	BRAF	p.V600E	TRUE	7	140453136	A	T	Missense_Mutation	0,931769723	469	TRUE	1	Clonal
P-0010162-T01-IM5	Pancreatic neuroendocrine tumor	Metastasis	IRS1	p.R411*	FALSE	2	227662224	G	A	Nonsense_Mutation	0,307692308	1157	FALSE	1	Clonal
P-0010162-T01-IM5	Pancreatic neuroendocrine tumor	Metastasis	SETD2	p.E1200*	FALSE	3	47162528	C	A	Nonsense_Mutation	0,951175407	553	TRUE	1	Clonal
P-0010162-T01-IM5	Pancreatic neuroendocrine tumor	Metastasis	MLL2	p.K3551*	FALSE	12	49427939	T	A	Nonsense_Mutation	0,748421053	950	FALSE	1	Clonal
P-0010445-T01-IM5	Pancreatic neuroendocrine tumor	Metastasis	DAXX	p.S737*	FALSE	6	33286533	G	C	Nonsense_Mutation	0,362025316	395	TRUE	0.96	Clonal
P-0010445-T01-IM5	Pancreatic neuroendocrine tumor	Metastasis	TSC2	p.L48Qfs*18	FALSE	16	2100405	TG	-	Frame_Shift_Del	0,384308511	752	TRUE	1	Clonal
P-0010445-T01-IM5	Pancreatic neuroendocrine tumor	Metastasis	MEN1	p.E240del	FALSE	11	64575101	CCT	-	In_Frame_Del	0,333931777	557	TRUE	0.88	Clonal
P-0010490-T01-IM5	Pancreatic neuroendocrine tumor	Metastasis	MEN1	p.L285*	FALSE	11	64574558	G	-	Frame_Shift_Del	0,682539683	882	TRUE	1	Clonal
P-0012117-T01-IM5	Pancreatic neuroendocrine tumor	Metastasis	MAP2K4	p.E92K	FALSE	17	11984728	G	A	Missense_Mutation	0,273781903	431	FALSE	1	Clonal
P-0012117-T01-IM5	Pancreatic neuroendocrine tumor	Metastasis	SMAD4	p.K88_G89del	FALSE	18	48575066	GGAAAG	-	In_Frame_Del	0,335877863	655	TRUE	0.99	Clonal
P-0012117-T01-IM5	Pancreatic neuroendocrine tumor	Metastasis	DAXX	p.L538Wfs*6	FALSE	6	33287485	G	-	Frame_Shift_Del	0,348122867	586	TRUE	1	Clonal
P-0012246-T01-IM5	Pancreatic neuroendocrine tumor	Metastasis	TP53	p.E258K	TRUE	17	7577509	C	T	Missense_Mutation	0,335588633	739	TRUE	1	Clonal
P-0012246-T01-IM5	Pancreatic neuroendocrine tumor	Metastasis	TSC1	p.S282*	FALSE	9	135787737	G	C	Nonsense_Mutation	0,247551202	1123	FALSE	1	Clonal
P-0012246-T01-IM5	Pancreatic neuroendocrine tumor	Metastasis	ARID1A	p.S334*	FALSE	1	27023895	C	A	Nonsense_Mutation	0,438972163	467	FALSE	1	Clonal
P-0012246-T01-IM5	Pancreatic neuroendocrine tumor	Metastasis	BMPR1A	p.F147L	FALSE	10	88659794	T	A	Missense_Mutation	0,224489796	931	FALSE	0.94	Clonal
P-0012786-T01-IM5	Pancreatic neuroendocrine tumor	Metastasis	NTRK1	p.G89D	FALSE	1	156834199	G	A	Missense_Mutation	0,382122905	895	FALSE	1	Clonal
P-0012786-T01-IM5	Pancreatic neuroendocrine tumor	Metastasis	IL10	p.I168T	FALSE	1	206942015	A	G	Missense_Mutation	0,168326693	1004	FALSE	0.95	Clonal
P-0012786-T01-IM5	Pancreatic neuroendocrine tumor	Metastasis	LATS2	p.A324T	FALSE	13	21562949	C	T	Missense_Mutation	0,209705373	577	FALSE	0.97	Clonal
P-0012786-T01-IM5	Pancreatic neuroendocrine tumor	Metastasis	EIF1AX	p.R12G	FALSE	X	20156723	T	C	Missense_Mutation	0,213978495	930	FALSE	1	Clonal
P-0013655-T01-IM5	Pancreatic neuroendocrine tumor	Metastasis	KDM5A	p.P914L	FALSE	12	427428	G	A	Missense_Mutation	0,373578303	1143	FALSE	1	Clonal
P-0013655-T01-IM5	Pancreatic neuroendocrine tumor	Metastasis	TSC2	p.G20Ffs*11	FALSE	16	2098674	GGACTGGGAACACCGA	TTCTG	Frame_Shift_Del	0,451169188	727	TRUE	1	Clonal
P-0013655-T01-IM5	Pancreatic neuroendocrine tumor	Metastasis	FBXW7	p.Q220*	FALSE	4	153268150	G	A	Nonsense_Mutation	0,213644524	1114	FALSE	1	Clonal
P-0013655-T01-IM5	Pancreatic neuroendocrine tumor	Metastasis	RAD50	p.Q854E	FALSE	5	131940533	C	G	Missense_Mutation	0,400437637	914	FALSE	1	Clonal
P-0013655-T01-IM5	Pancreatic neuroendocrine tumor	Metastasis	NSD1	p.M1094V	FALSE	5	176638680	A	G	Missense_Mutation	0,186540732	847	FALSE	1	Clonal
P-0013655-T01-IM5	Pancreatic neuroendocrine tumor	Metastasis	INHBA	p.S237Y	FALSE	7	41729819	G	T	Missense_Mutation	0,329334787	917	FALSE	1	Clonal
P-0014098-T01-IM5	Pancreatic neuroendocrine tumor	Metastasis	VHL	p.R167W	FALSE	3	10191506	C	T	Missense_Mutation	0,627586207	435	TRUE	1	Clonal

Table S2

Sample ID	Cancer subtype	Sample type	Symbol	Aminoacid change	Hotspot	Chromosome	Position	Reference	Alternate	Mutation type	Tumor MAF	Tumor DP	LOH	Cancer cell fraction	Clonal status
P-0014564-T01-IM6	Pancreatic neuroendocrine tumor	Metastasis	EGFR	p.V441dup	FALSE	7	55227856	-	GTT	In_Frame_Ins	0,106918239	318	FALSE	0.44	Subclonal
P-0015140-T01-IM6	Pancreatic neuroendocrine tumor	Metastasis	TP53	p.R175H	TRUE	17	7578406	C	T	Missense_Mutation	0,1831972	857	FALSE	0.53	Subclonal
P-0015140-T01-IM6	Pancreatic neuroendocrine tumor	Metastasis	PTEN	p.N323Kfs*2	FALSE	10	89720811	-	A	Frame_Shift_Ins	0,465608466	189	TRUE	0.88	Clonal
P-0015140-T01-IM6	Pancreatic neuroendocrine tumor	Metastasis	KRAS	p.V14I	FALSE	12	25398279	C	T	Missense_Mutation	0,337443946	892	FALSE	0.98	Clonal
P-0015140-T01-IM6	Pancreatic neuroendocrine tumor	Metastasis	SETD2	p.Y469H	FALSE	3	47164721	A	G	Missense_Mutation	0,56302521	357	TRUE	1	Clonal
P-0015140-T01-IM6	Pancreatic neuroendocrine tumor	Metastasis	SMARCA4	p.R513Q	FALSE	19	11105622	G	A	Missense_Mutation	0,099337748	1057	FALSE	0.29	Subclonal
P-0015140-T01-IM6	Pancreatic neuroendocrine tumor	Metastasis	ERCC2	p.A172T	FALSE	19	45868176	C	T	Missense_Mutation	0,364305428	1087	FALSE	1	Clonal
P-0015140-T01-IM6	Pancreatic neuroendocrine tumor	Metastasis	FLCN	p.V400I	FALSE	17	17119796	C	T	Missense_Mutation	0,348348348	999	FALSE	1	Clonal
P-0015140-T01-IM6	Pancreatic neuroendocrine tumor	Metastasis	MED12	p.A1383T	FALSE	X	70351950	G	A	Missense_Mutation	0,334365325	646	FALSE	1	Clonal
P-0015140-T01-IM6	Pancreatic neuroendocrine tumor	Metastasis	MTOR	p.Q2082L	FALSE	1	11187173	T	A	Missense_Mutation	0,538860104	579	FALSE	1	Clonal
P-0015140-T01-IM6	Pancreatic neuroendocrine tumor	Metastasis	NOTCH2	p.A1654T	FALSE	1	120465301	C	T	Missense_Mutation	0,571428571	420	FALSE	1	Clonal
P-0015140-T01-IM6	Pancreatic neuroendocrine tumor	Metastasis	BMPR1A	p.R406H	FALSE	10	88681327	G	A	Missense_Mutation	0,561837456	283	FALSE	1	Clonal
P-0015140-T01-IM6	Pancreatic neuroendocrine tumor	Metastasis	MEN1	p.L381P	FALSE	11	64573165	A	G	Missense_Mutation	0,579391892	592	TRUE	1	Clonal
P-0015140-T01-IM6	Pancreatic neuroendocrine tumor	Metastasis	CREBBP	p.E1278K	FALSE	16	3799632	C	T	Missense_Mutation	0,509202454	489	TRUE	0.96	Clonal
P-0015140-T01-IM6	Pancreatic neuroendocrine tumor	Metastasis	MSH6	p.S28*	FALSE	2	48010455	C	A	Nonsense_Mutation	0,572289157	498	FALSE	1	Clonal
P-0015140-T01-IM6	Pancreatic neuroendocrine tumor	Metastasis	SETD2	p.A2009P	FALSE	3	47125245	C	G	Missense_Mutation	0,579295154	454	TRUE	1	Clonal
P-0015140-T01-IM6	Pancreatic neuroendocrine tumor	Metastasis	DAXX	p.E465Gfs*63	FALSE	6	33287859	T	-	Frame_Shift_Del	0,579908676	657	TRUE	1	Clonal
P-0015140-T01-IM6	Pancreatic neuroendocrine tumor	Metastasis	PTP4A1	p.R138C	FALSE	6	64289969	C	T	Missense_Mutation	0,541025641	390	FALSE	1	Clonal
P-0015140-T01-IM6	Pancreatic neuroendocrine tumor	Metastasis	RXRA	p.R164C	FALSE	9	137300845	C	T	Missense_Mutation	0,116531165	1107	FALSE	0.34	Subclonal
P-0015140-T01-IM6	Pancreatic neuroendocrine tumor	Metastasis	SDHA	p.S346P	FALSE	5	233732	TCC	CCG	Missense_Mutation	0,195086705	692	FALSE	0.56	Subclonal
P-0015232-T01-IM6	Pancreatic neuroendocrine tumor	Metastasis	ARID1A	p.Q789*	FALSE	1	27088756	C	T	Nonsense_Mutation	0,503863988	647	TRUE	1	Clonal
P-0015232-T01-IM6	Pancreatic neuroendocrine tumor	Metastasis	PALB2	p.L484I	FALSE	16	23646417	A	T	Missense_Mutation	0,373725934	883	FALSE	1	Clonal
P-0015232-T01-IM6	Pancreatic neuroendocrine tumor	Metastasis	FAM175A	p.I61T	FALSE	4	84397829	A	G	Missense_Mutation	0,538461538	481	FALSE	1	Clonal
P-0015340-T01-IM6	Pancreatic neuroendocrine tumor	Metastasis	PTEN	p.Y65*	FALSE	10	89685300	C	A	Nonsense_Mutation	0,362745098	306	TRUE	1	Clonal
P-0015340-T01-IM6	Pancreatic neuroendocrine tumor	Metastasis	MEN1	p.I85Sfs*33	FALSE	11	64577330	AGAC	-	Frame_Shift_Del	0,360377358	530	TRUE	1	Clonal
P-0015340-T01-IM6	Pancreatic neuroendocrine tumor	Metastasis	DAXX	p.D738V	FALSE	6	33286530	T	A	Missense_Mutation	0,392014519	551	TRUE	1	Clonal
P-0015340-T01-IM6	Pancreatic neuroendocrine tumor	Metastasis	AXIN1	p.G295R	FALSE	16	364679	C	T	Missense_Mutation	0,193116635	523	FALSE	1	Clonal
P-0015914-T01-IM6	Pancreatic neuroendocrine tumor	Metastasis	FGFR1	p.K687E	FALSE	8	38272308	T	C	Missense_Mutation	0,097103918	587	FALSE	0.32	Subclonal
P-0015914-T01-IM6	Pancreatic neuroendocrine tumor	Metastasis	ERRFI1	p.S58P	FALSE	1	8075398	A	G	Missense_Mutation	0,068085106	235	FALSE	0.23	Subclonal
P-0015914-T01-IM6	Pancreatic neuroendocrine tumor	Metastasis	GLI1	p.H157R	FALSE	12	57858974	A	G	Missense_Mutation	0,217453505	699	FALSE	0.72	Subclonal
P-0015914-T01-IM6	Pancreatic neuroendocrine tumor	Metastasis	ERCC4	p.T221I	FALSE	16	14021962	C	T	Missense_Mutation	0,296482412	398	FALSE	0.98	Clonal
P-0015914-T01-IM6	Pancreatic neuroendocrine tumor	Metastasis	CIC	p.P574L	FALSE	19	42794641	C	T	Missense_Mutation	0,076923077	637	FALSE	0.26	Subclonal
P-0015914-T01-IM6	Pancreatic neuroendocrine tumor	Metastasis	SETD2	p.V1820A	FALSE	3	47125811	A	G	Missense_Mutation	0,317567568	296	FALSE	1	Clonal
P-0015914-T01-IM6	Pancreatic neuroendocrine tumor	Metastasis	FAT1	p.D1293G	FALSE	4	187557833	T	C	Missense_Mutation	0,245283019	477	FALSE	0.81	Subclonal
P-0015914-T01-IM6	Pancreatic neuroendocrine tumor	Metastasis	LATS1	p.Y808H	FALSE	6	150001182	A	G	Missense_Mutation	0,300275482	363	FALSE	1	Clonal
P-0015914-T01-IM6	Pancreatic neuroendocrine tumor	Metastasis	MYC	p.P75S	FALSE	8	128750686	C	T	Missense_Mutation	0,07606679	539	FALSE	0.25	Subclonal
P-0015914-T01-IM6	Pancreatic neuroendocrine tumor	Metastasis	KLF4	p.V282A	FALSE	9	110249830	A	G	Missense_Mutation	0,299236641	655	FALSE	0.99	Clonal
P-0015914-T01-IM6	Pancreatic neuroendocrine tumor	Metastasis	NOTCH1	p.E556G	FALSE	9	139410435	T	C	Missense_Mutation	0,236156352	614	FALSE	0.78	Subclonal
P-0015935-T01-IM6	Pancreatic neuroendocrine tumor	Metastasis	BCOR	p.N1459S	TRUE	X	39921444	T	C	Missense_Mutation	0,638497653	426	FALSE	1	Clonal
P-0015935-T01-IM6	Pancreatic neuroendocrine tumor	Metastasis	TP53	p.I255T	TRUE	17	7577517	A	G	Missense_Mutation	0,648590022	461	TRUE	1	Clonal
P-0015935-T01-IM6	Pancreatic neuroendocrine tumor	Metastasis	JAK1	p.N28S	FALSE	1	65349082	T	C	Missense_Mutation	0,255544841	1037	FALSE	0.94	Clonal
P-0015935-T01-IM6	Pancreatic neuroendocrine tumor	Metastasis	ATM	p.X412_splice	FALSE	11	108119831	T	C	Splice_Site	0,241650295	509	FALSE	0.89	Clonal
P-0015935-T01-IM6	Pancreatic neuroendocrine tumor	Metastasis	PRKD1	p.N248I	FALSE	14	30108064	T	A	Missense_Mutation	0,236375575	1523	FALSE	1	Clonal
P-0015935-T01-IM6	Pancreatic neuroendocrine tumor	Metastasis	ERCC4	p.M451V	FALSE	16	14029140	A	G	Missense_Mutation	0,25	640	FALSE	0.92	Clonal
P-0015935-T01-IM6	Pancreatic neuroendocrine tumor	Metastasis	CDH1	p.R524L	FALSE	16	68853188	G	T	Missense_Mutation	0,279299014	913	FALSE	1	Clonal
P-0015935-T01-IM6	Pancreatic neuroendocrine tumor	Metastasis	SPOP	p.L76S	FALSE	17	47696721	A	G	Missense_Mutation	0,250295159	847	FALSE	0.92	Clonal
P-0015935-T01-IM6	Pancreatic neuroendocrine tumor	Metastasis	ERBB4	p.H856Q	FALSE	2	212295745	A	T	Missense_Mutation	0,391420912	746	TRUE	1	Clonal
P-0015935-T01-IM6	Pancreatic neuroendocrine tumor	Metastasis	RUNX1	p.S436P	FALSE	21	36164569	A	G	Missense_Mutation	0,384496124	645	FALSE	1	Clonal
P-0015935-T01-IM6	Pancreatic neuroendocrine tumor	Metastasis	CSF1R	p.N412S	FALSE	5	149449829	T	C	Missense_Mutation	0,259484067	1318	FALSE	0.95	Clonal
P-0016483-T01-IM6	Pancreatic neuroendocrine tumor	Metastasis	KMT2D	p.E1816*	FALSE	12	49437439	C	A	Nonsense_Mutation	0,310743802	605	FALSE	1	Clonal
P-0016483-T01-IM6	Pancreatic neuroendocrine tumor	Metastasis	ZFHX3	p.E476K	FALSE	16	72992619	C	T	Missense_Mutation	0,34841629	442	FALSE	1	Clonal
P-0016483-T01-IM6	Pancreatic neuroendocrine tumor	Metastasis	EPHA7	p.G741R	FALSE	6	93965707	C	T	Missense_Mutation	0,106448311	977	FALSE	0.34	Subclonal
P-0016585-T01-IM6	Pancreatic neuroendocrine tumor	Metastasis	MEN1	p.E30Dfs*87	FALSE	11	64577492	-	G	Frame_Shift_Ins	0,858880779	411	TRUE	0.94	Clonal
P-0016585-T01-IM6	Pancreatic neuroendocrine tumor	Metastasis	POLE	p.M1754V	FALSE	12	133218351	T	C	Missense_Mutation	0,458870168	1009	FALSE	0.96	Clonal
P-0016585-T01-IM6	Pancreatic neuroendocrine tumor	Metastasis	TSC2	p.Y130*	FALSE	16	2104350	C	A	Nonsense_Mutation	0,918300654	612	TRUE	1	Clonal
P-0016585-T01-IM6	Pancreatic neuroendocrine tumor	Metastasis	DAXX	p.T435Mfs*92	FALSE	6	33287946	TCTG	-	Frame_Shift_Del	0,919101124	445	TRUE	1	Clonal
P-0016993-T01-IM6	Pancreatic neuroendocrine tumor	Metastasis	IGF1R	p.Q212*	FALSE	15	99251330	C	T	Nonsense_Mutation	0,378881988	644	FALSE	1	Clonal
P-0018232-T01-IM6	Pancreatic neuroendocrine tumor	Metastasis	PTEN	p.Y88*	FALSE	10	89692780	T	G	Nonsense_Mutation	0,052877138	643	TRUE	1	Clonal
P-0018232-T01-IM6	Pancreatic neuroendocrine tumor	Metastasis	SUFU	p.G378E	FALSE	10	104375135	G	A	Missense_Mutation	0,143048128	748	FALSE	1	Clonal
P-0018232-T01-IM6	Pancreatic neuroendocrine tumor	Metastasis	PGR	p.E146_T157del	FALSE	11	100999331	GGTGGCGGGGGCAGCCGGTGGATCTTCGGGAAGTTC	-	In_Frame_Del	0,186230248	886	FALSE	0.37	Subclonal
P-0018232-T01-IM6	Pancreatic neuroendocrine tumor	Metastasis	MGA	p.T2231A	FALSE	15	42042496	A	G	Missense_Mutation	0,126168224	642	FALSE	0.25	Subclonal
P-0018232-T01-IM6	Pancreatic neuroendocrine tumor	Metastasis	TRAF7	p.D631N	FALSE	16	2226278	G	A	Missense_Mutation	0,111441308	673	FALSE	0.22	Subclonal
P-0018232-T01-IM6	Pancreatic neuroendocrine tumor	Metastasis	SLX4	p.X561_splice	FALSE	16	3647379	C	T	Splice_Site	0,132701422	422	FALSE	0.26	Subclonal
P-0018232-T01-IM6	Pancreatic neuroendocrine tumor	Metastasis	ZFHX3	p.P3317L	FALSE	16	72822225	G	A	Missense_Mutation	0,153153153	777	FALSE	0.3	Subclonal
P-0018232-T01-IM6	Pancreatic neuroendocrine tumor	Metastasis	NCOR1	p.K1106R	FALSE	17	15983805	T	C	Missense_Mutation	0,13583815	1038	FALSE	0.4	Subclonal
P-0018232-T01-IM6	Pancreatic neuroendocrine tumor	Metastasis	RNF43	p.P112L	FALSE	17	56448312	G	A	Missense_Mutation	0,108734403	1122	FALSE	0.32	Subclonal
P-0018232-T01-IM6	Pancreatic neuroendocrine tumor	Metastasis	JAK3	p.P689L	FALSE	19	17945794	G	A	Missense_Mutation	0,109947644	955	FALSE	0.33	Subclonal
P-0018232-T01-IM6	Pancreatic neuroendocrine tumor	Metastasis	SETD2	p.P2527L	FALSE	3	47058698	G	A	Missense_Mutation	0,168253968	630	TRUE	0.33	Subclonal
P-0018232-T01-IM6	Pancreatic neuroendocrine tumor	Metastasis	FLT4	p.X951_splice	FALSE	5	180045922	T	C	Splice_Site	0,110544218	588	FALSE	0.33	Subclonal
P-0018232-T01-IM6	Pancreatic neuroendocrine tumor	Metastasis	DAXX	p.G519R	FALSE	6	33287542	C	T	Missense_Mutation	0,170610212	803	TRUE	0.34	Subclonal
P-0018232-T01-IM6	Pancreatic neuroendocrine tumor	Metastasis	DAXX	p.Q308*	FALSE	6	33288630	G	A	Nonsense_Mutation	0,589041096	365	TRUE	1	Clonal
P-0018232-T01-IM6	Pancreatic neuroendocrine tumor	Metastasis	LATS1	p.P491S	FALSE	6	150004754	G	A	Missense_Mutation	0,182857143	525	FALSE	0.36	Subclonal
P-0018232-T01-IM6	Pancreatic neuroendocrine tumor	Metastasis	MEN1	p.T305_K309del	FALSE	11	64574483	CTTGTGGTAGAGGGTG	A	In_Frame_Del	0,487767584	654	TRUE	0.96	Clonal
P-0019281-T01-IM6	Pancreatic neuroendocrine tumor	Metastasis	PTEN	p.T319*	FALSE	10	89720799	TACT	-	Frame_Shift_Del	0,459677419	124	TRUE	0.87	Clonal
P-0019281-T01-IM6	Pancreatic neuroendocrine tumor	Metastasis	TP53	p.F212Sfs*3	FALSE	17	7578213	AA	-	Frame_Shift_Del	0,523642732	571	TRUE	0.99	Clonal
P-0019281-T01-IM6	Pancreatic neuroendocrine tumor	Metastasis	MEN1	p.K120del	FALSE	11	64577222	CTT	-	In_Frame_Del	0,534552846	492	TRUE	1	Clonal
P-0019281-T01-IM6	Pancreatic neuroendocrine tumor	Metastasis	PPM1D	p.C603Ffs*21	FALSE	17	58740898	TGTT	-	Frame_Shift_Del	0,234887737	579	FALSE	0.68	Subclonal
P-0019281-T01-IM6	Pancreatic neuroendocrine tumor	Metastasis	BCOR	p.K1137Sfs*4	FALSE	X	39923680	TT	-	Frame_Shift_Del	0,36967632	587	FALSE	1	Clonal
P-0019281-T01-IM6	Pancreatic neuroendocrine tumor	Metastasis	TSC2	p.F124del	FALSE	16	2104325	TCT	-	In_Frame_Del	0,569196429	448	TRUE	1	Clonal
P-0019281-T01-IM6	Pancreatic neuroendocrine tumor	Metastasis	RAF1	p.G370del	FALSE	3	12633289	CTC	-	In_Frame_Del	0,527559055	381	FALSE	0.99	Clonal
P-0019281-T01-IM6	Pancreatic neuroendocrine tumor	Metastasis	DAXX	p.P689Qfs*51	FALSE	6	33286872	GA	-	Frame_Shift_Del	0,52688172	465	TRUE	0.99	Clonal

Table S2

Sample ID	Cancer subtype	Sample type	Symbol	Aminoacid change	Hotspot	Chromosome	Position	Reference	Alternate	Mutation type	Tumor MAF	Tumor DP	LOH	Cancer cell fraction	Clonal status
P-0019646-T01-IM6	Pancreatic neuroendocrine tumor	Metastasis	SMAD4	p.G352E	FALSE	18	48591892	G	A	Missense_Mutation	0,625577812	649	TRUE	1	Clonal
P-0019646-T01-IM6	Pancreatic neuroendocrine tumor	Metastasis	KRAS	p.A146V	TRUE	12	25378561	G	A	Missense_Mutation	0,298091043	681	TRUE	0.94	Clonal
P-0019646-T01-IM6	Pancreatic neuroendocrine tumor	Metastasis	MEN1	p.Q96Rfs*23	FALSE	11	64577296	G	-	Frame_Shift_Del	0,627534685	937	FALSE	1	Clonal
P-0019753-T01-IM6	Pancreatic neuroendocrine tumor	Metastasis	PTEN	p.K267Rfs*9	FALSE	10	89717770	A	-	Frame_Shift_Del	0,369620253	395	TRUE	1	Clonal
P-0019753-T01-IM6	Pancreatic neuroendocrine tumor	Metastasis	MEN1	p.G161V	FALSE	11	64575550	C	A	Missense_Mutation	0,400702988	569	TRUE	1	Clonal
P-0019753-T01-IM6	Pancreatic neuroendocrine tumor	Metastasis	PIK3R1	p.L165F	FALSE	5	67569832	C	T	Missense_Mutation	0,255965293	461	FALSE	0.95	Clonal
P-0020526-T01-IM6	Pancreatic neuroendocrine tumor	Metastasis	KRAS	p.G12D	TRUE	12	25398284	C	T	Missense_Mutation	0,571799308	1156	FALSE	1	Clonal
P-0020526-T01-IM6	Pancreatic neuroendocrine tumor	Metastasis	TP53	p.G245S	TRUE	17	7577548	C	T	Missense_Mutation	0,672683514	831	TRUE	1	Clonal
P-0020939-T01-IM6	Pancreatic neuroendocrine tumor	Metastasis	MEN1	p.I85Sfs*33	FALSE	11	64577330	AGAC	-	Frame_Shift_Del	0,818740399	651	TRUE	1	Clonal
P-0020939-T01-IM6	Pancreatic neuroendocrine tumor	Metastasis	DAXX	p.S102L	FALSE	6	33289247	G	A	Missense_Mutation	0,85440613	522	TRUE	1	Clonal
P-0020939-T01-IM6	Pancreatic neuroendocrine tumor	Metastasis	PIK3C2G	p.P176L	FALSE	12	18435542	C	T	Missense_Mutation	0,290748899	454	FALSE	0.65	Subclonal
P-0020939-T01-IM6	Pancreatic neuroendocrine tumor	Metastasis	MET	p.A1207V	FALSE	7	116422139	C	T	Missense_Mutation	0,437381404	1054	FALSE	0.98	Clonal
P-0022435-T01-IM6	Pancreatic neuroendocrine tumor	Metastasis	KMT2C	p.M3101V	FALSE	7	151871289	T	C	Missense_Mutation	0,059453032	841	FALSE	0.15	Subclonal
P-0022639-T01-IM6	Pancreatic neuroendocrine tumor	Metastasis	TP53	p.R273C	TRUE	17	7577121	G	A	Missense_Mutation	0,814241486	646	TRUE	1	Clonal
P-0022639-T01-IM6	Pancreatic neuroendocrine tumor	Metastasis	MTOR	p.A893S	FALSE	1	11291084	CC	AA	Missense_Mutation	0,109782609	920	FALSE	0.26	Subclonal
P-0022639-T01-IM6	Pancreatic neuroendocrine tumor	Metastasis	KMT2D	p.Q4210*	FALSE	12	49425860	G	A	Nonsense_Mutation	0,42	1000	FALSE	1	Clonal
P-0022639-T01-IM6	Pancreatic neuroendocrine tumor	Metastasis	POLE	p.R1136W	FALSE	12	133233988	G	A	Missense_Mutation	0,414492754	1035	FALSE	0.98	Clonal
P-0022639-T01-IM6	Pancreatic neuroendocrine tumor	Metastasis	APC	p.T1082Rfs*30	FALSE	5	112174532	AGCACTGATGATAAACACCTCAAGTTCCAACCACATTTTGGAC	-	Frame_Shift_Del	0,72826087	276	TRUE	1	Clonal
P-0022720-T01-IM6	Pancreatic neuroendocrine tumor	Metastasis	TP53	p.E286K	TRUE	17	7577082	C	T	Missense_Mutation	0,205499276	691	FALSE	1	Clonal
P-0022720-T01-IM6	Pancreatic neuroendocrine tumor	Metastasis	MEN1	p.L83Rfs*36	FALSE	11	64577334	A	-	Frame_Shift_Del	0,211505922	591	FALSE	1	Clonal
P-0022746-T01-IM6	Pancreatic neuroendocrine tumor	Metastasis	HLA-B	p.G40S	FALSE	6	31324690	C	T	Missense_Mutation	0,285714286	154	FALSE	0.57	Subclonal
P-0022746-T01-IM6	Pancreatic neuroendocrine tumor	Metastasis	HLA-B	p.R45H	FALSE	6	31324674	C	T	Missense_Mutation	0,169014085	142	FALSE	0.34	Subclonal
P-0022746-T01-IM6	Pancreatic neuroendocrine tumor	Metastasis	TSC2	p.W703R	FALSE	16	2122251	T	A	Missense_Mutation	0,516791045	536	TRUE	1	Clonal
P-0022746-T01-IM6	Pancreatic neuroendocrine tumor	Metastasis	FAT1	p.Y2014S	FALSE	4	187541699	T	G	Missense_Mutation	0,317507418	337	FALSE	0.96	Clonal
P-0022746-T01-IM6	Pancreatic neuroendocrine tumor	Metastasis	HIST1H3B	p.R135I	FALSE	6	26031885	C	A	Missense_Mutation	0,253658537	205	FALSE	0.51	Subclonal
P-0022746-T01-IM6	Pancreatic neuroendocrine tumor	Metastasis	HLA-B	p.A235T	FALSE	6	31323286	C	T	Missense_Mutation	0,073033708	178	FALSE	0.15	Subclonal
P-0022746-T01-IM6	Pancreatic neuroendocrine tumor	Metastasis	DAXX	p.Q77*	FALSE	6	33289323	G	A	Nonsense_Mutation	0,46460177	226	TRUE	0.93	Clonal
P-0023837-T01-IM6	Pancreatic neuroendocrine tumor	Metastasis	MEN1	p.I85Sfs*33	FALSE	11	64577330	AGAC	-	Frame_Shift_Del	0,431428571	350	TRUE	1	Clonal
P-0023837-T01-IM6	Pancreatic neuroendocrine tumor	Metastasis	NTRK1	p.V524F	FALSE	1	156845940	G	T	Missense_Mutation	0,45646438	379	FALSE	1	Clonal
P-0023837-T01-IM6	Pancreatic neuroendocrine tumor	Metastasis	DAXX	p.H711Ifs*55	FALSE	6	33286806	G	-	Frame_Shift_Del	0,410569106	246	TRUE	0.96	Clonal
P-0024354-T01-IM6	Pancreatic neuroendocrine tumor	Metastasis	KRAS	p.G12D	TRUE	12	25398284	C	T	Missense_Mutation	0,187165775	374	FALSE	0.91	Clonal
P-0024354-T01-IM6	Pancreatic neuroendocrine tumor	Metastasis	TP53	p.S241Y	TRUE	17	7577559	G	T	Missense_Mutation	0,207253886	193	FALSE	0.58	Subclonal
P-0024465-T01-IM6	Pancreatic neuroendocrine tumor	Metastasis	ARID1B	p.R1624*	FALSE	6	157522598	C	T	Nonsense_Mutation	0,420664207	542	FALSE	1	Clonal
P-0024465-T01-IM6	Pancreatic neuroendocrine tumor	Metastasis	RUNX1	p.R201*	FALSE	21	36231783	G	A	Nonsense_Mutation	0,399046105	629	FALSE	1	Clonal
P-0024465-T01-IM6	Pancreatic neuroendocrine tumor	Metastasis	ERBB3	p.G325R	FALSE	12	56482425	G	A	Missense_Mutation	0,389830508	531	FALSE	1	Clonal
P-0024465-T01-IM6	Pancreatic neuroendocrine tumor	Metastasis	ARID1A	p.Q1519*	FALSE	1	27101273	C	T	Nonsense_Mutation	0,410447761	536	FALSE	1	Clonal
P-0024465-T01-IM6	Pancreatic neuroendocrine tumor	Metastasis	KMT2C	p.R2139*	FALSE	7	151878530	G	A	Nonsense_Mutation	0,408928571	560	FALSE	1	Clonal
P-0024465-T01-IM6	Pancreatic neuroendocrine tumor	Metastasis	ARID1A	p.F1941Yfs*39	FALSE	1	27106211	TTGGCATTAGCCCAGCACAGAGCCACCGGAACATCAAGATCCTAGAGGACG AACCCCACAGTAAGGATGAGACCCCACTGTGTACCCTTCTGGACTGGCAGG ATTCTCTTGCCAAGCGCTGCGTCTGTGTGTCCAATACCATTCGAAGCCTGTC ATTTGTGCCAGGCAATGACTTTGAGATGTCCAAACACCCAGGGCTGCTGCTC ATCCTGGGCAAGCTGATCCTGCTGCACCACAAGCACCCAGAACGGAAGCAG GCACCACTAACTTATGAAAAGGAGGAGGAACAGGACCAAGGGGTGAGCTGC AACAAAGTGGAGTGGTGGTGGGACTGCTTGGAGATGCTCCGGGAAAAC	-	Frame_Shift_Del	0,403614458	498	FALSE	1	Clonal
P-0024465-T01-IM6	Pancreatic neuroendocrine tumor	Metastasis	DOT1L	p.E1417K	FALSE	19	2226769	G	A	Missense_Mutation	0,713091922	359	TRUE	1	Clonal
P-0024465-T01-IM6	Pancreatic neuroendocrine tumor	Metastasis	ERCC3	p.R234Q	FALSE	2	128047034	C	T	Missense_Mutation	0,419161677	501	FALSE	1	Clonal
P-0024465-T01-IM6	Pancreatic neuroendocrine tumor	Metastasis	FBXW7	p.Q508*	FALSE	4	153247280	G	A	Nonsense_Mutation	0,42557652	477	FALSE	1	Clonal
P-0024465-T01-IM6	Pancreatic neuroendocrine tumor	Metastasis	APC	p.W157Vfs*10	FALSE	5	112111372	TG	-	Frame_Shift_Del	0,367256637	226	FALSE	1	Clonal
P-0024465-T01-IM6	Pancreatic neuroendocrine tumor	Metastasis	APC	p.Y799Mfs*21	FALSE	5	112173685	T	-	Frame_Shift_Del	0,432276657	347	FALSE	1	Clonal
P-0024465-T01-IM6	Pancreatic neuroendocrine tumor	Metastasis	MET	p.S701G	FALSE	7	116397827	A	G	Missense_Mutation	0,422969188	357	FALSE	1	Clonal
P-0024508-T01-IM6	Pancreatic neuroendocrine tumor	Metastasis	MEN1	p.D70Tfs*49	FALSE	11	64577375	G	-	Frame_Shift_Del	0,331550802	935	FALSE	0.92	Clonal
P-0024508-T01-IM6	Pancreatic neuroendocrine tumor	Metastasis	ATM	p.P2271R	FALSE	11	108196789	C	G	Missense_Mutation	0,389150943	424	FALSE	1	Clonal
P-0024569-T01-IM6	Pancreatic neuroendocrine tumor	Metastasis	KRAS	p.G12D	TRUE	12	25398284	C	T	Missense_Mutation	0,591025641	780	FALSE	1	Clonal
P-0024569-T01-IM6	Pancreatic neuroendocrine tumor	Metastasis	TP53	p.R306*	FALSE	17	7577022	G	A	Nonsense_Mutation	0,513779528	508	FALSE	1	Clonal
P-0024569-T01-IM6	Pancreatic neuroendocrine tumor	Metastasis	KMT2D	p.G3670V	FALSE	12	49427479	C	A	Missense_Mutation	0,360497238	724	FALSE	1	Clonal
P-0024569-T01-IM6	Pancreatic neuroendocrine tumor	Metastasis	BRCA1	p.I986M	FALSE	17	41244590	G	C	Missense_Mutation	0,505681818	352	FALSE	1	Clonal
P-0024569-T01-IM6	Pancreatic neuroendocrine tumor	Metastasis	PDGFRB	p.D575N	FALSE	5	149505092	C	T	Missense_Mutation	0,269264836	1129	FALSE	1	Clonal
P-0024953-T01-IM6	Pancreatic neuroendocrine tumor	Metastasis	IRS2	p.P954L	FALSE	13	110435540	G	A	Missense_Mutation	0,5917603	267	TRUE	1	Clonal
P-0024953-T01-IM6	Pancreatic neuroendocrine tumor	Metastasis	TSC2	p.P1389Afs*22	FALSE	16	2134386	CTCCCGAG	-	Frame_Shift_Del	0,5	450	TRUE	1	Clonal
P-0025679-T01-IM6	Pancreatic neuroendocrine tumor	Metastasis	DAXX	p.R318*	FALSE	6	33288600	G	A	Nonsense_Mutation	0,225988701	531	FALSE	0.96	Clonal
P-0025679-T01-IM6	Pancreatic neuroendocrine tumor	Metastasis	CDKN2C	p.D67H	FALSE	1	51439634	G	C	Missense_Mutation	0,277456647	519	FALSE	1	Clonal
P-0025679-T01-IM6	Pancreatic neuroendocrine tumor	Metastasis	MEN1	p.W126*	FALSE	11	64577204	C	-	Frame_Shift_Del	0,234273319	922	FALSE	1	Clonal
P-0025679-T01-IM6	Pancreatic neuroendocrine tumor	Metastasis	TSC2	p.A822Ffs*4	FALSE	16	2124308	GGCGCTGCCT	-	Frame_Shift_Del	0,227712137	931	FALSE	0.97	Clonal
P-0028691-T01-IM6	Pancreatic neuroendocrine tumor	Metastasis	KRAS	p.G12V	TRUE	12	25398284	C	A	Missense_Mutation	0,642176871	735	FALSE	1	Clonal
P-0028691-T01-IM6	Pancreatic neuroendocrine tumor	Metastasis	TP53	p.K132R	TRUE	17	7578535	T	C	Missense_Mutation	0,801801802	1221	TRUE	1	Clonal
P-0028691-T01-IM6	Pancreatic neuroendocrine tumor	Metastasis	RB1	p.S648Tfs*4	FALSE	13	49030465	TT	-	Frame_Shift_Del	0,770573566	401	TRUE	1	Clonal
P-0028691-T01-IM6	Pancreatic neuroendocrine tumor	Metastasis	SMYD3	p.L417R	FALSE	1	245912902	A	C	Missense_Mutation	0,257709251	454	FALSE	1	Clonal
P-0028691-T01-IM6	Pancreatic neuroendocrine tumor	Metastasis	RPS6KA4	p.V514L	FALSE	11	64137029	G	C	Missense_Mutation	0,369472183	701	FALSE	1	Clonal
P-0028691-T01-IM6	Pancreatic neuroendocrine tumor	Metastasis	IRS2	p.S606P	FALSE	13	110436585	A	G	Missense_Mutation	0,214953271	1391	FALSE	0.78	Subclonal
P-0028691-T01-IM6	Pancreatic neuroendocrine tumor	Metastasis	PDGFRB	p.P427L	FALSE	5	149510189	G	A	Missense_Mutation	0,244798042	817	FALSE	1	Clonal
P-0029599-T01-IM6	Pancreatic neuroendocrine tumor	Metastasis	STK11	p.X97_splice	FALSE	19	1207203	G	T	Splice_Site	0,919925512	537	FALSE	1	Clonal
P-0029599-T01-IM6	Pancreatic neuroendocrine tumor	Metastasis	ATM	p.R337C	TRUE	11	108117798	C	T	Missense_Mutation	0,889908257	218	TRUE	1	Clonal
P-0029749-T01-IM6	Pancreatic neuroendocrine tumor	Metastasis	MEN1	p.I85Sfs*33	FALSE	11	64577330	AGAC	-	Frame_Shift_Del	0,550070522	709	TRUE	0.86	Clonal
P-0029749-T01-IM6	Pancreatic neuroendocrine tumor	Metastasis	TSC2	p.A1546T	FALSE	16	2135297	G	A	Missense_Mutation	0,648834019	729	TRUE	1	Clonal
P-0029749-T01-IM6	Pancreatic neuroendocrine tumor	Metastasis	DAXX	p.D740Lfs*27	FALSE	6	33286525	-	AA	Frame_Shift_Ins	0,569506726	446	TRUE	0.89	Clonal
P-0029916-T01-IM6	Pancreatic neuroendocrine tumor	Metastasis	TET1	p.K241E	FALSE	10	70332816	A	G	Missense_Mutation	0,277777778	828	FALSE	1	Clonal
P-0029916-T01-IM6	Pancreatic neuroendocrine tumor	Metastasis	MEN1	p.E45D	FALSE	11	64577447	C	G	Missense_Mutation	0,352173913	690	TRUE	1	Clonal
P-0029916-T01-IM6	Pancreatic neuroendocrine tumor	Metastasis	MAX	p.X22_splice	FALSE	14	65560535	T	C	Splice_Site	0,358208955	335	FALSE	1	Clonal
P-0030377-T01-IM6	Pancreatic neuroendocrine tumor	Metastasis	SPOP	p.D130E	FALSE	17	47696433	G	C	Missense_Mutation	0,370851371	693	FALSE	1	Clonal
P-0030377-T01-IM6	Pancreatic neuroendocrine tumor	Metastasis	VHL	p.V194Wfs*4	FALSE	3	10191587	GTGCAGAAAGACC	-	Frame_Shift_Del	0,722457627	472	TRUE	1	Clonal
P-0031225-T01-IM6	Pancreatic neuroendocrine tumor	Metastasis	BCOR	p.N1459S	TRUE	X	39921444	T	C	Missense_Mutation	0,68297456	511	FALSE	1	Clonal

Table S2

Sample ID	Cancer subtype	Sample type	Symbol	Aminoacid change	Hotspot	Chromosome	Position	Reference	Alternate	Mutation type	Tumor MAF	Tumor DP	LOH	Cancer cell fraction	Clonal status
P-0031225-T01-IM6	Pancreatic neuroendocrine tumor	Metastasis	PMS2	p.H479Q	FALSE	7	6026959	G	C	Missense_Mutation	0,069364162	173	FALSE	0.21	Subclonal
P-0031225-T01-IM6	Pancreatic neuroendocrine tumor	Metastasis	PPP2R1A	p.M350V	FALSE	19	52719836	A	G	Missense_Mutation	0,342719228	1243	FALSE	1	Clonal
P-0032869-T01-IM6	Pancreatic neuroendocrine tumor	Metastasis	MPL	p.P200L	FALSE	1	43805149	C	T	Missense_Mutation	0,171232877	438	FALSE	0.46	Subclonal
P-0032869-T01-IM6	Pancreatic neuroendocrine tumor	Metastasis	PARP1	p.G482E	FALSE	1	226567721	C	T	Missense_Mutation	0,118451025	439	FALSE	0.32	Subclonal
P-0032869-T01-IM6	Pancreatic neuroendocrine tumor	Metastasis	RET	p.P560L	FALSE	10	43608331	C	T	Missense_Mutation	0,055809698	1093	FALSE	0.26	Subclonal
P-0032869-T01-IM6	Pancreatic neuroendocrine tumor	Metastasis	ARID5B	p.A281T	FALSE	10	63810754	G	A	Missense_Mutation	0,81570997	331	FALSE	1	Clonal
P-0032869-T01-IM6	Pancreatic neuroendocrine tumor	Metastasis	TET1	p.W1220*	FALSE	10	70406146	G	A	Nonsense_Mutation	0,075630252	476	FALSE	0.2	Subclonal
P-0032869-T01-IM6	Pancreatic neuroendocrine tumor	Metastasis	RPS6KA4	p.A292T	FALSE	11	64129442	G	A	Missense_Mutation	0,113871636	483	FALSE	0.31	Subclonal
P-0032869-T01-IM6	Pancreatic neuroendocrine tumor	Metastasis	MEN1	p.L268P	FALSE	11	64574687	A	G	Missense_Mutation	0,729064039	406	TRUE	1	Clonal
P-0032869-T01-IM6	Pancreatic neuroendocrine tumor	Metastasis	ERBB3	p.S300F	FALSE	12	56482351	C	T	Missense_Mutation	0,063380282	852	FALSE	0.3	Subclonal
P-0032869-T01-IM6	Pancreatic neuroendocrine tumor	Metastasis	ERBB3	p.G718D	FALSE	12	56490384	G	A	Missense_Mutation	0,077777778	630	FALSE	0.37	Subclonal
P-0032869-T01-IM6	Pancreatic neuroendocrine tumor	Metastasis	ERBB3	p.A1233V	FALSE	12	56495508	C	T	Missense_Mutation	0,054778555	858	FALSE	0.26	Subclonal
P-0032869-T01-IM6	Pancreatic neuroendocrine tumor	Metastasis	POLE	p.T2215I	FALSE	12	133202244	G	A	Missense_Mutation	0,060606061	825	FALSE	0.28	Subclonal
P-0032869-T01-IM6	Pancreatic neuroendocrine tumor	Metastasis	POLE	p.M1470I	FALSE	12	133220027	C	T	Missense_Mutation	0,070998797	831	FALSE	0.33	Subclonal
P-0032869-T01-IM6	Pancreatic neuroendocrine tumor	Metastasis	POLE	p.S121Qfs*82	FALSE	12	133256601	GA	-	Frame_Shift_Del	0,135842881	611	FALSE	0.64	Subclonal
P-0032869-T01-IM6	Pancreatic neuroendocrine tumor	Metastasis	LATS2	p.P256S	FALSE	13	21563153	G	A	Missense_Mutation	0,423033067	877	FALSE	1	Clonal
P-0032869-T01-IM6	Pancreatic neuroendocrine tumor	Metastasis	IRS2	p.G508S	FALSE	13	110436879	C	T	Missense_Mutation	0,09476662	707	FALSE	0.45	Subclonal
P-0032869-T01-IM6	Pancreatic neuroendocrine tumor	Metastasis	MGA	p.D582N	FALSE	15	41988952	G	A	Missense_Mutation	0,081690141	355	FALSE	0.22	Subclonal
P-0032869-T01-IM6	Pancreatic neuroendocrine tumor	Metastasis	TSC2	p.P1202L	FALSE	16	2130373	C	T	Missense_Mutation	0,133640553	434	TRUE	1	Clonal
P-0032869-T01-IM6	Pancreatic neuroendocrine tumor	Metastasis	ZFHX3	p.P2871L	FALSE	16	72827969	G	A	Missense_Mutation	0,112852665	638	FALSE	0.3	Subclonal
P-0032869-T01-IM6	Pancreatic neuroendocrine tumor	Metastasis	ZFHX3	p.A1409V	FALSE	16	72832355	G	A	Missense_Mutation	0,137349398	415	FALSE	0.37	Subclonal
P-0032869-T01-IM6	Pancreatic neuroendocrine tumor	Metastasis	ZFHX3	p.G249S	FALSE	16	72993300	C	T	Missense_Mutation	0,074484945	631	FALSE	0.2	Subclonal
P-0032869-T01-IM6	Pancreatic neuroendocrine tumor	Metastasis	AURKB	p.A329T	FALSE	17	8108239	C	T	Missense_Mutation	0,058954394	899	FALSE	0.28	Subclonal
P-0032869-T01-IM6	Pancreatic neuroendocrine tumor	Metastasis	CDK12	p.S318F	FALSE	17	37619277	C	T	Missense_Mutation	0,054365733	607	FALSE	0.26	Subclonal
P-0032869-T01-IM6	Pancreatic neuroendocrine tumor	Metastasis	ERBB2	p.A588V	FALSE	17	37873598	C	T	Missense_Mutation	0,063366337	1010	FALSE	0.3	Subclonal
P-0032869-T01-IM6	Pancreatic neuroendocrine tumor	Metastasis	RARA	p.G13D	FALSE	17	38487508	G	A	Missense_Mutation	0,055393586	686	FALSE	0.26	Subclonal
P-0032869-T01-IM6	Pancreatic neuroendocrine tumor	Metastasis	DOT1L	p.P758L	FALSE	19	2216629	C	T	Missense_Mutation	0,054933876	983	FALSE	0.26	Subclonal
P-0032869-T01-IM6	Pancreatic neuroendocrine tumor	Metastasis	INSR	p.E1017K	FALSE	19	7125503	C	T	Missense_Mutation	0,066585956	826	FALSE	0.31	Subclonal
P-0032869-T01-IM6	Pancreatic neuroendocrine tumor	Metastasis	DNMT1	p.V1344M	FALSE	19	10249152	C	T	Missense_Mutation	0,064200218	919	FALSE	0.3	Subclonal
P-0032869-T01-IM6	Pancreatic neuroendocrine tumor	Metastasis	JAK3	p.R613Q	FALSE	19	17946809	C	T	Missense_Mutation	0,057546145	921	FALSE	0.27	Subclonal
P-0032869-T01-IM6	Pancreatic neuroendocrine tumor	Metastasis	UPF1	p.A878T	FALSE	19	18974278	G	A	Missense_Mutation	0,079182631	783	FALSE	0.37	Subclonal
P-0032869-T01-IM6	Pancreatic neuroendocrine tumor	Metastasis	UPF1	p.T922I	FALSE	19	18974411	C	T	Missense_Mutation	0,060070671	566	FALSE	0.28	Subclonal
P-0032869-T01-IM6	Pancreatic neuroendocrine tumor	Metastasis	CIC	p.G1068D	FALSE	19	42796745	G	A	Missense_Mutation	0,058947368	950	FALSE	0.28	Subclonal
P-0032869-T01-IM6	Pancreatic neuroendocrine tumor	Metastasis	DNMT3A	p.A45T	FALSE	2	25523052	C	T	Missense_Mutation	0,101694915	531	FALSE	0.27	Subclonal
P-0032869-T01-IM6	Pancreatic neuroendocrine tumor	Metastasis	MSH6	p.Q1314*	FALSE	2	48033729	C	T	Nonsense_Mutation	0,100917431	327	FALSE	0.27	Subclonal
P-0032869-T01-IM6	Pancreatic neuroendocrine tumor	Metastasis	ERBB4	p.A1219T	FALSE	2	212248612	C	T	Missense_Mutation	0,078512397	484	TRUE	0.21	Subclonal
P-0032869-T01-IM6	Pancreatic neuroendocrine tumor	Metastasis	CTNNB1	p.G268E	FALSE	3	41267219	G	A	Missense_Mutation	0,054775281	712	FALSE	0.26	Subclonal
P-0032869-T01-IM6	Pancreatic neuroendocrine tumor	Metastasis	CTNNB1	p.L517F	FALSE	3	41275654	C	T	Missense_Mutation	0,060606061	726	FALSE	0.28	Subclonal
P-0032869-T01-IM6	Pancreatic neuroendocrine tumor	Metastasis	EPHA3	p.A804T	FALSE	3	89498438	G	A	Missense_Mutation	0,062794349	637	FALSE	0.3	Subclonal
P-0032869-T01-IM6	Pancreatic neuroendocrine tumor	Metastasis	EPHB1	p.E816K	FALSE	3	134960089	G	A	Missense_Mutation	0,097087379	515	FALSE	0.26	Subclonal
P-0032869-T01-IM6	Pancreatic neuroendocrine tumor	Metastasis	ATR	p.S250N	FALSE	3	142281495	C	T	Missense_Mutation	0,103646833	521	FALSE	0.28	Subclonal
P-0032869-T01-IM6	Pancreatic neuroendocrine tumor	Metastasis	PIK3CA	p.G804R	FALSE	3	178942603	G	A	Missense_Mutation	0,142276423	246	FALSE	0.38	Subclonal
P-0032869-T01-IM6	Pancreatic neuroendocrine tumor	Metastasis	WHSC1	p.G1364S	FALSE	4	1980628	G	A	Missense_Mutation	0,063380282	426	FALSE	0.3	Subclonal
P-0032869-T01-IM6	Pancreatic neuroendocrine tumor	Metastasis	KIT	p.D615N	FALSE	4	55594057	G	A	Missense_Mutation	0,080724876	607	FALSE	0.38	Subclonal
P-0032869-T01-IM6	Pancreatic neuroendocrine tumor	Metastasis	FBXW7	p.G654S	FALSE	4	153244197	C	T	Missense_Mutation	0,057716437	797	FALSE	0.27	Subclonal
P-0032869-T01-IM6	Pancreatic neuroendocrine tumor	Metastasis	TERT	p.G135R	FALSE	5	1294598	C	T	Missense_Mutation	0,071287129	505	FALSE	0.34	Subclonal
P-0032869-T01-IM6	Pancreatic neuroendocrine tumor	Metastasis	FGFR4	p.L409F	FALSE	5	176520306	C	T	Missense_Mutation	0,066350711	844	FALSE	0.31	Subclonal
P-0032869-T01-IM6	Pancreatic neuroendocrine tumor	Metastasis	IRF4	p.G323E	FALSE	6	401646	G	A	Missense_Mutation	0,118380062	321	TRUE	0.32	Subclonal
P-0032869-T01-IM6	Pancreatic neuroendocrine tumor	Metastasis	EPHA7	p.E876K	FALSE	6	93956610	C	T	Missense_Mutation	0,067885117	383	FALSE	0.18	Subclonal
P-0032869-T01-IM6	Pancreatic neuroendocrine tumor	Metastasis	FYN	p.G66S	FALSE	6	112041059	C	T	Missense_Mutation	0,060827251	411	FALSE	0.16	Subclonal
P-0032869-T01-IM6	Pancreatic neuroendocrine tumor	Metastasis	EGFR	p.L62F	TRUE	7	55210074	C	T	Missense_Mutation	0,073414905	899	FALSE	0.35	Subclonal
P-0032869-T01-IM6	Pancreatic neuroendocrine tumor	Metastasis	MET	p.H1321Y	FALSE	7	116435966	C	T	Missense_Mutation	0,057986871	914	FALSE	0.27	Subclonal
P-0032869-T01-IM6	Pancreatic neuroendocrine tumor	Metastasis	RHEB	p.G177D	FALSE	7	151164230	C	T	Missense_Mutation	0,060498221	281	FALSE	0.28	Subclonal
P-0032869-T01-IM6	Pancreatic neuroendocrine tumor	Metastasis	PREX2	p.T289I	FALSE	8	68956748	C	T	Missense_Mutation	0,055950266	1126	FALSE	0.32	Subclonal
P-0032869-T01-IM6	Pancreatic neuroendocrine tumor	Metastasis	NTRK2	p.P769L	FALSE	9	87635254	C	T	Missense_Mutation	0,068278805	703	FALSE	0.32	Subclonal
P-0032869-T01-IM6	Pancreatic neuroendocrine tumor	Metastasis	KLF4	p.P160L	FALSE	9	110250196	G	A	Missense_Mutation	0,082245431	766	FALSE	0.39	Subclonal
P-0032869-T01-IM6	Pancreatic neuroendocrine tumor	Metastasis	NOTCH1	p.X1880_splice	FALSE	9	139395300	C	T	Splice_Site	0,077844311	668	FALSE	0.37	Subclonal
P-0032869-T01-IM6	Pancreatic neuroendocrine tumor	Metastasis	ARAF	p.G11E	FALSE	X	47422398	G	A	Missense_Mutation	0,111842105	304	FALSE	0.37	Subclonal
P-0032869-T01-IM6	Pancreatic neuroendocrine tumor	Metastasis	KDM5C	p.T463I	FALSE	X	53240692	G	A	Missense_Mutation	0,087912088	455	FALSE	0.37	Subclonal
P-0032869-T01-IM6	Pancreatic neuroendocrine tumor	Metastasis	STAG2	p.R654K	FALSE	X	123197837	G	A	Missense_Mutation	0,121287129	404	FALSE	0.32	Subclonal
P-0032869-T01-IM6	Pancreatic neuroendocrine tumor	Metastasis	PTPN11	p.A376V	FALSE	12	112919912	C	T	Missense_Mutation	0,068669528	233	FALSE	0.37	Subclonal
P-0032952-T01-IM6	Pancreatic neuroendocrine tumor	Metastasis	SETD2	p.E462Vfs*22	FALSE	3	47164722	TGTCTTCTTATACTCTTCTT	-	Frame_Shift_Del	0,711229947	374	TRUE	1	Clonal
P-0032952-T01-IM6	Pancreatic neuroendocrine tumor	Metastasis	DAXX	p.T639Qfs*6	FALSE	6	33287182	T	-	Frame_Shift_Del	0,727118644	590	TRUE	1	Clonal
P-0032979-T01-IM6	Pancreatic neuroendocrine tumor	Metastasis	CARD11	p.R555Gfs*45	FALSE	7	2968323	G	-	Frame_Shift_Del	0,192982456	684	FALSE	0.77	Subclonal
P-0032979-T01-IM6	Pancreatic neuroendocrine tumor	Metastasis	MEN1	p.R521Gfs*43	FALSE	11	64572093	G	-	Frame_Shift_Del	0,228733459	529	FALSE	0.92	Clonal
P-0032979-T01-IM6	Pancreatic neuroendocrine tumor	Metastasis	FGFR4	p.R758H	FALSE	5	176524541	G	A	Missense_Mutation	0,075129534	772	FALSE	0.3	Subclonal
P-0032979-T01-IM6	Pancreatic neuroendocrine tumor	Metastasis	TP53	p.V157I	TRUE	17	7578461	C	T	Missense_Mutation	0,204081633	686	FALSE	0.82	Clonal
P-0032979-T01-IM6	Pancreatic neuroendocrine tumor	Metastasis	RXRA	p.R421C	FALSE	9	137328332	C	T	Missense_Mutation	0,190551181	635	FALSE	0.76	Subclonal
P-0032979-T01-IM6	Pancreatic neuroendocrine tumor	Metastasis	RAD21	p.R586Q	FALSE	8	117859878	C	T	Missense_Mutation	0,103202847	281	FALSE	0.41	Subclonal
P-0032979-T01-IM6	Pancreatic neuroendocrine tumor	Metastasis	TSC2	p.A460V	FALSE	16	2112990	C	T	Missense_Mutation	0,106157113	471	FALSE	0.43	Subclonal
P-0032979-T01-IM6	Pancreatic neuroendocrine tumor	Metastasis	INPPL1	p.X708_splice	FALSE	11	71944567	G	A	Splice_Site	0,249496982	497	FALSE	1	Clonal
P-0032979-T01-IM6	Pancreatic neuroendocrine tumor	Metastasis	KDM5A	p.K1587Rfs*37	FALSE	12	402031	T	-	Frame_Shift_Del	0,167381974	466	FALSE	0.67	Subclonal
P-0032979-T01-IM6	Pancreatic neuroendocrine tumor	Metastasis	LATS2	p.A287T	FALSE	13	21563060	C	T	Missense_Mutation	0,076708508	717	FALSE	0.31	Subclonal
P-0032979-T01-IM6	Pancreatic neuroendocrine tumor	Metastasis	FLT1	p.G1086R	FALSE	13	28893590	C	T	Missense_Mutation	0,153061224	392	FALSE	0.61	Subclonal
P-0032979-T01-IM6	Pancreatic neuroendocrine tumor	Metastasis	NKX2-1	p.H90Afs*349	FALSE	14	36988389	-	A	Frame_Shift_Ins	0,244897959	294	FALSE	0.98	Clonal
P-0032979-T01-IM6	Pancreatic neuroendocrine tumor	Metastasis	TP53BP1	p.R1463C	FALSE	15	43712797	G	A	Missense_Mutation	0,263888889	576	FALSE	1	Clonal
P-0032979-T01-IM6	Pancreatic neuroendocrine tumor	Metastasis	TSC2	p.C230Y	FALSE	16	2106685	G	A	Missense_Mutation	0,237936772	601	FALSE	0.95	Clonal
P-0032979-T01-IM6	Pancreatic neuroendocrine tumor	Metastasis	TSC2	p.P1497S	FALSE	16	2134712	C	T	Missense_Mutation	0,082329317	498	FALSE	0.33	Subclonal
P-0032979-T01-IM6	Pancreatic neuroendocrine tumor	Metastasis	CDK12	p.R708H	FALSE	17	37649018	G	A	Missense_Mutation	0,074468085	282	FALSE	0.3	Subclonal
P-0032979-T01-IM6	Pancreatic neuroendocrine tumor	Metastasis	KMT2B	p.D1819N	FALSE	19	36222826	G	A	Missense_Mutation	0,122270742	687	FALSE	0.49	Subclonal
P-0032979-T01-IM6	Pancreatic neuroendocrine tumor	Metastasis	RRAS	p.E181K	FALSE	19	50139022	C	T	Missense_Mutation	0,136170213	705	FALSE	0.55	Subclonal

Table S2

Sample ID	Cancer subtype	Sample type	Symbol	Aminoacid change	Hotspot	Chromosome	Position	Reference	Alternate	Mutation type	Tumor MAF	Tumor DP	LOH	Cancer cell fraction	Clonal status
P-0032979-T01-IM6	Pancreatic neuroendocrine tumor	Metastasis	MSH2	p.G692R	FALSE	2	47703574	G	A	Missense_Mutation	0,280487805	492	FALSE	1	Clonal
P-0032979-T01-IM6	Pancreatic neuroendocrine tumor	Metastasis	KDR	p.G893D	FALSE	4	55962446	C	T	Missense_Mutation	0,066801619	494	FALSE	0.27	Subclonal
P-0032979-T01-IM6	Pancreatic neuroendocrine tumor	Metastasis	APC	p.G746S	FALSE	5	112173527	G	A	Missense_Mutation	0,072555205	317	FALSE	0.29	Subclonal
P-0032979-T01-IM6	Pancreatic neuroendocrine tumor	Metastasis	HIST1H3B	p.A89V	FALSE	6	26032023	G	A	Missense_Mutation	0,249027237	257	FALSE	1	Clonal
P-0032979-T01-IM6	Pancreatic neuroendocrine tumor	Metastasis	HLA-A	p.T187M	FALSE	6	29911261	C	T	Missense_Mutation	0,076086957	184	FALSE	0.3	Subclonal
P-0032979-T01-IM6	Pancreatic neuroendocrine tumor	Metastasis	DAXX	p.C240Y	FALSE	6	33288833	C	T	Missense_Mutation	0,210810811	370	TRUE	0.84	Clonal
P-0032979-T01-IM6	Pancreatic neuroendocrine tumor	Metastasis	ABL1	p.G754R	FALSE	9	133759937	G	A	Missense_Mutation	0,056	625	FALSE	0.22	Subclonal
P-0032979-T01-IM6	Pancreatic neuroendocrine tumor	Metastasis	KDM5C	p.P1253S	FALSE	X	53223602	G	A	Missense_Mutation	0,058252427	309	FALSE	0.22	Subclonal
P-0035509-T01-IM6	Pancreatic neuroendocrine tumor	Metastasis	MEN1	p.F452S	FALSE	11	64572516	A	G	Missense_Mutation	0,470394737	304	FALSE	1	Clonal
P-0035509-T01-IM6	Pancreatic neuroendocrine tumor	Metastasis	ATM	p.G2867E	FALSE	11	108218021	G	A	Missense_Mutation	0,419178082	365	FALSE	1	Clonal
P-0037474-T01-IM6	Pancreatic neuroendocrine tumor	Metastasis	MEN1	p.K135_H139del	FALSE	11	64577164	TGTGGGCCCGATCCT	-	In_Frame_Del	0,654205607	642	TRUE	1	Clonal
P-0037557-T01-IM6	Pancreatic neuroendocrine tumor	Metastasis	PBRM1	p.Y442*	FALSE	3	52663027	-	TATTCTTGATTCTTCAGTT	Nonsense_Mutation	0,170686456	539	FALSE	0.41	Subclonal
P-0038805-T01-IM6	Pancreatic neuroendocrine tumor	Metastasis	ASXL1	p.R1247H	FALSE	20	31024255	G	A	Missense_Mutation	0,385146805	579	FALSE	0.93	Clonal
P-0039419-T01-IM6	Pancreatic neuroendocrine tumor	Metastasis	TP53	p.S241Y	TRUE	17	7577559	G	T	Missense_Mutation	0,37917088	989	FALSE	1	Clonal
P-0039419-T01-IM6	Pancreatic neuroendocrine tumor	Metastasis	CREBBP	p.Y1482N	FALSE	16	3786767	A	T	Missense_Mutation	0,640684411	526	TRUE	1	Clonal
P-0039419-T01-IM6	Pancreatic neuroendocrine tumor	Metastasis	TCF7L2	p.C469Vfs*8	FALSE	10	114925325	-	G	Frame_Shift_Ins	0,496746204	461	FALSE	0.87	Clonal
P-0039419-T01-IM6	Pancreatic neuroendocrine tumor	Metastasis	SPOP	p.R138L	FALSE	17	47696410	C	A	Missense_Mutation	0,31981982	888	FALSE	0.88	Clonal
P-0039419-T01-IM6	Pancreatic neuroendocrine tumor	Metastasis	PTPRT	p.T370K	FALSE	20	41306550	G	T	Missense_Mutation	0,357300885	904	FALSE	0.99	Clonal
P-0039419-T01-IM6	Pancreatic neuroendocrine tumor	Metastasis	TP63	p.D83H	FALSE	3	189456486	G	C	Missense_Mutation	0,619164619	407	FALSE	1	Clonal
P-0039419-T01-IM6	Pancreatic neuroendocrine tumor	Metastasis	TET2	p.C828*	FALSE	4	106157583	C	A	Nonsense_Mutation	0,350917431	436	FALSE	0.97	Clonal
P-0039419-T01-IM6	Pancreatic neuroendocrine tumor	Metastasis	TCF7L2	p.K468R	FALSE	10	114925325	A	G	Missense_Mutation	0,074235808	458	FALSE	0.13	Subclonal
P-0041552-T01-IM6	Pancreatic neuroendocrine tumor	Metastasis	TRAF2	p.S304Tfs*11	FALSE	9	139814915	GCAGCCGGCA	-	Frame_Shift_Del	0,231852654	923	FALSE	0.79	Subclonal
P-0041570-T01-IM6	Pancreatic neuroendocrine tumor	Metastasis	MTOR	p.L2426V	FALSE	1	11174399	A	C	Missense_Mutation	0,279778393	722	FALSE	0.68	Subclonal
P-0041570-T01-IM6	Pancreatic neuroendocrine tumor	Metastasis	CDK12	p.H1035Lfs*6	FALSE	17	37680934	CA	-	Frame_Shift_Del	0,198852772	523	FALSE	0.48	Subclonal
P-0042403-T01-IM6	Pancreatic neuroendocrine tumor	Metastasis	KRAS	p.G12V	TRUE	12	25398284	C	A	Missense_Mutation	0,608208955	268	FALSE	1	Clonal
P-0042403-T01-IM6	Pancreatic neuroendocrine tumor	Metastasis	INSR	p.D247N	FALSE	19	7184562	C	T	Missense_Mutation	0,551807229	830	FALSE	1	Clonal
P-0042403-T01-IM6	Pancreatic neuroendocrine tumor	Metastasis	RB1	p.I753*	FALSE	13	49039179	A	-	Frame_Shift_Del	0,866013072	306	TRUE	1	Clonal
P-0042403-T01-IM6	Pancreatic neuroendocrine tumor	Metastasis	MGA	p.A2526S	FALSE	15	42054392	G	T	Missense_Mutation	0,312977099	131	FALSE	0.79	Subclonal
P-0042403-T01-IM6	Pancreatic neuroendocrine tumor	Metastasis	TP53	p.P177_H178del	FALSE	17	7578395	GGTGGG	-	In_Frame_Del	0,609907121	323	TRUE	0.94	Clonal
P-0042403-T01-IM6	Pancreatic neuroendocrine tumor	Metastasis	FAT1	p.S312N	FALSE	4	187630047	C	T	Missense_Mutation	0,705882353	238	TRUE	1	Clonal
P-0042403-T01-IM6	Pancreatic neuroendocrine tumor	Metastasis	RASA1	p.Y290S	FALSE	5	86629124	A	C	Missense_Mutation	0,427272727	110	FALSE	1	Clonal
P-0042403-T01-IM6	Pancreatic neuroendocrine tumor	Metastasis	NOTCH1	p.C1045Y	FALSE	9	139403359	C	T	Missense_Mutation	0,392857143	504	FALSE	1	Clonal
P-0043418-T01-IM6	Pancreatic neuroendocrine tumor	Metastasis	GNA11	p.R183C	FALSE	19	3115012	C	T	Missense_Mutation	0,444995045	1009	FALSE	1	Clonal
P-0043418-T01-IM6	Pancreatic neuroendocrine tumor	Metastasis	STAG2	p.R908G	FALSE	X	123211855	A	G	Missense_Mutation	0,606425703	249	FALSE	1	Clonal
P-0043725-T01-IM6	Pancreatic neuroendocrine tumor	Metastasis	VHL	p.R167W	FALSE	3	10191506	C	T	Missense_Mutation	0,621483376	391	TRUE	1	Clonal
P-0043725-T01-IM6	Pancreatic neuroendocrine tumor	Metastasis	WHSC1	p.E1099K	FALSE	4	1962801	G	A	Missense_Mutation	0,157657658	888	FALSE	0.44	Subclonal
P-0043725-T01-IM6	Pancreatic neuroendocrine tumor	Metastasis	PRKD1	p.A189V	FALSE	14	30133035	G	A	Missense_Mutation	0,358600583	686	FALSE	1	Clonal
P-0044031-T01-IM6	Pancreatic neuroendocrine tumor	Metastasis	MEN1	p.I97Mfs*19	FALSE	11	64577290	GG	-	Frame_Shift_Del	0,463917526	970	TRUE	1	Clonal
P-0044031-T01-IM6	Pancreatic neuroendocrine tumor	Metastasis	ERBB4	p.R1304Q	FALSE	2	212248356	C	T	Missense_Mutation	0,208571429	350	FALSE	1	Clonal
P-0044031-T01-IM6	Pancreatic neuroendocrine tumor	Metastasis	PBRM1	p.X79_splice	FALSE	3	52702661	TC	-	Splice_Site	0,398340249	241	FALSE	1	Clonal
P-0044031-T01-IM6	Pancreatic neuroendocrine tumor	Metastasis	FGFR1	p.R101Q	FALSE	8	38287355	C	T	Missense_Mutation	0,055464927	1226	FALSE	0.35	Subclonal
P-0044031-T01-IM6	Pancreatic neuroendocrine tumor	Metastasis	TSC1	p.K168*	FALSE	9	135798741	T	A	Nonsense_Mutation	0,491525424	236	TRUE	1	Clonal
P-0044178-T01-IM6	Pancreatic neuroendocrine tumor	Metastasis	BMPR1A	p.C67Y	FALSE	10	88649951	G	A	Missense_Mutation	0,098159509	163	FALSE	0.2	Subclonal
P-0044178-T01-IM6	Pancreatic neuroendocrine tumor	Metastasis	DAXX	p.P540Lfs*11	FALSE	6	33287465	ATCTATGCTGGAGGGG	-	Frame_Shift_Del	0,135802469	162	FALSE	0.28	Subclonal
P-0044178-T01-IM6	Pancreatic neuroendocrine tumor	Metastasis	MEN1	p.D253_L259delinsM	FALSE	11	64575047	GCGAGTCGGTGTGCAGGTC	T	In_Frame_Del	0,06231454	337	FALSE	0.13	Subclonal
P-0044792-T01-IM6	Pancreatic neuroendocrine tumor	Metastasis	TP53	p.R273C	TRUE	17	7577121	G	A	Missense_Mutation	0,944700461	1302	TRUE	1	Clonal
P-0044792-T01-IM6	Pancreatic neuroendocrine tumor	Metastasis	ERBB3	p.V104M	FALSE	12	56478854	G	A	Missense_Mutation	0,289772727	880	TRUE	0.7	Subclonal
P-0044792-T01-IM6	Pancreatic neuroendocrine tumor	Metastasis	ARID1A	p.Q412*	FALSE	1	27056238	C	T	Nonsense_Mutation	0,890092879	646	TRUE	1	Clonal
P-0044792-T01-IM6	Pancreatic neuroendocrine tumor	Metastasis	MAP3K13	p.L286*	FALSE	3	185165579	-	TTAAAGTG	Nonsense_Mutation	0,091503268	306	FALSE	0.4	Subclonal
P-0046277-T01-IM6	Pancreatic neuroendocrine tumor	Metastasis	KRAS	p.G12V	TRUE	12	25398284	C	A	Missense_Mutation	0,734767025	558	FALSE	1	Clonal
P-0046277-T01-IM6	Pancreatic neuroendocrine tumor	Metastasis	TP53	p.R282W	TRUE	17	7577094	G	A	Missense_Mutation	0,491111111	450	FALSE	1	Clonal
P-0046277-T01-IM6	Pancreatic neuroendocrine tumor	Metastasis	ARID1A	p.C1099Sfs*5	FALSE	1	27097705	GT	-	Frame_Shift_Del	0,187633262	469	FALSE	0.93	Clonal
P-0046277-T01-IM6	Pancreatic neuroendocrine tumor	Metastasis	ARID1A	p.N2059Kfs*76	FALSE	1	27106566	C	-	Frame_Shift_Del	0,398245614	570	FALSE	1	Clonal
P-0047236-T01-IM6	Pancreatic neuroendocrine tumor	Metastasis	FAT1	p.T3339N	FALSE	4	187531007	G	T	Missense_Mutation	0,304964539	282	FALSE	0.74	Subclonal
P-0048010-T01-IM6	Pancreatic neuroendocrine tumor	Metastasis	EIF1AX	p.G9R	TRUE	X	20156732	C	G	Missense_Mutation	0,3802589	618	FALSE	1	Clonal
P-0049898-T01-IM6	Pancreatic neuroendocrine tumor	Metastasis	PTEN	p.D24H	TRUE	10	89624296	G	C	Missense_Mutation	0,335616438	146	TRUE	0.99	Clonal
P-0049898-T01-IM6	Pancreatic neuroendocrine tumor	Metastasis	MEN1	p.X266_splice	FALSE	11	64575023	C	T	Splice_Site	0,316498316	297	TRUE	0.93	Clonal
P-0049898-T01-IM6	Pancreatic neuroendocrine tumor	Metastasis	SETD2	p.Q1931*	FALSE	3	47125479	G	A	Nonsense_Mutation	0,318181818	88	FALSE	0.94	Clonal
P-0049898-T01-IM6	Pancreatic neuroendocrine tumor	Metastasis	DAXX	p.T719Nfs*22	FALSE	6	33286781	-	T	Frame_Shift_Ins	0,246305419	203	TRUE	0.72	Subclonal
P-0045817-T01-IM6	Pancreatic neuroendocrine tumor	Metastasis	MEN1	p.A169V	FALSE	11	64575526	G	A	Missense_Mutation	0,436210131	1066	TRUE	1	Clonal
P-0045817-T01-IM6	Pancreatic neuroendocrine tumor	Metastasis	PTEN	p.X160_splice	FALSE	10	89692994	ACCAGAGACAAAAAGGTAAGTTAT	-	Splice_Site	0,328313253	332	TRUE	0.79	Subclonal

Table S3: Genetic somatic alterations identified in primary pancreatic neuroendocrine tumors and matched metastases subjected to targeted massively parallel sequencing.

Sample ID	Cancer subtype	Sample type	Symbol	Aminoacid change	Hotspot	Chromosome/ Cytoband	Position	Reference	Alternate	Mutation type	Private/shared	Tumor MAF	Tumor DP	LOH	Cancer cell fraction	Clonal status
Case 1	Pancreatic neuroendocrine tumor	Primary	DAXX	Q193*	FALSE	6	33288975	G	A	Nonsense_Mutation	Shared	0.269662921348315	445	TRUE	1	Clonal
Case 1	Pancreatic neuroendocrine tumor	Primary	MEN1	X263_splice	FALSE	11	64575021	CACCTGCTGCAGCTG	-	Splice_Site	Shared	0.239344262295082	305	TRUE	1	Clonal
Case 1	Pancreatic neuroendocrine tumor	Metastasis	DAXX	Q193*	FALSE	6	33288975	G	A	Nonsense_Mutation	Shared	0.734741784037559	426	TRUE	1	Clonal
Case 1	Pancreatic neuroendocrine tumor	Metastasis	MEN1	X263_splice	FALSE	11	64575021	CACCTGCTGCAGCTG	-	Splice_Site	Shared	0.752	375	TRUE	**1**	Clonal
Case 1	Pancreatic neuroendocrine tumor	Metastasis	CDKN2A	**.**	FALSE	9p21.3	.	.	.	Homozygous_deletion	Private	.	.	FALSE	.	.
Case 1	Pancreatic neuroendocrine tumor	Metastasis	CDKN2B	**.**	FALSE	9p21.3	.	.	.	Homozygous_deletion	Private	.	.	FALSE	.	.
Case 2	Pancreatic neuroendocrine tumor	Primary	ARID1A	p.E1766*	FALSE	1	27105685	G	T	Nonsense_Mutation	Shared	0,394736842	494	FALSE	0.84	Clonal
Case 2	Pancreatic neuroendocrine tumor	Primary	DAXX	p.E454*	FALSE	6	33287893	C	A	Nonsense_Mutation	Shared	0,366666667	510	TRUE	0.78	Subclonal
Case 2	Pancreatic neuroendocrine tumor	Primary	DAXX	p.G250C	FALSE	6	33288804	C	A	Missense_Mutation	Shared	0,531453362	461	TRUE	1	Clonal
Case 2	Pancreatic neuroendocrine tumor	Primary	PTEN	p.G156W	FALSE	10	89692982	G	T	Missense_Mutation	Private	0,405511811	254	TRUE	0.76	Clonal
Case 2	Pancreatic neuroendocrine tumor	Primary	HRAS	p.A134S	FALSE	11	533503	C	A	Missense_Mutation	Shared	0,52866242	785	TRUE	1	Clonal
Case 2	Pancreatic neuroendocrine tumor	Primary	MEN1	p.E260*	FALSE	11	64575044	C	A	Nonsense_Mutation	Shared	0,525956284	732	TRUE	1	Clonal
Case 2	Pancreatic neuroendocrine tumor	Primary	DNAJB1	p.T114A	FALSE	19	14627730	T	C	Missense_Mutation	Private	0,16286645	1228	FALSE	0.51	Subclonal
Case 2	Pancreatic neuroendocrine tumor	Primary	AKT2	p.S398R	FALSE	19	40741239	G	T	Missense_Mutation	Shared	0,344455348	1019	FALSE	1	Clonal
Case 2	Pancreatic neuroendocrine tumor	Primary	KDM5C	p.E185D	FALSE	X	53246427	C	A	Missense_Mutation	Private	0,574850299	668	FALSE	0.88	Clonal
Case 2	Pancreatic neuroendocrine tumor	Metastasis	ARID1A	p.E1766*	FALSE	1	27105685	G	T	Nonsense_Mutation	Shared	0,5472103	466	TRUE	1	Clonal
Case 2	Pancreatic neuroendocrine tumor	Metastasis	DAXX	p.E454*	FALSE	6	33287893	C	A	Nonsense_Mutation	Shared	0,515358362	586	TRUE	1	Clonal
Case 2	Pancreatic neuroendocrine tumor	Metastasis	DAXX	p.G250C	FALSE	6	33288804	C	A	Missense_Mutation	Shared	0,529247911	359	TRUE	1	Clonal
Case 2	Pancreatic neuroendocrine tumor	Metastasis	HRAS	p.A134S	FALSE	11	533503	C	A	Missense_Mutation	Shared	0,511744966	596	TRUE	1	Clonal
Case 2	Pancreatic neuroendocrine tumor	Metastasis	MEN1	p.E260*	FALSE	11	64575044	C	A	Nonsense_Mutation	Shared	0,50955414	471	TRUE	1	Clonal
Case 2	Pancreatic neuroendocrine tumor	Metastasis	AKT2	p.S398R	FALSE	19	40741239	G	T	Missense_Mutation	Shared	0,335343788	829	FALSE	1	Clonal
Case 2	Pancreatic neuroendocrine tumor	Metastasis	CXCR4	p.L86H	FALSE	2	136873241	A	T	Missense_Mutation	Private	0,504310345	696	TRUE	1	Clonal
Case 2	Pancreatic neuroendocrine tumor	Metastasis	FGFR4	p.A444S	FALSE	5	176520485	G	T	Missense_Mutation	Private	0,346774194	744	FALSE	1	Clonal
Case 2	Pancreatic neuroendocrine tumor	Metastasis	E2F3	p.R216I	FALSE	6	20481578	G	T	Missense_Mutation	Private	0,513711152	547	TRUE	1	Clonal
Case 2	Pancreatic neuroendocrine tumor	Metastasis	KMT2C	p.S806Y	FALSE	7	151945102	G	T	Missense_Mutation	Private	0,111888112	286	FALSE	0.33	Subclonal
Case 2	Pancreatic neuroendocrine tumor	Metastasis	FGFR2	p.G613V	FALSE	10	123256071	C	A	Missense_Mutation	Private	0,496441281	562	TRUE	0.98	Clonal
Case 2	Pancreatic neuroendocrine tumor	Metastasis	TSC2	p.W1208C	FALSE	16	2131609	G	T	Missense_Mutation	Private	0,541044776	536	TRUE	1	Clonal
Case 2	Pancreatic neuroendocrine tumor	Metastasis	CDKN2A	.	FALSE	9p21.3	.	.	.	Homozygous_deletion	Private	.	.	FALSE	.	.
Case 2	Pancreatic neuroendocrine tumor	Metastasis	CDKN2B	.	FALSE	9p21.3	.	.	.	Homozygous_deletion	Private	.	.	FALSE	.	.
Case 3	Pancreatic neuroendocrine tumor	Primary	DAXX	p.N268S	FALSE	6	33288749	T	C	Missense_Mutation	Shared	0,077809798	347	FALSE	0.32	Subclonal
Case 3	Pancreatic neuroendocrine tumor	Primary	ATRX	p.H2260R	FALSE	X	76778800	T	C	Missense_Mutation	Shared	0,26146789	218	FALSE	1	Clonal
Case 3	Pancreatic neuroendocrine tumor	Primary	MEN1	p.Y328Tfs*45	FALSE	11	64573786	A	-	Frame_Shift_Del	Shared	0,136363636	264	FALSE	0.62	Subclonal
Case 3	Pancreatic neuroendocrine tumor	Metastasis 1	DAXX	p.N268S	FALSE	6	33288749	T	C	Missense_Mutation	Shared	0,743707094	437	TRUE	1	Clonal
Case 3	Pancreatic neuroendocrine tumor	Metastasis 1	ATRX	p.H2260R	FALSE	X	76778800	T	C	Missense_Mutation	Shared	0,724373576	439	TRUE	1	Clonal
Case 3	Pancreatic neuroendocrine tumor	Metastasis 1	MEN1	p.Y328Tfs*45	FALSE	11	64573786	A	-	Frame_Shift_Del	Shared	0,770061728	648	TRUE	1	Clonal
Case 3	Pancreatic neuroendocrine tumor	Metastasis 1	TSC2	p.X325_splice	FALSE	16	2108877	A	C	Splice_Site	Shared	0,782462057	593	TRUE	1	Clonal
Case 3	Pancreatic neuroendocrine tumor	Metastasis 1	CDKN2A	.	FALSE	9p21.3	.	.	.	Homozygous_deletion	Shared	.	.	FALSE	.	.
Case 3	Pancreatic neuroendocrine tumor	Metastasis 1	CDKN2B	.	FALSE	9p21.3	.	.	.	Homozygous_deletion	Shared	.	.	FALSE	.	.
Case 3	Pancreatic neuroendocrine tumor	Metastasis 2	ATRX	p.H2260R	FALSE	X	76778800	T	C	Missense_Mutation	Shared	0,723502304	434	FALSE	1	Clonal
Case 3	Pancreatic neuroendocrine tumor	Metastasis 2	KDM6A	p.V558I	FALSE	X	44922811	G	A	Missense_Mutation	Private	0,145199063	427	FALSE	0.66	Subclonal
Case 3	Pancreatic neuroendocrine tumor	Metastasis 2	KDM5C	p.T575I	FALSE	X	53239618	G	A	Missense_Mutation	Private	0,061688312	616	FALSE	0.34	Subclonal
Case 3	Pancreatic neuroendocrine tumor	Metastasis 2	AMER1	p.G1079D	FALSE	X	63409931	C	T	Missense_Mutation	Private	0,063768116	345	FALSE	0.34	Subclonal
Case 3	Pancreatic neuroendocrine tumor	Metastasis 2	MED12	p.W158*	FALSE	X	70339941	G	A	Nonsense_Mutation	Private	0,081967213	488	FALSE	0.61	Subclonal
Case 3	Pancreatic neuroendocrine tumor	Metastasis 2	MED12	p.V1216M	FALSE	X	70349234	G	A	Missense_Mutation	Private	0,063795853	627	FALSE	0.34	Subclonal
Case 3	Pancreatic neuroendocrine tumor	Metastasis 2	MED12	p.E1608K	FALSE	X	70354657	G	A	Missense_Mutation	Private	0,1536	625	FALSE	0.51	Subclonal
Case 3	Pancreatic neuroendocrine tumor	Metastasis 2	ATRX	p.P2218L	FALSE	X	76812968	G	A	Missense_Mutation	Private	0,104815864	353	FALSE	0.74	Subclonal
Case 3	Pancreatic neuroendocrine tumor	Metastasis 2	ATRX	p.E1897K	FALSE	X	76855911	C	T	Missense_Mutation	Private	0,066246057	317	FALSE	0.40	Subclonal
Case 3	Pancreatic neuroendocrine tumor	Metastasis 2	ATRX	p.P1528L	FALSE	X	76891522	G	A	Missense_Mutation	Private	0,080082136	487	FALSE	0.45	Subclonal
Case 3	Pancreatic neuroendocrine tumor	Metastasis 2	ATRX	p.E824K	FALSE	X	76938278	C	T	Missense_Mutation	Private	0,051162791	430	FALSE	0.35	Subclonal
Case 3	Pancreatic neuroendocrine tumor	Metastasis 2	BTK	p.T403I	FALSE	X	100611913	G	A	Missense_Mutation	Private	0,110280374	535	FALSE	0.66	Subclonal
Case 3	Pancreatic neuroendocrine tumor	Metastasis 2	BTK	p.S318F	FALSE	X	100613626	G	A	Missense_Mutation	Private	0,072147651	596	FALSE	0.39	Subclonal
Case 3	Pancreatic neuroendocrine tumor	Metastasis 2	XIAP	p.W73*	FALSE	X	123019731	G	A	Nonsense_Mutation	Private	0,092009685	413	FALSE	0.60	Subclonal
Case 3	Pancreatic neuroendocrine tumor	Metastasis 2	XIAP	p.X434_splice	FALSE	X	123040837	G	A	Splice_Site	Private	0,09469697	528	FALSE	0.65	Subclonal
Case 3	Pancreatic neuroendocrine tumor	Metastasis 2	STAG2	p.V231M	FALSE	X	123181227	G	A	Missense_Mutation	Private	0,063909774	266	FALSE	0.40	Subclonal
Case 3	Pancreatic neuroendocrine tumor	Metastasis 2	TP53	p.G245D	TRUE	17	7577547	C	T	Missense_Mutation	Private	0,033968517	1207	FALSE	0.29	Subclonal
Case 3	Pancreatic neuroendocrine tumor	Metastasis 2	MAP2K4	p.L105F	FALSE	17	11984767	C	T	Missense_Mutation	Private	0,058139535	516	FALSE	0.5	Subclonal
Case 3	Pancreatic neuroendocrine tumor	Metastasis 2	CASP8	p.S158F	FALSE	2	202131505	C	T	Missense_Mutation	Private	0,061810155	453	TRUE	0.34	Subclonal
Case 3	Pancreatic neuroendocrine tumor	Metastasis 2	TSC2	p.P1092L	FALSE	16	2129420	C	T	Missense_Mutation	Private	0,070934256	578	TRUE	0.39	Subclonal
Case 3	Pancreatic neuroendocrine tumor	Metastasis 2	DICER1	p.E1794K	FALSE	14	95557687	C	T	Missense_Mutation	Private	0,335981839	881	FALSE	1	Clonal
Case 3	Pancreatic neuroendocrine tumor	Metastasis 2	REL	p.G579E	FALSE	2	61149546	G	A	Missense_Mutation	Private	0,056930693	404	TRUE	0.32	Subclonal
Case 3	Pancreatic neuroendocrine tumor	Metastasis 2	NOTCH4	p.S79N	FALSE	6	32190503	C	T	Missense_Mutation	Private	0,051820728	714	TRUE	0.29	Subclonal
Case 3	Pancreatic neuroendocrine tumor	Metastasis 2	HIST1H3F	p.G133E	FALSE	6	26250436	C	T	Missense_Mutation	Private	0,066439523	587	FALSE	0.57	Subclonal
Case 3	Pancreatic neuroendocrine tumor	Metastasis 2	DOT1L	p.G1386D	FALSE	19	2226677	G	A	Missense_Mutation	Private	0,022160665	1083	FALSE	0.19	Subclonal
Case 3	Pancreatic neuroendocrine tumor	Metastasis 2	DAXX	p.N268S	FALSE	6	33288749	T	C	Missense_Mutation	Shared	0,610619469	452	TRUE	1	Clonal

Table S3

Sample ID	Cancer subtype	Sample type	Symbol	Aminoacid change	Hotspot	Chromosome/ Cytoband	Position	Reference	Alternate	Mutation type	Private/shared	Tumor MAF	Tumor DP	LOH	Cancer cell fraction	Clonal status
Case 3	Pancreatic neuroendocrine tumor	Metastasis 2	MEN1	p.Y328Tfs*45	FALSE	11	64573786	A	-	Frame_Shift_Del	Shared	0,662087912	728	TRUE	1	Clonal
Case 3	Pancreatic neuroendocrine tumor	Metastasis 2	FLT1	p.T210I	FALSE	13	29008242	G	A	Missense_Mutation	Private	0,050488599	614	FALSE	0.43	Subclonal
Case 3	Pancreatic neuroendocrine tumor	Metastasis 2	PARK2	p.E344K	FALSE	6	161969939	C	T	Missense_Mutation	Private	0,0688	625	TRUE	0.38	Subclonal
Case 3	Pancreatic neuroendocrine tumor	Metastasis 2	EPHA3	p.P64L	FALSE	3	89259047	C	T	Missense_Mutation	Private	0,119298246	285	TRUE	0.66	Subclonal
Case 3	Pancreatic neuroendocrine tumor	Metastasis 2	TET1	p.R114Q	FALSE	10	70332436	G	A	Missense_Mutation	Private	0,084254144	724	TRUE	0.47	Subclonal
Case 3	Pancreatic neuroendocrine tumor	Metastasis 2	TP63	p.P166S	FALSE	3	189526232	C	T	Missense_Mutation	Private	0,067080745	805	TRUE	0.37	Subclonal
Case 3	Pancreatic neuroendocrine tumor	Metastasis 2	KLF4	p.G169E	FALSE	9	110250169	C	T	Missense_Mutation	Private	0,083067093	939	FALSE	0.71	Subclonal
Case 3	Pancreatic neuroendocrine tumor	Metastasis 2	KMT2C	p.P2607S	FALSE	7	151874719	G	A	Missense_Mutation	Private	0,072261072	858	FALSE	0.62	Subclonal
Case 3	Pancreatic neuroendocrine tumor	Metastasis 2	TP53	p.P222L	FALSE	17	7578184	G	A	Missense_Mutation	Private	0,059724349	1306	FALSE	0.51	Subclonal
Case 3	Pancreatic neuroendocrine tumor	Metastasis 2	TSC2	p.X325_splice	FALSE	16	2108877	A	C	Splice_Site	Private	0,616666667	600	TRUE	1	Clonal
Case 3	Pancreatic neuroendocrine tumor	Metastasis 2	KDR	p.G976E	FALSE	4	55961013	C	T	Missense_Mutation	Private	0,388282026	1007	FALSE	1	Clonal
Case 3	Pancreatic neuroendocrine tumor	Metastasis 2	BARD1	p.T88I	FALSE	2	215657122	G	A	Missense_Mutation	Private	0,090909091	440	TRUE	0.5	Subclonal
Case 3	Pancreatic neuroendocrine tumor	Metastasis 2	MET	p.S637F	FALSE	7	116397538	C	T	Missense_Mutation	Private	0,056430446	762	FALSE	0.48	Subclonal
Case 3	Pancreatic neuroendocrine tumor	Metastasis 2	ATM	p.P1069S	FALSE	11	108143500	C	T	Missense_Mutation	Private	0,064853556	478	TRUE	0.36	Subclonal
Case 3	Pancreatic neuroendocrine tumor	Metastasis 2	CASP8	p.E109K	FALSE	2	202131357	G	A	Missense_Mutation	Private	0,093023256	473	TRUE	0.52	Subclonal
Case 3	Pancreatic neuroendocrine tumor	Metastasis 2	BCL2L11	p.E23K	FALSE	2	111881389	G	A	Missense_Mutation	Private	0,055655296	557	TRUE	0.31	Subclonal
Case 3	Pancreatic neuroendocrine tumor	Metastasis 2	TNFRSF14	p.S108N	FALSE	1	2491280	G	A	Missense_Mutation	Private	0,15128593	661	TRUE	0.84	Clonal
Case 3	Pancreatic neuroendocrine tumor	Metastasis 2	ERRFI1	p.G205E	FALSE	1	8074045	C	T	Missense_Mutation	Private	0,096385542	415	TRUE	0.53	Subclonal
Case 3	Pancreatic neuroendocrine tumor	Metastasis 2	MTOR	p.E1530K	FALSE	1	11206831	C	T	Missense_Mutation	Private	0,065217391	552	TRUE	0.36	Subclonal
Case 3	Pancreatic neuroendocrine tumor	Metastasis 2	SPEN	p.S291N	FALSE	1	16203164	G	A	Missense_Mutation	Private	0,052529183	514	TRUE	0.29	Subclonal
Case 3	Pancreatic neuroendocrine tumor	Metastasis 2	ARID1A	p.P776L	FALSE	1	27088718	C	T	Missense_Mutation	Private	0,102827763	778	TRUE	0.57	Subclonal
Case 3	Pancreatic neuroendocrine tumor	Metastasis 2	ARID1A	p.P1400L	FALSE	1	27100917	C	T	Missense_Mutation	Private	0,114180479	543	TRUE	0.63	Subclonal
Case 3	Pancreatic neuroendocrine tumor	Metastasis 2	ARID1A	p.T1987I	FALSE	1	27106349	C	T	Missense_Mutation	Private	0,618604651	645	TRUE	1	Clonal
Case 3	Pancreatic neuroendocrine tumor	Metastasis 2	CSF3R	p.T234I	FALSE	1	36938260	G	A	Missense_Mutation	Private	0,052186178	709	TRUE	0.29	Subclonal
Case 3	Pancreatic neuroendocrine tumor	Metastasis 2	MYCL	p.R270K	FALSE	1	40363420	C	T	Missense_Mutation	Private	0,063380282	710	TRUE	0.35	Subclonal
Case 3	Pancreatic neuroendocrine tumor	Metastasis 2	FUBP1	p.T398I	FALSE	1	78428606	G	A	Missense_Mutation	Private	0,06127451	408	TRUE	0.34	Subclonal
Case 3	Pancreatic neuroendocrine tumor	Metastasis 2	FAM46C	p.L292F	FALSE	1	118166364	C	T	Missense_Mutation	Private	0,07424594	431	TRUE	0.41	Subclonal
Case 3	Pancreatic neuroendocrine tumor	Metastasis 2	DDR2	p.A747T	FALSE	1	162746116	G	A	Missense_Mutation	Private	0,080882353	408	TRUE	0.45	Subclonal
Case 3	Pancreatic neuroendocrine tumor	Metastasis 2	PARP1	p.G98D	FALSE	1	226580009	C	T	Missense_Mutation	Private	0,063464837	583	TRUE	0.35	Subclonal
Case 3	Pancreatic neuroendocrine tumor	Metastasis 2	RET	p.T488I	FALSE	10	43606854	C	T	Missense_Mutation	Private	0,067123288	730	TRUE	0.37	Subclonal
Case 3	Pancreatic neuroendocrine tumor	Metastasis 2	ARID5B	p.A279T	FALSE	10	63810748	G	A	Missense_Mutation	Private	0,068265683	542	TRUE	0.38	Subclonal
Case 3	Pancreatic neuroendocrine tumor	Metastasis 2	TET1	p.P1596S	FALSE	10	70432764	C	T	Missense_Mutation	Private	0,063953488	516	TRUE	0.35	Subclonal
Case 3	Pancreatic neuroendocrine tumor	Metastasis 2	IGF2	p.G41D	FALSE	11	2161405	C	T	Missense_Mutation	Private	0,055865922	716	TRUE	0.31	Subclonal
Case 3	Pancreatic neuroendocrine tumor	Metastasis 2	WT1	p.D427N	FALSE	11	32414272	C	T	Missense_Mutation	Private	0,051056338	568	TRUE	0.28	Subclonal
Case 3	Pancreatic neuroendocrine tumor	Metastasis 2	RPS6KA4	p.E490K	FALSE	11	64136957	G	A	Missense_Mutation	Private	0,050092764	539	TRUE	0.28	Subclonal
Case 3	Pancreatic neuroendocrine tumor	Metastasis 2	RPS6KA4	p.S619N	FALSE	11	64137755	G	A	Missense_Mutation	Private	0,084791386	743	TRUE	0.47	Subclonal
Case 3	Pancreatic neuroendocrine tumor	Metastasis 2	PAK1	p.A14V	FALSE	11	77103525	G	A	Missense_Mutation	Private	0,107070707	495	TRUE	0.59	Subclonal
Case 3	Pancreatic neuroendocrine tumor	Metastasis 2	MRE11A	p.X339_splice	FALSE	11	94203636	C	T	Splice_Site	Private	0,08097166	247	TRUE	0.45	Subclonal
Case 3	Pancreatic neuroendocrine tumor	Metastasis 2	PGR	p.G125E	FALSE	11	100999428	C	T	Missense_Mutation	Private	0,14552737	749	TRUE	0.81	Subclonal
Case 3	Pancreatic neuroendocrine tumor	Metastasis 2	ATM	p.H945Y	FALSE	11	108139331	C	T	Missense_Mutation	Private	0,050890585	393	TRUE	0.28	Subclonal
Case 3	Pancreatic neuroendocrine tumor	Metastasis 2	ATM	p.C1831Y	FALSE	11	108173752	G	A	Missense_Mutation	Private	0,05	300	TRUE	0.28	Subclonal
Case 3	Pancreatic neuroendocrine tumor	Metastasis 2	SDHD	p.P28L	FALSE	11	111958611	C	T	Missense_Mutation	Private	0,116945107	419	TRUE	0.65	Subclonal
Case 3	Pancreatic neuroendocrine tumor	Metastasis 2	KDM5A	p.A1049T	FALSE	12	420122	C	T	Missense_Mutation	Private	0,065436242	1192	FALSE	0.56	Subclonal
Case 3	Pancreatic neuroendocrine tumor	Metastasis 2	ARID2	p.A1250T	FALSE	12	46245654	G	A	Missense_Mutation	Private	0,052870091	662	FALSE	0.45	Subclonal
Case 3	Pancreatic neuroendocrine tumor	Metastasis 2	KMT2D	p.R3719K	FALSE	12	49427332	C	T	Missense_Mutation	Private	0,070960699	916	FALSE	0.61	Subclonal
Case 3	Pancreatic neuroendocrine tumor	Metastasis 2	KMT2D	p.G2585E	FALSE	12	49433799	C	T	Missense_Mutation	Private	0,398648649	1332	FALSE	1	Clonal
Case 3	Pancreatic neuroendocrine tumor	Metastasis 2	KMT2D	p.P2197L	FALSE	12	49434963	G	A	Missense_Mutation	Private	0,443807339	872	FALSE	1	Clonal
Case 3	Pancreatic neuroendocrine tumor	Metastasis 2	KMT2D	p.D1458N	FALSE	12	49440438	C	T	Missense_Mutation	Private	0,050873197	1317	FALSE	0.43	Subclonal
Case 3	Pancreatic neuroendocrine tumor	Metastasis 2	KMT2D	p.P196S	FALSE	12	49447848	G	A	Missense_Mutation	Private	0,35173642	1123	FALSE	1	Clonal
Case 3	Pancreatic neuroendocrine tumor	Metastasis 2	SH2B3	p.G517R	FALSE	12	111885927	G	A	Missense_Mutation	Private	0,403314917	905	FALSE	1	Clonal
Case 3	Pancreatic neuroendocrine tumor	Metastasis 2	POLE	p.E1299K	FALSE	12	133226002	C	T	Missense_Mutation	Private	0,063029163	1063	FALSE	0.54	Subclonal
Case 3	Pancreatic neuroendocrine tumor	Metastasis 2	FLT1	p.G191S	FALSE	13	29008300	C	T	Missense_Mutation	Private	0,061688312	616	FALSE	0.53	Subclonal
Case 3	Pancreatic neuroendocrine tumor	Metastasis 2	FOXO1	p.P299S	FALSE	13	41134733	G	A	Missense_Mutation	Private	0,061093248	622	FALSE	0.52	Subclonal
Case 3	Pancreatic neuroendocrine tumor	Metastasis 2	NFKBIA	p.G99E	FALSE	14	35872936	C	T	Missense_Mutation	Private	0,063508065	992	FALSE	0.54	Subclonal
Case 3	Pancreatic neuroendocrine tumor	Metastasis 2	FOXA1	p.T317I	FALSE	14	38061039	G	A	Missense_Mutation	Private	0,078277886	511	FALSE	0.67	Subclonal
Case 3	Pancreatic neuroendocrine tumor	Metastasis 2	DICER1	p.D418N	FALSE	14	95590657	C	T	Missense_Mutation	Private	0,06741573	712	FALSE	0.58	Subclonal
Case 3	Pancreatic neuroendocrine tumor	Metastasis 2	IGF1R	p.A746T	FALSE	15	99465411	G	A	Missense_Mutation	Private	0,079219288	871	FALSE	0.68	Subclonal
Case 3	Pancreatic neuroendocrine tumor	Metastasis 2	TSC2	p.P666L	FALSE	16	2121835	C	T	Missense_Mutation	Private	0,098901099	637	TRUE	0.55	Subclonal
Case 3	Pancreatic neuroendocrine tumor	Metastasis 2	TSC2	p.P903L	FALSE	16	2126137	C	T	Missense_Mutation	Private	0,055282555	814	TRUE	0.31	Subclonal
Case 3	Pancreatic neuroendocrine tumor	Metastasis 2	TRAF7	p.W628*	FALSE	16	2226271	G	A	Nonsense_Mutation	Private	0,050420168	595	TRUE	0.28	Subclonal
Case 3	Pancreatic neuroendocrine tumor	Metastasis 2	CREBBP	p.S1754N	FALSE	16	3779787	C	T	Missense_Mutation	Private	0,064083458	671	TRUE	0.36	Subclonal
Case 3	Pancreatic neuroendocrine tumor	Metastasis 2	NUP93	p.G40E	FALSE	16	56782278	G	A	Missense_Mutation	Private	0,107575758	660	TRUE	0.6	Subclonal
Case 3	Pancreatic neuroendocrine tumor	Metastasis 2	NUP93	p.L584F	FALSE	16	56868658	C	T	Missense_Mutation	Private	0,056521739	460	TRUE	0.31	Subclonal
Case 3	Pancreatic neuroendocrine tumor	Metastasis 2	ZFHX3	p.T2109I	FALSE	16	72830255	G	A	Missense_Mutation	Private	0,071661238	307	TRUE	0.4	Subclonal
Case 3	Pancreatic neuroendocrine tumor	Metastasis 2	ZFHX3	p.A775T	FALSE	16	72991722	C	T	Missense_Mutation	Private	0,066037736	318	TRUE	0.37	Subclonal
Case 3	Pancreatic neuroendocrine tumor	Metastasis 2	ZFHX3	p.P427S	FALSE	16	72992766	G	A	Missense_Mutation	Private	0,131294964	556	TRUE	0.73	Subclonal

Table S3

Sample ID	Cancer subtype	Sample type	Symbol	Aminoacid change	Hotspot	Chromosome/ Cytoband	Position	Reference	Alternate	Mutation type	Private/shared	Tumor MAF	Tumor DP	LOH	Cancer cell fraction	Clonal status
Case 3	Pancreatic neuroendocrine tumor	Metastasis 2	PLCG2	p.P433L	FALSE	16	81934321	C	T	Missense_Mutation	Private	0,122562674	718	TRUE	0.68	Subclonal
Case 3	Pancreatic neuroendocrine tumor	Metastasis 2	ANKRD11	p.P1779L	FALSE	16	89347614	G	A	Missense_Mutation	Private	0,06685633	703	TRUE	0.37	Subclonal
Case 3	Pancreatic neuroendocrine tumor	Metastasis 2	ANKRD11	p.E735K	FALSE	16	89350747	C	T	Missense_Mutation	Private	0,09273183	798	TRUE	0.51	Subclonal
Case 3	Pancreatic neuroendocrine tumor	Metastasis 2	FANCA	p.V491M	FALSE	16	89849510	C	T	Missense_Mutation	Private	0,061657033	519	TRUE	0.34	Subclonal
Case 3	Pancreatic neuroendocrine tumor	Metastasis 2	TP53	p.E62K	FALSE	17	7579503	C	T	Missense_Mutation	Private	0,064250412	1214	FALSE	0.55	Subclonal
Case 3	Pancreatic neuroendocrine tumor	Metastasis 2	AURKB	p.X69_splice	FALSE	17	8110686	C	T	Splice_Site	Private	0,076115486	762	FALSE	0.65	Subclonal
Case 3	Pancreatic neuroendocrine tumor	Metastasis 2	NCOR1	p.G1340D	FALSE	17	15974856	C	T	Missense_Mutation	Private	0,058201058	756	FALSE	0.5	Subclonal
Case 3	Pancreatic neuroendocrine tumor	Metastasis 2	ERBB2	p.A180T	FALSE	17	37865669	G	A	Missense_Mutation	Private	0,065068493	1168	FALSE	0.56	Subclonal
Case 3	Pancreatic neuroendocrine tumor	Metastasis 2	STAT5A	p.S664N	FALSE	17	40460280	G	A	Missense_Mutation	Private	0,066009852	1015	FALSE	0.56	Subclonal
Case 3	Pancreatic neuroendocrine tumor	Metastasis 2	BRCA1	p.S1551F	FALSE	17	41226371	G	A	Missense_Mutation	Private	0,050094518	1058	FALSE	0.43	Subclonal
Case 3	Pancreatic neuroendocrine tumor	Metastasis 2	HOXB13	p.P32L	FALSE	17	46805861	G	A	Missense_Mutation	Private	0,388203018	729	FALSE	1	Clonal
Case 3	Pancreatic neuroendocrine tumor	Metastasis 2	RNF43	p.A169V	FALSE	17	56440712	G	A	Missense_Mutation	Private	0,410997204	1073	FALSE	1	Clonal
Case 3	Pancreatic neuroendocrine tumor	Metastasis 2	PPM1D	p.A71T	FALSE	17	58677986	G	A	Missense_Mutation	Private	0,06013363	449	FALSE	0.51	Subclonal
Case 3	Pancreatic neuroendocrine tumor	Metastasis 2	PPM1D	p.E111K	FALSE	17	58678106	G	A	Missense_Mutation	Private	0,082846004	1026	FALSE	0.71	Subclonal
Case 3	Pancreatic neuroendocrine tumor	Metastasis 2	SOX9	p.T460I	FALSE	17	70120377	C	T	Missense_Mutation	Private	0,084959094	1589	FALSE	0.73	Subclonal
Case 3	Pancreatic neuroendocrine tumor	Metastasis 2	MALT1	p.P12S	FALSE	18	56338909	C	T	Missense_Mutation	Private	0,090163934	122	FALSE	0.77	Subclonal
Case 3	Pancreatic neuroendocrine tumor	Metastasis 2	TCF3	p.S229N	FALSE	19	1622189	C	T	Missense_Mutation	Private	0,087737844	946	FALSE	0.75	Subclonal
Case 3	Pancreatic neuroendocrine tumor	Metastasis 2	PTPRS	p.G1282E	FALSE	19	5219399	C	T	Missense_Mutation	Private	0,062132662	1191	FALSE	0.53	Subclonal
Case 3	Pancreatic neuroendocrine tumor	Metastasis 2	PTPRS	p.R851C	FALSE	19	5223252	G	A	Missense_Mutation	Private	0,058128974	1101	FALSE	0.5	Subclonal
Case 3	Pancreatic neuroendocrine tumor	Metastasis 2	BRD4	p.V997M	FALSE	19	15353891	C	T	Missense_Mutation	Private	0,078516903	917	FALSE	0.67	Subclonal
Case 3	Pancreatic neuroendocrine tumor	Metastasis 2	BRD4	p.G631D	FALSE	19	15366263	C	T	Missense_Mutation	Private	0,067474048	1156	FALSE	0.58	Subclonal
Case 3	Pancreatic neuroendocrine tumor	Metastasis 2	JAK3	p.X481_splice	FALSE	19	17950285	C	T	Splice_Site	Private	0,071948998	1098	FALSE	0.61	Subclonal
Case 3	Pancreatic neuroendocrine tumor	Metastasis 2	MEF2B	p.E321V	FALSE	19	19256751	T	A	Missense_Mutation	Private	0,376175549	957	FALSE	1	Clonal
Case 3	Pancreatic neuroendocrine tumor	Metastasis 2	MEF2B	p.G254D	FALSE	19	19257372	C	T	Missense_Mutation	Private	0,079831933	952	FALSE	0.68	Subclonal
Case 3	Pancreatic neuroendocrine tumor	Metastasis 2	CEBPA	p.G96D	FALSE	19	33793034	C	T	Missense_Mutation	Private	0,420258621	464	FALSE	1	Clonal
Case 3	Pancreatic neuroendocrine tumor	Metastasis 2	KMT2B	p.P938S	FALSE	19	36213986	C	T	Missense_Mutation	Private	0,07265625	1280	FALSE	0.62	Subclonal
Case 3	Pancreatic neuroendocrine tumor	Metastasis 2	KMT2B	p.A1404T	FALSE	19	36218431	G	A	Missense_Mutation	Private	0,389570552	978	FALSE	1	Clonal
Case 3	Pancreatic neuroendocrine tumor	Metastasis 2	KMT2B	p.P1446S	FALSE	19	36218632	C	T	Missense_Mutation	Private	0,079584775	1156	FALSE	0.68	Subclonal
Case 3	Pancreatic neuroendocrine tumor	Metastasis 2	KMT2B	p.P1970L	FALSE	19	36223359	C	T	Missense_Mutation	Private	0,070953437	1353	FALSE	0.61	Subclonal
Case 3	Pancreatic neuroendocrine tumor	Metastasis 2	ASXL2	p.L1067F	FALSE	2	25966007	G	A	Missense_Mutation	Private	0,056921087	773	TRUE	0.32	Subclonal
Case 3	Pancreatic neuroendocrine tumor	Metastasis 2	ALK	p.G1356E	FALSE	2	29420414	C	T	Missense_Mutation	Private	0,066006601	606	TRUE	0.37	Subclonal
Case 3	Pancreatic neuroendocrine tumor	Metastasis 2	ALK	p.E994K	FALSE	2	29449875	C	T	Missense_Mutation	Private	0,059311981	843	TRUE	0.33	Subclonal
Case 3	Pancreatic neuroendocrine tumor	Metastasis 2	MSH6	p.E1214K	FALSE	2	48032840	G	A	Missense_Mutation	Private	0,567567568	481	TRUE	1	Clonal
Case 3	Pancreatic neuroendocrine tumor	Metastasis 2	INPP4A	p.D756N	FALSE	2	99182201	G	A	Missense_Mutation	Private	0,051771117	734	TRUE	0.29	Subclonal
Case 3	Pancreatic neuroendocrine tumor	Metastasis 2	SF3B1	p.R512K	FALSE	2	198269804	C	T	Missense_Mutation	Private	0,096336499	737	TRUE	0.53	Subclonal
Case 3	Pancreatic neuroendocrine tumor	Metastasis 2	IRS1	p.T774I	FALSE	2	227661134	G	A	Missense_Mutation	Private	0,633181126	657	TRUE	1	Clonal
Case 3	Pancreatic neuroendocrine tumor	Metastasis 2	IRS1	p.G361D	FALSE	2	227662373	C	T	Missense_Mutation	Private	0,052536232	552	TRUE	0.29	Subclonal
Case 3	Pancreatic neuroendocrine tumor	Metastasis 2	SRC	p.E489K	FALSE	20	36031636	G	A	Missense_Mutation	Private	0,066543438	1082	FALSE	0.57	Subclonal
Case 3	Pancreatic neuroendocrine tumor	Metastasis 2	NCOA3	p.P215S	FALSE	20	46256415	C	T	Missense_Mutation	Private	0,063371356	789	FALSE	0.54	Subclonal
Case 3	Pancreatic neuroendocrine tumor	Metastasis 2	CHEK2	p.G178E	FALSE	22	29121024	C	T	Missense_Mutation	Private	0,064971751	708	TRUE	0.36	Subclonal
Case 3	Pancreatic neuroendocrine tumor	Metastasis 2	EP300	p.Q1223*	FALSE	22	41556722	C	T	Nonsense_Mutation	Private	0,118126273	491	TRUE	0.66	Subclonal
Case 3	Pancreatic neuroendocrine tumor	Metastasis 2	RAF1	p.A7T	FALSE	3	12660202	C	T	Missense_Mutation	Private	0,076923077	585	TRUE	0.43	Subclonal
Case 3	Pancreatic neuroendocrine tumor	Metastasis 2	TGFBR2	p.A335T	FALSE	3	30713603	G	A	Missense_Mutation	Private	0,616702355	467	TRUE	1	Clonal
Case 3	Pancreatic neuroendocrine tumor	Metastasis 2	SETD2	p.R2048K	FALSE	3	47103803	C	T	Missense_Mutation	Private	0,067357513	579	TRUE	0.37	Subclonal
Case 3	Pancreatic neuroendocrine tumor	Metastasis 2	RHOA	p.G155E	FALSE	3	49397760	C	T	Missense_Mutation	Private	0,056583243	919	TRUE	0.31	Subclonal
Case 3	Pancreatic neuroendocrine tumor	Metastasis 2	MST1R	p.D1208N	FALSE	3	49928652	C	T	Missense_Mutation	Private	0,054161162	757	TRUE	0.3	Subclonal
Case 3	Pancreatic neuroendocrine tumor	Metastasis 2	MST1R	p.X1022_splice	FALSE	3	49932879	C	T	Splice_Site	Private	0,054676259	695	TRUE	0.3	Subclonal
Case 3	Pancreatic neuroendocrine tumor	Metastasis 2	MST1R	p.A756T	FALSE	3	49934241	C	T	Missense_Mutation	Private	0,059375	640	TRUE	0.33	Subclonal
Case 3	Pancreatic neuroendocrine tumor	Metastasis 2	MST1R	p.P160S	FALSE	3	49940565	G	A	Missense_Mutation	Private	0,053054662	622	TRUE	0.29	Subclonal
Case 3	Pancreatic neuroendocrine tumor	Metastasis 2	MITF	p.P492S	FALSE	3	70014310	C	T	Missense_Mutation	Private	0,119815668	651	TRUE	0.66	Subclonal
Case 3	Pancreatic neuroendocrine tumor	Metastasis 2	EPHB1	p.E245K	FALSE	3	134670822	G	A	Missense_Mutation	Private	0,08266129	496	TRUE	0.46	Subclonal
Case 3	Pancreatic neuroendocrine tumor	Metastasis 2	FOXL2	p.S238N	FALSE	3	138664852	C	T	Missense_Mutation	Private	0,111627907	215	TRUE	0.62	Subclonal
Case 3	Pancreatic neuroendocrine tumor	Metastasis 2	PDGFRA	p.P32S	FALSE	4	55127306	C	T	Missense_Mutation	Private	0,065057712	953	FALSE	0.56	Subclonal
Case 3	Pancreatic neuroendocrine tumor	Metastasis 2	KIT	p.D284N	FALSE	4	55569983	G	A	Missense_Mutation	Private	0,053701016	689	FALSE	0.46	Subclonal
Case 3	Pancreatic neuroendocrine tumor	Metastasis 2	KIT	p.D768N	FALSE	4	55598105	G	A	Missense_Mutation	Private	0,056680162	988	FALSE	0.48	Subclonal
Case 3	Pancreatic neuroendocrine tumor	Metastasis 2	KDR	p.G1284E	FALSE	4	55946328	C	T	Missense_Mutation	Private	0,063063063	444	FALSE	0.54	Subclonal
Case 3	Pancreatic neuroendocrine tumor	Metastasis 2	TET2	p.G523R	FALSE	4	106156666	G	A	Missense_Mutation	Private	0,065934066	728	FALSE	0.56	Subclonal
Case 3	Pancreatic neuroendocrine tumor	Metastasis 2	TET2	p.D905N	FALSE	4	106157812	G	A	Missense_Mutation	Private	0,064833006	509	FALSE	0.55	Subclonal
Case 3	Pancreatic neuroendocrine tumor	Metastasis 2	INPP4B	p.E42K	FALSE	4	143350338	C	T	Missense_Mutation	Private	0,056565657	495	FALSE	0.48	Subclonal
Case 3	Pancreatic neuroendocrine tumor	Metastasis 2	FBXW7	p.S142N	FALSE	4	153332531	C	T	Missense_Mutation	Private	0,050691244	651	FALSE	0.43	Subclonal
Case 3	Pancreatic neuroendocrine tumor	Metastasis 2	FBXW7	p.G21D	FALSE	4	153332894	C	T	Missense_Mutation	Private	0,37279597	794	FALSE	1	Clonal
Case 3	Pancreatic neuroendocrine tumor	Metastasis 2	FAT1	p.R4252W	FALSE	4	187517940	G	A	Missense_Mutation	Private	0,133751306	957	FALSE	1	Clonal
Case 3	Pancreatic neuroendocrine tumor	Metastasis 2	TERT	p.G715D	FALSE	5	1278898	C	T	Missense_Mutation	Private	0,053459119	954	FALSE	0.46	Subclonal
Case 3	Pancreatic neuroendocrine tumor	Metastasis 2	PDGFRB	p.G363D	FALSE	5	149512352	C	T	Missense_Mutation	Private	0,069164265	694	TRUE	0.38	Subclonal
Case 3	Pancreatic neuroendocrine tumor	Metastasis 2	FLT4	p.X1001_splice	FALSE	5	180045769	C	T	Splice_Site	Private	0,116202946	611	TRUE	0.64	Subclonal
Case 3	Pancreatic neuroendocrine tumor	Metastasis 2	FLT4	p.S171L	FALSE	5	180057226	G	A	Missense_Mutation	Private	0,069943289	529	TRUE	0.39	Subclonal

Table S3

Sample ID	Cancer subtype	Sample type	Symbol	Aminoacid change	Hotspot	Chromosome/ Cytoband	Position	Reference	Alternate	Mutation type	Private/shared	Tumor MAF	Tumor DP	LOH	Cancer cell fraction	Clonal status
Case 3	Pancreatic neuroendocrine tumor	Metastasis 2	E2F3	p.S107F	FALSE	6	20402783	C	T	Missense_Mutation	Private	0,080838323	334	TRUE	0.45	Subclonal
Case 3	Pancreatic neuroendocrine tumor	Metastasis 2	HIST1H3B	p.M1?	FALSE	6	26032286	C	T	Translation_Start_Site	Private	0,061728395	324	TRUE	0.34	Subclonal
Case 3	Pancreatic neuroendocrine tumor	Metastasis 2	NOTCH4	p.A1314V	FALSE	6	32169092	G	A	Missense_Mutation	Private	0,068535826	642	TRUE	0.38	Subclonal
Case 3	Pancreatic neuroendocrine tumor	Metastasis 2	NOTCH4	p.A572V	FALSE	6	32184953	G	A	Missense_Mutation	Private	0,073211314	601	TRUE	0.41	Subclonal
Case 3	Pancreatic neuroendocrine tumor	Metastasis 2	VEGFA	p.X226_splice	FALSE	6	43749825	G	A	Splice_Site	Private	0,060344828	464	TRUE	0.33	Subclonal
Case 3	Pancreatic neuroendocrine tumor	Metastasis 2	PNRC1	p.P172L	FALSE	6	89791128	C	T	Missense_Mutation	Private	0,094170404	223	TRUE	0.52	Subclonal
Case 3	Pancreatic neuroendocrine tumor	Metastasis 2	FYN	p.W429*	FALSE	6	111995811	C	T	Nonsense_Mutation	Private	0,114334471	586	TRUE	0.63	Subclonal
Case 3	Pancreatic neuroendocrine tumor	Metastasis 2	ESR1	p.P336S	FALSE	6	152265553	C	T	Missense_Mutation	Private	0,053484603	617	TRUE	0.3	Subclonal
Case 3	Pancreatic neuroendocrine tumor	Metastasis 2	ARID1B	p.P1225S	FALSE	6	157510898	C	T	Missense_Mutation	Private	0,097069597	546	TRUE	0.54	Subclonal
Case 3	Pancreatic neuroendocrine tumor	Metastasis 2	CARD11	p.R720Q	FALSE	7	2962378	C	T	Missense_Mutation	Private	0,062893082	954	FALSE	0.54	Subclonal
Case 3	Pancreatic neuroendocrine tumor	Metastasis 2	MET	p.A1221T	FALSE	7	116423386	G	A	Missense_Mutation	Private	0,062611807	559	FALSE	0.54	Subclonal
Case 3	Pancreatic neuroendocrine tumor	Metastasis 2	SOX17	p.Q13*	FALSE	8	55370735	C	T	Nonsense_Mutation	Private	0,057692308	520	TRUE	0.32	Subclonal
Case 3	Pancreatic neuroendocrine tumor	Metastasis 2	AGO2	p.G616S	FALSE	8	141551451	C	T	Missense_Mutation	Private	0,073359073	518	TRUE	0.41	Subclonal
Case 3	Pancreatic neuroendocrine tumor	Metastasis 2	JAK2	p.H944Y	FALSE	9	5090514	C	T	Missense_Mutation	Private	0,086513995	393	TRUE	0.48	Subclonal
Case 3	Pancreatic neuroendocrine tumor	Metastasis 2	CD274	p.G159D	FALSE	9	5462915	G	A	Missense_Mutation	Private	0,077142857	350	TRUE	0.43	Subclonal
Case 3	Pancreatic neuroendocrine tumor	Metastasis 2	PTPRD	p.Q425*	FALSE	9	8518118	G	A	Nonsense_Mutation	Private	0,095	600	TRUE	0.53	Subclonal
Case 3	Pancreatic neuroendocrine tumor	Metastasis 2	PAX5	p.S168F	FALSE	9	37002746	G	A	Missense_Mutation	Private	0,120171674	466	FALSE	0.67	Subclonal
Case 3	Pancreatic neuroendocrine tumor	Metastasis 2	NOTCH1	p.P2253S	FALSE	9	139391434	G	A	Missense_Mutation	Private	0,08202765	1085	FALSE	0.7	Subclonal
Case 3	Pancreatic neuroendocrine tumor	Metastasis 2	CDKN2A	.	FALSE	9p21.3	.	.	.	Homozygous_deletion	Shared	.	.	FALSE	.	.
Case 3	Pancreatic neuroendocrine tumor	Metastasis 2	CDKN2B	.	FALSE	9p21.3	.	.	.	Homozygous_deletion	Shared	.	.	FALSE	.	.
Case 4	Pancreatic neuroendocrine tumor	Primary	MEN1	p.D70Tfs*49	FALSE	11	64577375	G	-	Frame_Shift_Del	Shared	0,679591837	490	TRUE	1	Clonal
Case 4	Pancreatic neuroendocrine tumor	Primary	BRD4	p.S601L	FALSE	19	15366353	G	A	Missense_Mutation	Shared	0,075268817	558	FALSE	0.19	Subclonal
Case 4	Pancreatic neuroendocrine tumor	Primary	RICTOR	p.X782_splice	FALSE	5	38958622	C	T	Splice_Site	Private	0,066666667	285	FALSE	0.17	Subclonal
Case 4	Pancreatic neuroendocrine tumor	Primary	ATRX	p.F1241Lfs*7	FALSE	X	76937024	AA	-	Frame_Shift_Del	Shared	0,687116564	326	TRUE	1	Clonal
Case 4	Pancreatic neuroendocrine tumor	Primary	CDKN2A	.	FALSE	9p21.3	.	.	.	Homozygous_deletion	Shared	.	.	FALSE	.	.
Case 4	Pancreatic neuroendocrine tumor	Primary	CDKN2B	.	FALSE	9p21.3	.	.	.	Homozygous_deletion	Shared	.	.	FALSE	.	.
Case 4	Pancreatic neuroendocrine tumor	Metastasis	RPTOR	p.V1327M	FALSE	17	78938101	G	A	Missense_Mutation	Private	0,25633383	671	FALSE	0.94	Clonal
Case 4	Pancreatic neuroendocrine tumor	Metastasis	MEN1	p.D70Tfs*49	FALSE	11	64577375	G	-	Frame_Shift_Del	Shared	0,756660746	563	TRUE	1	Clonal
Case 4	Pancreatic neuroendocrine tumor	Metastasis	BRD4	p.S601L	FALSE	19	15366353	G	A	Missense_Mutation	Shared	0,217758985	473	FALSE	0.78	Subclonal
Case 4	Pancreatic neuroendocrine tumor	Metastasis	ATRX	p.F1241Lfs*7	FALSE	X	76937024	AA	-	Frame_Shift_Del	Shared	0,814814815	189	TRUE	1	Clonal
Case 4	Pancreatic neuroendocrine tumor	Metastasis	CDKN2A	.	FALSE	9p21.3	.	.	.	Homozygous_deletion	Shared	.	.	FALSE	.	.
Case 4	Pancreatic neuroendocrine tumor	Metastasis	CDKN2B	.	FALSE	9p21.3	.	.	.	Homozygous_deletion	Shared	.	.	FALSE	.	.
Case 5	Pancreatic neuroendocrine tumor	Metastasis	SETD2	p.Q1750*	FALSE	3	47129632	G	A	Nonsense_Mutation	Shared	0,179530201	596	FALSE	1	Clonal
Case 5	Pancreatic neuroendocrine tumor	Metastasis	SETD2	p.Q2139*	FALSE	3	47098859	G	A	Nonsense_Mutation	Shared	0,357142857	1050	FALSE	1	Clonal
Case 5	Pancreatic neuroendocrine tumor	Metastasis	B2M	.	FALSE	15q21.1	.	.	.	Homozygous_deletion	Shared	.	.	FALSE	.	.
Case 5	Pancreatic neuroendocrine tumor	Metastasis	PTPRD	.	FALSE	9p24.1-p23	.	.	.	Homozygous_deletion	Shared	.	.	FALSE	.	.
Case 5	Pancreatic neuroendocrine tumor	Metastasis	SPRED1	.	FALSE	15q14	.	.	.	Homozygous_deletion	Shared	.	.	FALSE	.	.
Case 5	Pancreatic neuroendocrine tumor	Metastasis	RAD51	.	FALSE	15q15.1	.	.	.	Homozygous_deletion	Shared	.	.	FALSE	.	.
Case 5	Pancreatic neuroendocrine tumor	Metastasis	MGA	.	FALSE	15q15	.	.	.	Homozygous_deletion	Shared	.	.	FALSE	.	.
Case 5	Pancreatic neuroendocrine tumor	Metastasis	TP53BP1	.	FALSE	15q15.3	.	.	.	Homozygous_deletion	Shared	.	.	FALSE	.	.
Case 5	Pancreatic neuroendocrine tumor	Metastasis	CD274	.	FALSE	9p24.1	.	.	.	Homozygous_deletion	Shared	.	.	FALSE	.	.
Case 5	Pancreatic neuroendocrine tumor	Metastasis	JAK2	.	FALSE	9p24.1	.	.	.	Homozygous_deletion	Shared	.	.	FALSE	.	.
Case 5	Pancreatic neuroendocrine tumor	Metastasis	PDCD1LG2	.	FALSE	9p24.1	.	.	.	Homozygous_deletion	Shared	.	.	FALSE	.	.
Case 5	Pancreatic neuroendocrine tumor	Metastasis	KNSTRN	.	FALSE	15q15.1	.	.	.	Homozygous_deletion	Shared	.	.	FALSE	.	.
Case 5	Pancreatic neuroendocrine tumor	Primary	TP53	p.R248W	TRUE	17	7577539	G	A	Missense_Mutation	Private	0,272143774	779	FALSE	0.58	Subclonal
Case 5	Pancreatic neuroendocrine tumor	Primary	SETD2	p.Q1750*	FALSE	3	47129632	G	A	Nonsense_Mutation	Shared	0,239273927	606	FALSE	0.48	Subclonal
Case 5	Pancreatic neuroendocrine tumor	Primary	SETD2	p.Q2139*	FALSE	3	47098859	G	A	Nonsense_Mutation	Shared	0,491746308	1151	FALSE	1	Clonal
Case 5	Pancreatic neuroendocrine tumor	Primary	BCOR	p.E994Dfs*27	FALSE	X	39931617	C	-	Frame_Shift_Del	Private	0,310909091	550	FALSE	0.60	Subclonal
Case 5	Pancreatic neuroendocrine tumor	Primary	B2M	.	FALSE	15q21.1	.	.	.	Homozygous_deletion	Shared	.	.	FALSE	.	.
Case 5	Pancreatic neuroendocrine tumor	Primary	PTPRD	.	FALSE	9p24.1-p23	.	.	.	Homozygous_deletion	Shared	.	.	FALSE	.	.
Case 5	Pancreatic neuroendocrine tumor	Primary	SPRED1	.	FALSE	15q14	.	.	.	Homozygous_deletion	Shared	.	.	FALSE	.	.
Case 5	Pancreatic neuroendocrine tumor	Primary	RAD51	.	FALSE	15q15.1	.	.	.	Homozygous_deletion	Shared	.	.	FALSE	.	.
Case 5	Pancreatic neuroendocrine tumor	Primary	MGA	.	FALSE	15q15	.	.	.	Homozygous_deletion	Shared	.	.	FALSE	.	.
Case 5	Pancreatic neuroendocrine tumor	Primary	TP53BP1	.	FALSE	15q15.3	.	.	.	Homozygous_deletion	Shared	.	.	FALSE	.	.
Case 5	Pancreatic neuroendocrine tumor	Primary	CD274	.	FALSE	9p24.1	.	.	.	Homozygous_deletion	Shared	.	.	FALSE	.	.
Case 5	Pancreatic neuroendocrine tumor	Primary	JAK2	.	FALSE	9p24.1	.	.	.	Homozygous_deletion	Shared	.	.	FALSE	.	.
Case 5	Pancreatic neuroendocrine tumor	Primary	PDCD1LG2	.	FALSE	9p24.1	.	.	.	Homozygous_deletion	Shared	.	.	FALSE	.	.
Case 5	Pancreatic neuroendocrine tumor	Primary	KNSTRN	.	FALSE	15q15.1	.	.	.	Homozygous_deletion	Shared	.	.	FALSE	.	.
Case 6	Pancreatic neuroendocrine tumor	Primary	MEN1	p.I85Yfs*32	FALSE	11	64577329	-	A	Frame_Shift_Ins	Shared	0,307407407	810	FALSE	1	Clonal
Case 6	Pancreatic neuroendocrine tumor	Primary	TSC2	p.I1614del	FALSE	16	2136370	CAT	-	In_Frame_Del	Shared	0,263452915	892	FALSE	0.86	Clonal
Case 6	Pancreatic neuroendocrine tumor	Primary	DAXX	p.Q383*	FALSE	6	33288261	G	A	Nonsense_Mutation	Shared	0,306954436	834	FALSE	1	Clonal
Case 6	Pancreatic neuroendocrine tumor	Metastasis	CDKN2A	p.H83N	TRUE	9	21971111	G	T	Missense_Mutation	Private	0,082627119	472	FALSE	0.4	Subclonal
Case 6	Pancreatic neuroendocrine tumor	Metastasis	TSC2	p.I1614del	FALSE	16	2136370	CAT	-	In_Frame_Del	Shared	0,489224138	464	FALSE	1	Clonal
Case 6	Pancreatic neuroendocrine tumor	Metastasis	DAXX	p.Q383*	FALSE	6	33288261	G	A	Nonsense_Mutation	Shared	0,482200647	618	FALSE	1	Clonal
Case 6	Pancreatic neuroendocrine tumor	Metastasis	RPTOR	p.L1115P	FALSE	17	78923321	T	C	Missense_Mutation	Private	0,092495637	573	FALSE	0.63	Subclonal
Case 6	Pancreatic neuroendocrine tumor	Metastasis	MEN1	p.I85Yfs*32	FALSE	11	64577329	-	A	Frame_Shift_Ins	Shared	0,54192229	489	FALSE	1	Clonal
